# Designed endocytosis-inducing proteins degrade targets and amplify signals

**DOI:** 10.1038/s41586-024-07948-2

**Published:** 2024-09-25

**Authors:** Buwei Huang, Mohamad Abedi, Green Ahn, Brian Coventry, Isaac Sappington, Cong Tang, Rong Wang, Thomas Schlichthaerle, Jason Z. Zhang, Yujia Wang, Inna Goreshnik, Ching Wen Chiu, Adam Chazin-Gray, Sidney Chan, Stacey Gerben, Analisa Murray, Shunzhi Wang, Jason O’Neill, Li Yi, Ronald Yeh, Ayesha Misquith, Anitra Wolf, Luke M. Tomasovic, Dan I. Piraner, Maria J. Duran Gonzalez, Nathaniel R. Bennett, Preetham Venkatesh, Maggie Ahlrichs, Craig Dobbins, Wei Yang, Xinru Wang, Danny D. Sahtoe, Dionne Vafeados, Rubul Mout, Shirin Shivaei, Longxing Cao, Lauren Carter, Lance Stewart, Jamie B. Spangler, Kole T. Roybal, Per Jr Greisen, Xiaochun Li, Gonçalo J. L. Bernardes, Carolyn R. Bertozzi, David Baker

**Affiliations:** 1 Department of Biochemistry, University of Washington, Seattle, WA USA; 2 Institute for Protein Design, University of Washington, Seattle, WA USA; 3 Department of Bioengineering, University of Washington, Seattle, WA USA; 4 Department of Chemistry, Stanford University, Stanford, CA USA; 5 Howard Hughes Medical Institute, University of Washington, Seattle, WA USA; 6 Instituto de Medicina Molecular João Lobo Antunes, Faculdade de Medicina, Universidade de Lisboa, Lisbon, Portugal; 7 Department of Molecular Genetics, University of Texas Southwestern Medical Center, Dallas, TX USA; 8 Novo Nordisk, Måløv, Denmark; 9 Departments of Biomedical Engineering and Chemical and Biomolecular Engineering, Johns Hopkins University, Baltimore, MD USA; 10 Medical Scientist Training Program, Johns Hopkins University School of Medicine, Baltimore, MD USA; 11 Department of Microbiology and Immunology, University of California San Francisco, San Francisco, CA USA; 12 Hubrecht Institute, Utrecht, The Netherlands; 13 Harvard Medical School, Harvard University, Boston, MA USA; 14 Division of Biology and Bioengineering, California Institute of Technology, Pasadena, CA USA; 15 School of Life Sciences, Westlake University, Hangzhou, China; 16 Yusuf Hamied Department of Chemistry, University of Cambridge, Cambridge, UK; 17 Howard Hughes Medical Institute, Stanford, CA USA; 18 Sarafan ChEM-H, Stanford University, Stanford, CA USA

**Keywords:** Protein design, Synthetic biology

## Abstract

Endocytosis and lysosomal trafficking of cell surface receptors can be triggered by endogenous ligands. Therapeutic approaches such as lysosome-targeting chimaeras^1,2^ (LYTACs) and cytokine receptor-targeting chimeras^3^ (KineTACs) have used this to target specific proteins for degradation by fusing modified native ligands to target binding proteins. Although powerful, these approaches can be limited by competition with native ligands and requirements for chemical modification that limit genetic encodability and can complicate manufacturing, and, more generally, there may be no native ligands that stimulate endocytosis through a given receptor. Here we describe computational design approaches for endocytosis-triggering binding proteins (EndoTags) that overcome these challenges. We present EndoTags for insulin-like growth factor 2 receptor (IGF2R) and asialoglycoprotein receptor (ASGPR), sortilin and transferrin receptors, and show that fusing these tags to soluble or transmembrane target protein binders leads to lysosomal trafficking and target degradation. As these receptors have different tissue distributions, the different EndoTags could enable targeting of degradation to different tissues. EndoTag fusion to a PD-L1 antibody considerably increases efficacy in a mouse tumour model compared to antibody alone. The modularity and genetic encodability of EndoTags enables AND gate control for higher-specificity targeted degradation, and the localized secretion of degraders from engineered cells. By promoting endocytosis, EndoTag fusion increases signalling through an engineered ligand–receptor system by nearly 100-fold. EndoTags have considerable therapeutic potential as targeted degradation inducers, signalling activators for endocytosis-dependent pathways, and cellular uptake inducers for targeted antibody–drug and antibody–RNA conjugates.

## Abstract (web summary)

Computationally designed genetically encoded proteins can be used to target surface proteins, thereby triggering endocytosis and subsequent intracellular degradation, activating signalling or increasing cellular uptake in specific tissues.

## Main

The endocytosis of many cell surface receptors is triggered by binding of their endogenous ligands, which can shift the conformational or oligomerization state of the receptor^4^ and induce receptor clustering and recruitment of adaptor proteins^5,6^. Native endocytosis-inducing ligands have been utilized to target extracellular and membrane proteins to the lysosome for degradation^1–3^. Although powerful, these approaches have the limitations that native ligands can trigger off-target signalling^3,7^, their binding sites may be occupied by existing ligands^8^, and instability and—in some cases—the need for modification can complicate manufacturing^9^. Bio-orthogonal inducers of endocytosis could have therapeutic utility for targeted degradation or for initiating signalling through pathways involving endocytosis^4^, and could provide powerful tools for investigating the association between cellular trafficking and receptor conformational and oligomerization state. Antibodies have been identified that stimulate endocytosis, but this can require considerable empirical screening for any target receptor^10,11^.

**Fig. 1:**
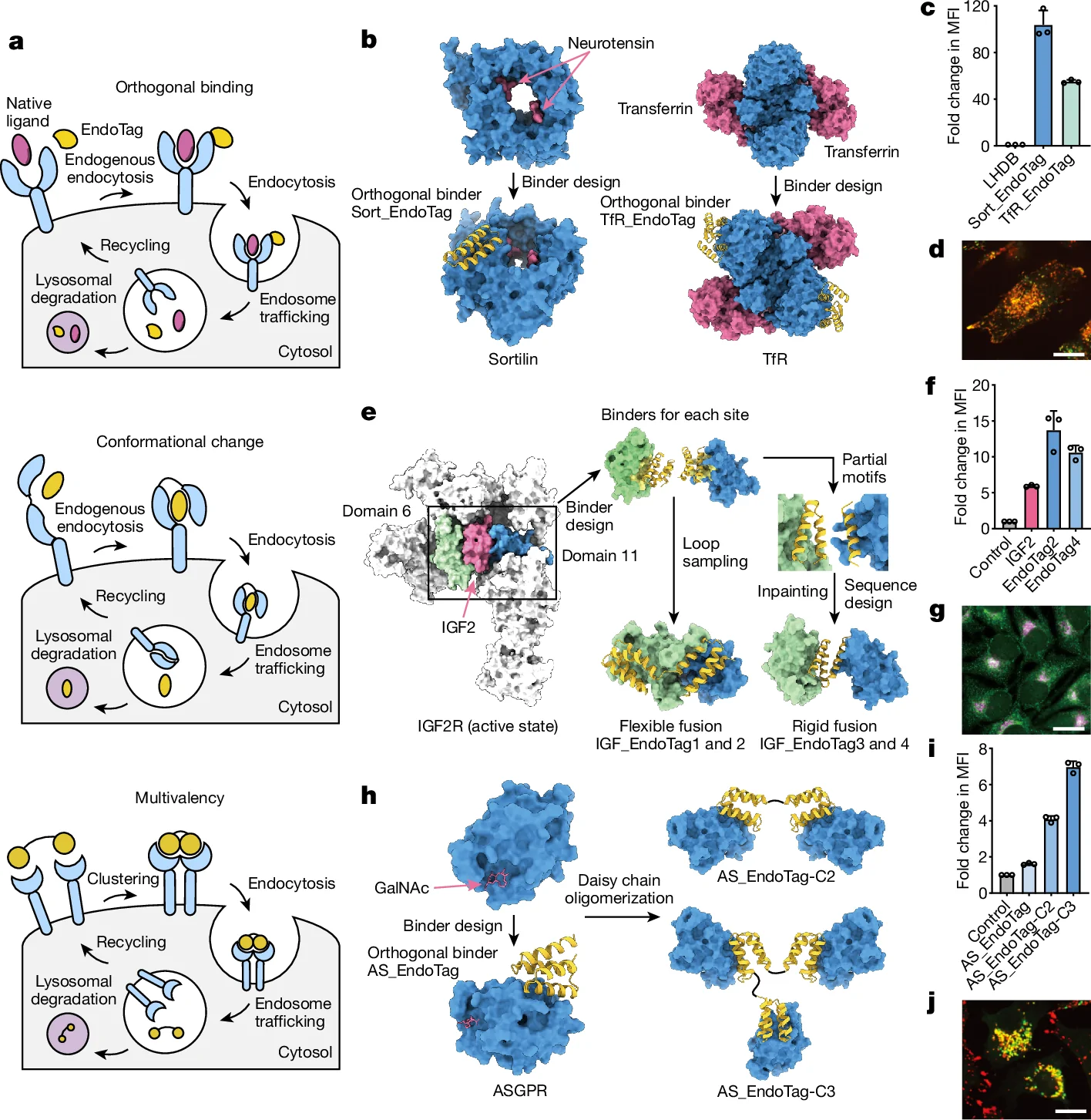
Design strategies for endocytosis-triggering EndoTags. **a**, Schema of designed endocytosis mechanisms. Top, design of binding to constitutively cycling receptors at sites that do not overlap with binding sites for natural ligands to avoid competition. Middle, design of binders that trigger endocytosis by eliciting conformational changes in the receptor. The EndoTag binds at two distinct epitopes on the target and actively triggers the conformational change. Bottom, designed endocytosis via receptor clustering. The multivalent EndoTag clusters multiple copies of the target receptor and induces endocytosis. **b**, Design strategy for sortilin^48^ and TfR^49^ EndoTags. **c**, Cellular uptake of 100 nM AF647-labelled Sort_EndoTags, TfR-EndoTags or LHDB^22^ scaffold control for 2 h in U-251MG cells. Data were normalized to the 100 nM AF647-labelled LHDB group (no endocytosis). MFI, mean fluorescence intensity. **d**, Confocal imaging of Sort_EndoTag (red) and lysosomal marker (green, AF488-labelled LysoTracker) after 24 h incubation in U-251MG cells. **e**, Design strategy for IGF_EndoTags. **f**, Cellular uptake of IGF_EndoTags in Jurkat cells with biotinylated 100 nM IGF_EndoTags or IGF2 and 33 nM Streptavidin–AF647 for 24 h. Data were normalized with the control group treated with 33 nM Streptavidin–AF647 alone. **g**, Fluorescence microscopy showing IGF_EndoTag1 (pink) co-localization with lysosomes (green, anti-LAMP2A) in HeLa cells. **h**, Design strategy for ASGPR EndoTags. **i**, Cellular uptake of ASGPR EndoTags. Hep3B cells were treated with 100 nM AF647-conjugated ASGPR EndoTags for 24 h followed with flow cytometry. Data were normalized to 100 nM AF647-labelled LHDB group (no endocytosis). **j**, Confocal imaging of AS_EndoTag (red) with lysosome (green, AF488-labelled LysoTracker); 500 nM AF647-labelled AS_EndoTag/AS_EndoTag-2C/AS_EndoTag-3C was incubated with Hep3B cells for 24 h. In **c**,**f**,**i**, data are mean ± s.e.m. of three biological replicates. In **d**,**g**,**j**, images are representative of three independently replicated samples. Scale bars, 20 µm.

We reasoned that de novo protein design could enable the creation of bio-orthogonal endocytosis-inducing proteins that avoid the above limitations using strategies customized for the target receptor. To enable tissue-specific control over endocytosis for downstream applications, we selected target receptors with distinct tissue expression profiles: IGF2R is expressed in most tissues, asialoglycoprotein receptor (ASGPR) is expressed primarily in the liver, transferrin receptor (TfR) is expressed in the brain, liver and muscles, and sortilin is expressed in the brain and spinal cord^12–14^. For receptors such as sortilin and TfR that constitutively traffic between the cell surface and the endosome–lysosome, binding to a site on the receptor that does not overlap with the native ligands could be sufficient (Fig. 1a). For receptors such as IGF2R, for which conformational change triggers endocytosis, binding must induce rearrangement of receptor extracellular domains, whereas for others, such as ASGPR, for which endocytosis is stimulated by clustering, binding should induce oligomerization. Fusion of designed proteins with these properties to a second target-binding protein could promote endocytosis and lysosomal trafficking of the target. We set out to design such endocytosis-targeting proteins, which we call EndoTags, for all four receptor systems (IGF2R, ASGPR, sortilin and TfR), and to explore their utility for modulating protein degradation and cellular signalling.

## EndoTags orthogonal to native ligands

TfR and sortilin constitutively cycle between the cell surface and intracellular compartments. Thus, for these receptors, the challenge is not to actively induce endocytosis, but to bind to the receptor at a site that does not compete for the natural ligand, which could have undesired side effects and reduce efficiency. De novo protein design has the advantage of being able to target binders to specific sites of interest on a target^15–17^, and is thus well suited to designing protein binders that target receptor sites that do not overlap with those of native ligands.

**Extended Data Fig. 1:**
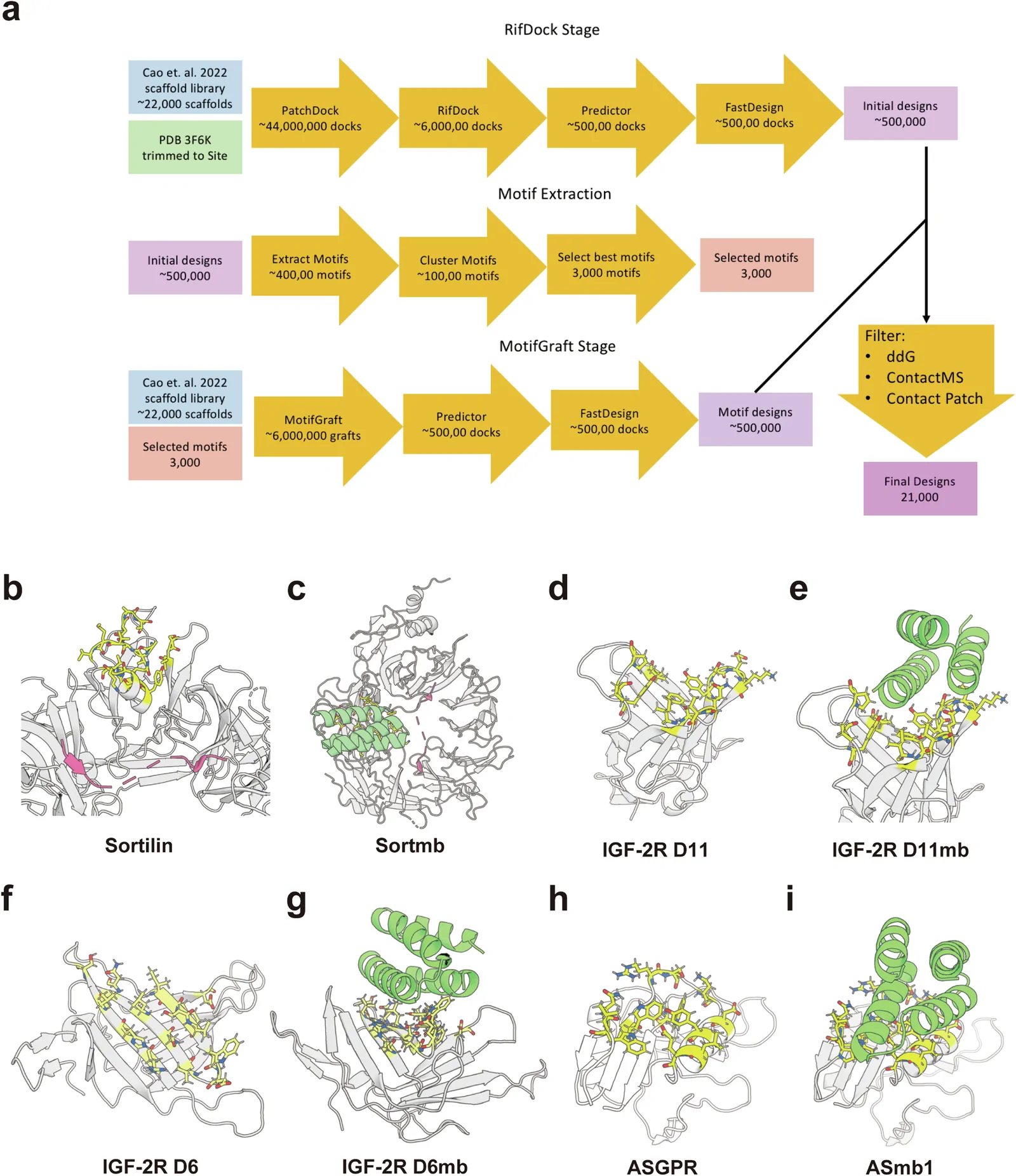
Binder design strategy and epitope selection. **a**, Rifdock-based binder design pipeline. **b**, Selected target region for IGF2R D6, yellow region highlighted the selected residues for rifdock. **c**, Design model for D6mb in complex with IGF2R D6. **d**, Selected target region for IGF2R D11, yellow region highlighted the selected residues for rifdock. **e**, Design model for D11mb in complex with IGF2R D11. **f**, Selected target region for Sortilin, yellow region highlighted the selected residues for rifdock. **g**, Design model for Sort_EndoTag (green) in complex with Sortilin. **h**, Selected target region for ASGPR orthogonal binding sites, yellow region highlighted the selected residues for rifdock. **i**, Design model for ASmb1 in complex with ASGPR.

**Extended Data Fig. 2:**
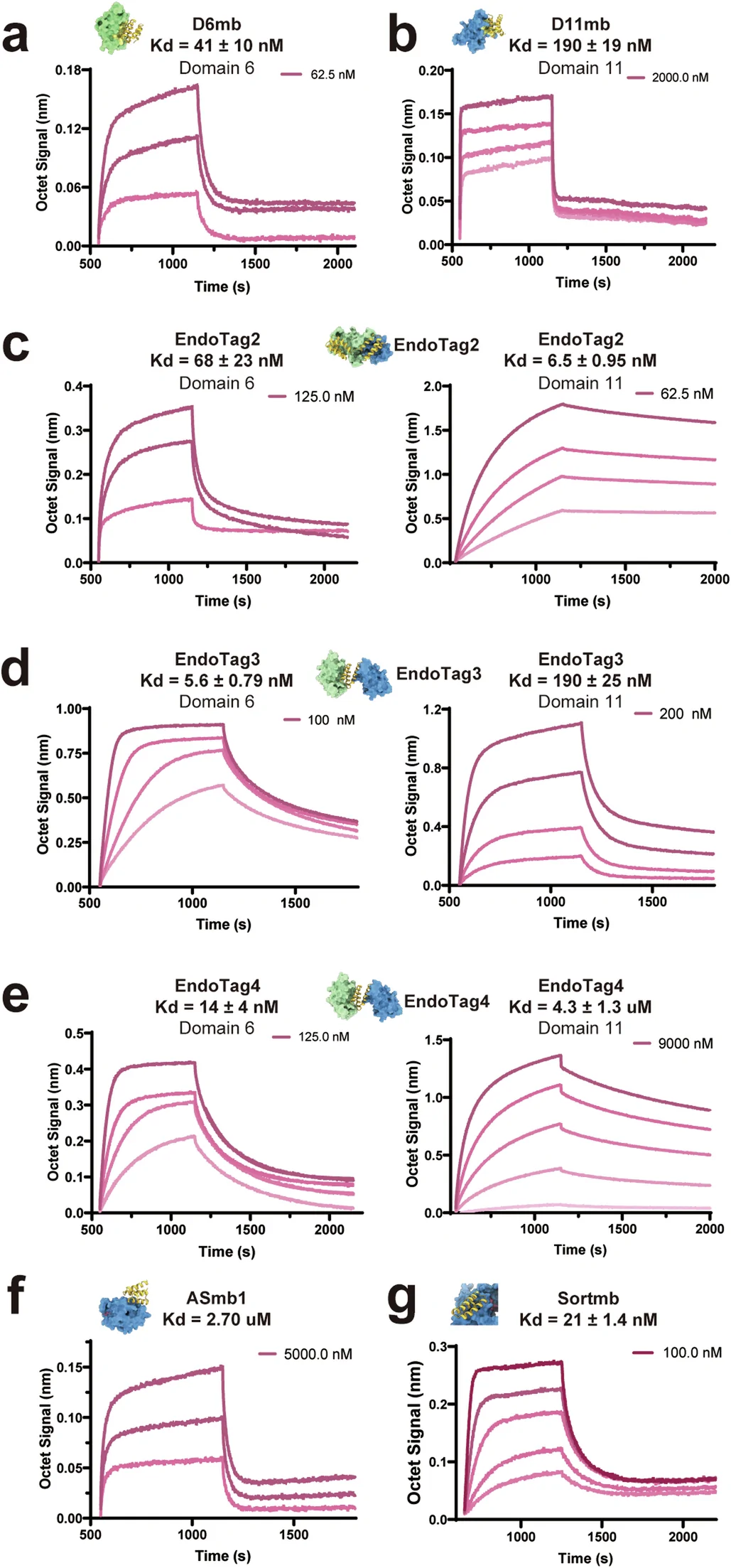
Binding affinity measurement for IGF2R and ASGPR minibinders. **a**, BLI binding affinity measurement for D6mb against IGF2R D6. **b**, BLI binding affinity measurement for D11mb against IGF2R D11. **c**, BLI binding affinity measurement for EndoTag2 against IGF2R D6 (left) and D11 (right). **d**, BLI binding affinity measurement for EndoTag3 against IGF2R D6 (left) and D11 (right). **e**, BLI binding affinity measurement for EndoTag4 against IGF2R D6 (left) and D11 (right). **f**, BLI binding affinity measurement for ASmb1 against ASGPR. **g**, BLI binding affinity measurement for Sort_EndoTag against Sortilin. All affinity data was collected by Octet R8 and binding affinity is estimated by Octet ForteBio software package.

Sortilin is a rapid trafficking receptor with substantial expression in the neural system, and has a role in lysosomal targeting of neurotensin^14,18^. We sought to design protein binders of sortilin at binding sites that do not overlap with native ligands, including neurotensin (Fig. 1a,b). We used Rosetta de novo binder design^15^ and yeast display (Extended Data Fig. 1a) to design and screen molecules that bind sortilin at a site that does not overlap with known interactions or undergo considerable structural change at low pH^18^ (Extended Data Fig. 1b,c); the highest affinity design (Sort_EndoTag) had a dissociation constant (*K*_d_) for Sortilin of 21 nM (Extended Data Fig. 2g and Extended Data Table 1). Four sortilin variants that introduce N-linked glycans close to the designed Sort_EndoTag interface blocked binding (Supplementary Fig. 19a–f), supporting the computational design model. Following incubation of 200 nM fluorescence-labelled Sort_EndoTag with U-251MG glioblastoma cells for 2 h at 37 °C followed by extensive washing, there was a 90-fold increase in fluorescence compared with fluorophore-conjugated control (Fig. 1c). Confocal imaging indicated co-localization of the Sort_EndoTag with a lysosomal marker after 24 h incubation in U-251MG cells (Fig. 1d and Supplementary Fig. 18a).

We applied a similar orthogonal binding strategy with TfR (Fig. 1b) whose native function is to transport iron-bound transferrin into cells and across the blood–brain barrier^13,19^, taking advantage of a previously designed binder that binds a site away from the transferrin-binding site^20^ and further optimized for solubility using ProteinMPNN^21^ (Supplementary Table 3). We found that this design, referred to here as TfR_EndoTag, was readily endocytosed in U-251MG glioblastoma cells, with a 50-fold increase in cellular uptake over the LHDB scaffold control^22^ after 2 h incubation (Fig. 1c). Confocal imaging again indicated lysosome targeting of the TfR_EndoTag after 24 h incubation in U-251MG cells (Supplementary Fig. 18b). Co-incubation of TfR_EndoTag with fluorescence-labelled transferrin had no effect on transferrin binding and uptake in HeLa cells (Extended Data Fig. 7d,e).

## Triggering conformational change

IGF2R rapidly transports the endogenous ligands IGF2 and mannose-6-phosphate (M6P) to the lysosome for degradation^1^. Structural analysis suggests that IGF2 binding induces a conformational change in IGF2R that brings together domain 6 (D6) and domain 11 (D11) and promotes dimerization of the receptor^23^. Using the ability to design de novo binders at arbitrary interfaces^15^, we hypothesized that a designed binding protein that brings together domain 6 and domain 11 could similarly trigger IGF2R endocytosis and lysosomal targeting without triggering off-target signalling activation, similar to IGF2^7^.

We used the Rosetta RIFdock method^15,16^ to design small proteins (minibinders) that bind to domain 6 and domain 11 (Extended Data Fig. 1d–g and Supplementary Methods). We expressed binding proteins identified by yeast display screening in *Escherichia coli* and measured the binding affinities using biolayer interferometry (BLI). The tightest binder to domain 6 (D6mb) had an affinity of 41 nM (Extended Data Fig. 2a and Extended Data Table 1), and the tightest binder to domain 11 (D11mb) had an affinity of 190 nM (Extended Data Fig. 2b and Extended Data Table 1), which was improved to 6.5 nM following optimization (D11mb2) (Extended Data Fig. 2c and Extended Data Table 1).

We next sought to develop IGF2R EndoTags (IGF_EndoTags) by fusing the domain 6 and domain 11 binders (Fig. 1e). We first explored flexible fusions between D11mb and D6mb, with different loop lengths and domain orders; we expressed these fusions in *E. coli* and, following conjugation with Alexa Fluor 647 (AF647), evaluated cellular uptake using flow cytometry. Treatment with a D11mb–GGS–D6mb fusion (IGF_EndoTag1; where GGS is a flexible Gly-Gly-Ser linker) resulted in increased cell-associated fluorescence over native IGF2 or D6mb or D11mb alone in Jurkat cells (Extended Data Fig. 4c). Fluorescence microscopy indicated that IGF_EndoTag1 is targeted to lysosomes (Fig. 1g), recapitulating the trafficking of endogenous IGF2 ligands. Longer linkers decreased the uptake level, whereas a shorter Gly-Ser (GS) linker abolished uptake (Extended Data Fig. 4b), suggesting that the orientation and distance of the two binding domains modulates IGF2R endocytosis. Constructs with two copies of one minibinder (D6mb–linker–D6mb and D11mb–linker–D11mb) were not taken up (Extended Data Fig. 4a); engagement of both domains (which probably drive their reorientation within the receptor structure) appears to be necessary to trigger efficient cellular uptake. Substitution of D11mb with the higher affinity variant D11mb2 in IGF_EndoTag1 (generating IGF_EndoTag2) increased internalization twofold compared with native IGF2 in Jurkat cells (Fig. 1f); IGF_EndoTag2, but not IGF2 or IGF_EndoTag1, was clearly detectable in lysosomes after a 30 min incubation (Extended Data Fig. 4d,e).

**Extended Data Fig. 3:**
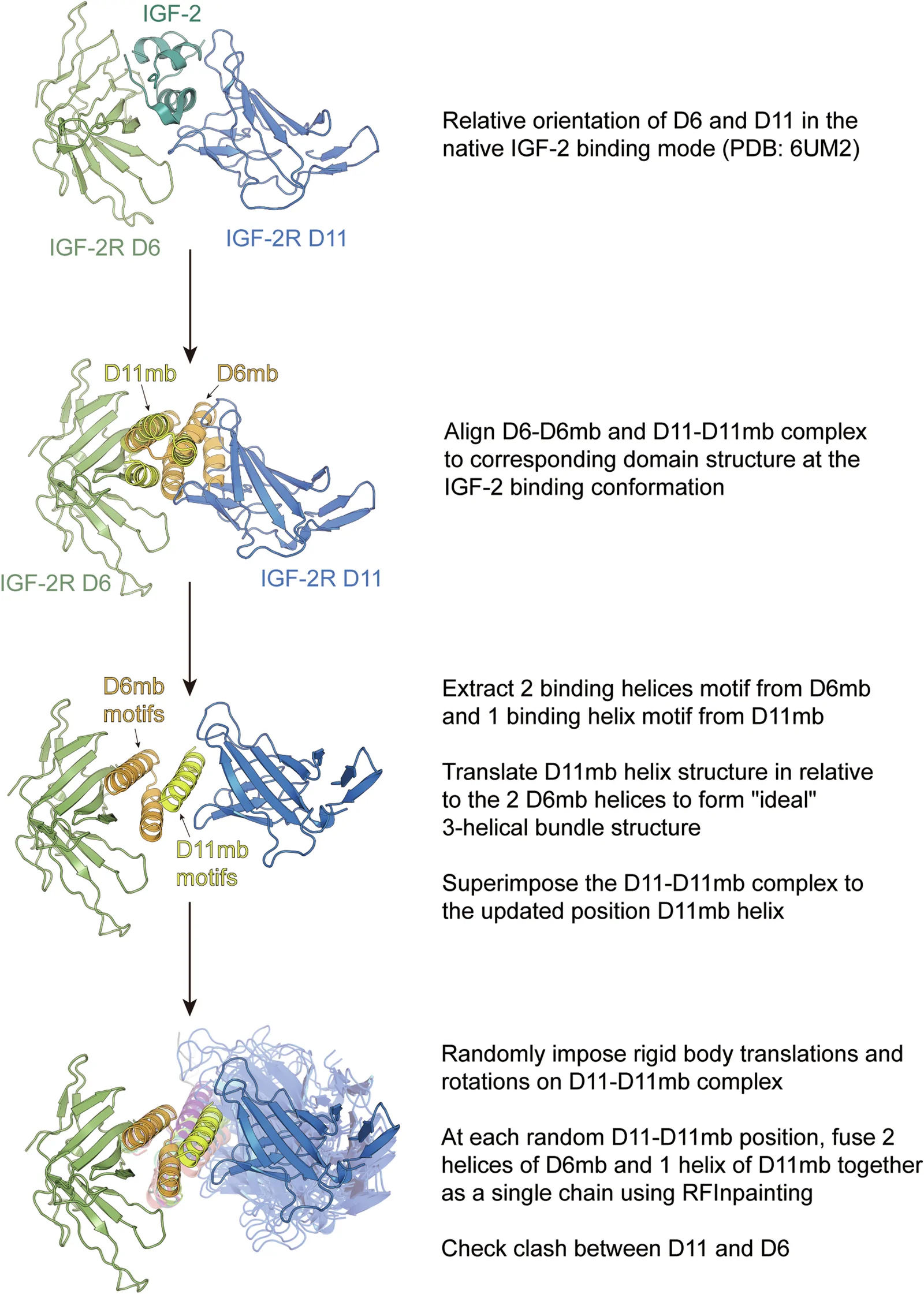
Computational design strategy to make rigid IGF_EndoTags (EndoTag3 and EndoTag4). Starting from the structure of IGF-2 in complex with IGF2R, de novo minibinders were generated and screened against the IGF-2 binding sites at IGF2R domain 6 and domain 11, separately. Individual binders for each domain (D6mb and D11mb) were fused with flexible linkers or a rigid fusion interdomain connection. For flexible fusion, multiple linker lengths and fusion directions were sampled. For rigid fusion, the two major binding helices from D6mb and one major binding helix from D11mb were extracted as starting motifs. With protein Inpainting, geometries and fusion orders were sampled, and ranked based on Rosetta and alphafold2 metrics.

**Extended Data Fig. 4:**
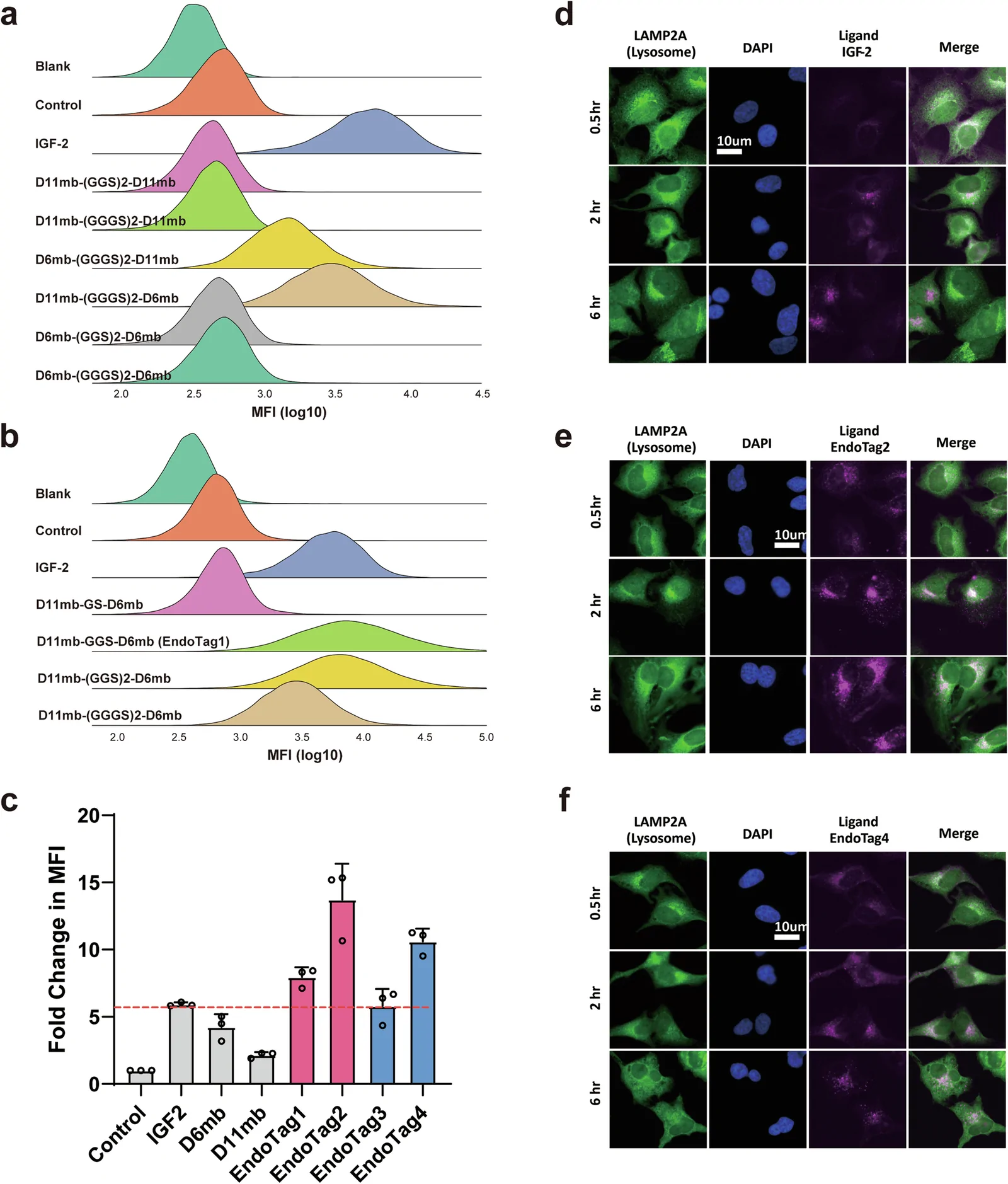
Cellular uptake evaluation IGF_EndoTags. **a**, Cellular uptake comparison between homodimer and heterodimer fusion of D6mb and D11mb. **b**, Cellular uptake comparison of linker length of D11mb-D6mb fusion with various lengths of GS linkers in the middle. **c**, Cellular uptake of IGF_EndoTags. Jurkat cells were treated with biotinylated 100 nM IGF_EndoTags or IGF-2, and 33 nM Strapavidin-AF647 for 24 h. After washing 3 times, the cellular uptake was measured by flow cytometry. The data were normalized with the control group treated with 33 nM Strapavidin-AF647 alone. The data was collected as mean values ± SEM across n = 3 biologically independent samples. **d**, Fluorescence Microscopy imaging of IGF-2 co-localized with lysosomal. **e**, Fluorescence Microscopy imaging of EndoTag2 co-localized with lysosomal. **f**, Fluorescence Microscopy imaging of EndoTag4 co-localized with lysosomal. For **a**, and **b**, 200 nM biotinylated fusion proteins were incubated with 50 nM of Strapavidin-AF647 and incubated with Jurkat cells for 24 h. After wash 3 times with cold PBS, the cellular uptake was measured by flow cytometry. For **d**-**f**, HeLa cells were incubated with 100 nM of biotinylated IGF-2, EndoTag2 or EndoTag4 for various time length. After cells were washed twice and fixed, they were stained with anti-LAMP2A antibody followed by goat secondary anti-IgG Alexa Fluo 488 antibody and DAPI. Epifluorescence imaging was conducted on a Yokogawa CSU-X1 microscope. These images are representative of three independently replicated samples per time point.

We reasoned that more potent stimulation of endocytosis could be achieved by using two domain constructs in which the individual domains are rigidly fused to each other to drive specific conformational changes in receptors. We aligned the major interface helix of D11mb and the two interface helices of D6mb (Fig. 1e and Extended Data Fig. 3) on the basis of their binding modes to IGF2R, sampled the rigid-body orientation between the domain 11- and domain 6-binding elements to explore a range of induced receptor conformations, and connected the two chains were using RFInpainting^24^. The sequence of the fusions was designed in the context of IGF2R using ProteinMPNN^21^, keeping residues that are in contact with the receptor constant. We tested 170 designs for which AlphaFold2^25^ predictions matched the intended structures for binding to both IGF2R domains, and expressed eight designs that interacted with both domains in *E. coli*. Different designs had distinct affinities for domain 6 and domain 11; for example, IGF_EndoTag3 possessed strong binding affinity to both domains (6 nM for domain 6 and 190 nM for domain 11; Extended Data Fig. 2d and Extended Data Table 1), whereas EndoTag4 bound more tightly to domain 6 (15 nM for domain 6 and 4.3 µM for domain 11; Extended Data Fig. 2e). In cellular uptake assays, IGF_EndoTag3 was internalized similarly to IGF2 and twofold more efficiently than IGF_EndoTag4 (Extended Data Fig. 4c), and both designs co-localized with lysosomes within 30 min (Extended Data Fig. 4f).

The M6P-binding site on IGF2R is largely occupied by M6P-tagged lysosomal hydrolases, limiting the maximal degradation capacity through this receptor, and knockout of the M6P biosynthesis enzyme GNPTAB increased binding of M6P-conjugated proteins to the cell surface^8^. The IGF_EndoTags were designed to bind to an orthogonal binding site to M6P, thus competition with M6P-tagged enzymes should not be an issue. Indeed, the extent of binding of IGF_EndoTag2 was not altered by knockout of GNPTAB in UMRC2 cells (Extended Data Fig. 7c), confirming that competition with M6P-tagged endogenous proteins does not limit EndoTag function, an advantage over the original direct M6P conjugation strategy. Although the binding site of IGF_EndoTag4 is proximal to the IGF2-binding site on IGF2R domain 11, pre-incubation of cells with IGF_EndoTag4 did not inhibit IGF2 uptake or transport, whereas IGF_EndoTag2 did reduce IGF2 uptake (Extended Data Fig. 7a,b), indicating that orthogonality with IGF2 interaction can be achieved by modulating the binding affinity to IGF2R domain 11 while preserving the binding to domain 6.

## Clustering receptors

The endocytosis of receptors such as ASGPR and EGFR is stimulated through dimerization or oligomerization^26^. ASGPR is a liver-specific receptor that transports *N*-acetylgalactosamine (GalNAc)-labelled proteins into lysosomes for clearance^27^. Multivalent GalNAc ligands have been used for multiple liver-specific degradation applications^2,28^ and RNA delivery platforms^29^. However, these require chemical modification and thus are not genetically encodable and must compete with native ligands.

We designed binders to ASGPR that do not overlap with the glycan-binding sites (Extended Data Fig. 1h,i) using an updated version of the Rosetta design approach described above that uses ProteinMPNN^21^ for sequence design and AlphaFold2^25^ for design evaluation. We used yeast display for 2,689 designs that were predicted to bind ASGPR by AlphaFold2^25^ followed by fluorescence-activated cell sorting (FACS) and next-generation sequencing to reveal four highly enriched designs. These were expressed in *E. coli*, and following purification, BLI showed that the design with the highest affinity, ASmb1, bound to ASGPR with an affinity of 2.7 µM (Extended Data Fig. 2f and Extended Data Table 1).

To stimulate ASGPR endocytosis through clustering, we connected two or three ASmb1 domains with GS linkers to generate ASGPR EndoTags, which we refer to as AS_EndoTag-2C and AS_EndoTag-3C (Fig. 1h). Following a 2 h incubation with Hep3B cells and extensive washing, 2.5-fold and 4.5-fold more fluorescence was associated with cells for AS_EndoTag-2C and AS_EndoTag-3C, respectively, compared with monomeric ASmb1 (Fig. 1i). Confocal imaging showed that AS_EndoTag-3C strongly co-localized with lysosomes after 24 h (Fig. 1j and Supplementary Fig. 18c). To determine the oligomerization state induced by AS_EndoTag, we mixed ASGPR and AS_EndoTag-3C at a 1:3 ratio and separated the generated species by size-exclusion chromatography (SEC); AS_EndoTag induced formation of a trimeric ASGPR complex as expected given the three ASGPR-binding sites (Supplementary Fig. 23).

## Cell surface receptor degradation

**Fig. 2:**
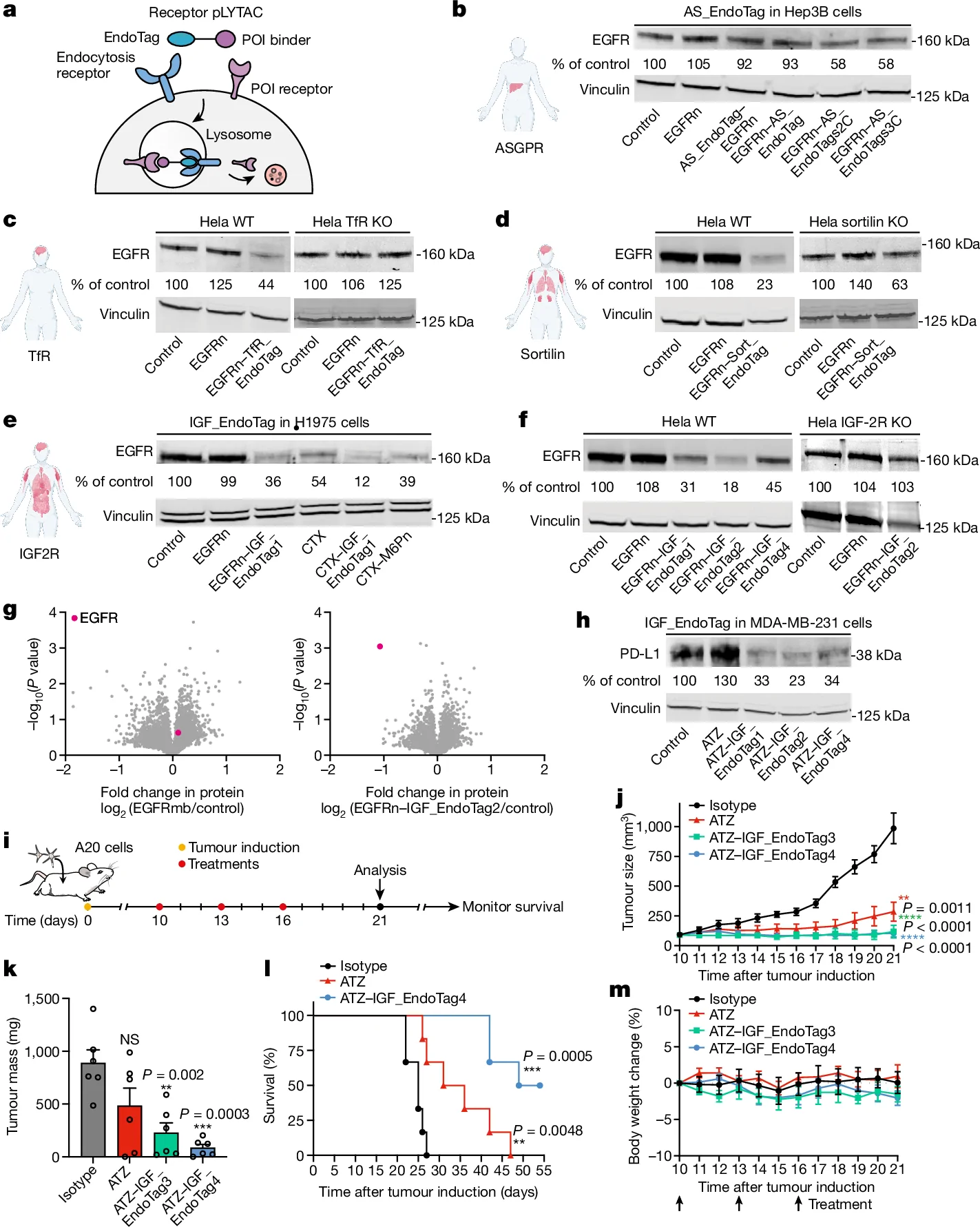
Surface receptor degradation with tissue-specific pLYTACs. **a**, Schema of tissue-specific pLYTACs for receptor degradation. POI, protein of interest. **b**, Western blot analysis of total EGFR in Hep3B cells after treatment with 200 nM EGFRn or EGFRn–AS_EndoTag for 48 h. **c**, Western blot analysis of total EGFR levels in wild-type (WT) or TfR-knockout (KO) HeLa cells after treatment with 200 nM EGFRn or EGFRn–TfR_EndoTag for 48 h. **d**, Western blot analysis of total EGFR in wild-type or sortilin-knockout HeLa cells after treatment with 200 nM EGFRn or EGFRn–Sort_EndoTag for 48 h. **e**, Western blot analysis of total EGFR in H1975 cells after treatment with 200 nM of EGFRn or CTX with or without fusion to EndoTag1 or M6Pn for 48 h. **f**, Western blot analysis of total EGFR in wild-type or IGF2R-knockout HeLa cells after treatment with 200 nM EGFRn or EGFRn–IGF_EndoTags for 48 h. **g**, Quantitative proteomics analysis of protein abundance in H1975 cells. Data are mean of three biological replicates. Two-tailed unpaired *t*-test with Welch’s correction. EGFRmb, EGFR minibinder. **h**, Western blot analysis of PD-L1 in MDA-MB-231 cells after treatment with 200 nM ATZ or ATZ–pLYTACs for 48 h. **i**–**m**, Schema (**i**) of in vivo study. Five million A20 tumour cells were inoculated subcutaneously into BALB/c mice on day 0, then 5 mg kg^−1^ of indicated reagent (*n* = 6) was administered intratumourally on days 10, 13 and 16 (**j**–**m**). **j**, Tumour growth curve over time. One-way ANOVA. **k**, Tumour mass measured at day 21. **l**, Overall survival of treated mice. *P* value by log-rank (Mantel–Cox) test. **m**, Body weight of treated mice. One-way ANOVA. In **j**–**l**, data are mean ± s.e.m. of *n* = 6 biological independent samples. NS, not significant. Source Data

LYTACs utilize mannose-6-phosphonate (M6Pn) ligands that trigger lysosomal delivery and degradation of the targeted proteins through the IGF2R^1,30^, or GalNAc to trigger the ASGPR lysosomal trafficking pathway^2^. Although promising, the LYTAC approach is hindered by the reliance on existing native ligands^8^ and by the sophisticated chemistry required to generate multivalent modifications that increase endocytosis potency, complicating their manufacturing. Given their potent and rapid endocytosis and lysosome-targeting ability, we hypothesized that the fusion of EndoTags with target-specific binders to generate protein–LYTACs (pLYTAC) (Fig. 2a) could provide an orthogonal and genetically encoded approach for efficient extracellular protein degradation, and the different tissue distributions of the different receptors could enable targeting of degradation to distinct tissues.

We began by investigating the ability of EndoTags to target and degrade EGFR, which is frequently overexpressed in cancers and has an important role in regulating cell proliferation^31^. We first assessed the degradation efficiency of the liver-specific AS_EndoTags. Consistent with the cellular uptake results (Fig. 1i), introduction of fusions of AS_EndoTags-2C of AS_EndoTags-3C with a minibinder targeting the N terminus of EGFR (EGFRn) resulted in a 40% decrease in total EGFR levels, whereas fusions to the monomeric ASGPR binder had little effect (Fig. 2b). Thus, the ASGPR EndoTags function as liver-specific targeted degraders. To generate EGFR–pLYTACs targeting the brain, we fused TfR_EndoTag or Sort_EndoTag with EGFRn^15^. We observed efficient clearance of EGFR in wild-type HeLa cells after 48 h incubation, with EGFRn-TfR_EndoTag resulting in a 55% reduction of EGFR (Fig. 2c) and EGFRn–Sort_EndoTag resulting in a 78% reduction of EGFR (Fig. 2d).

To confirm that the EndoTags function through their target receptor, we carried out parallel experiments in sortilin-knockout or TfR-knockout HeLa cells (Supplementary Fig. 22). Knockout of these receptors eliminated EGFR degradation by the corresponding EndoTags, demonstrating that degradation is dependent on the target receptors (Fig. 2c,d). As a further test, we introduced mutations into Sort_EndoTag to eliminate sortilin binding based on the design model and mutation scanning data (EGFRn–Sort_EndoTagKO). The EGFR-degradation capacity of EGFRn–Sort_EndoTagKO was largely ablated compared with EGFRn–Sort_EndoTag, further confirming that EGFR degradation requires sortilin engagement (Supplementary Fig. 20). Given the abundant expression of the corresponding receptors in the brain, both TfR_EndoTag and Sort_EndoTag could function as pLYTACs for applications in neurodegenerative disease.

**Extended Data Fig. 5:**
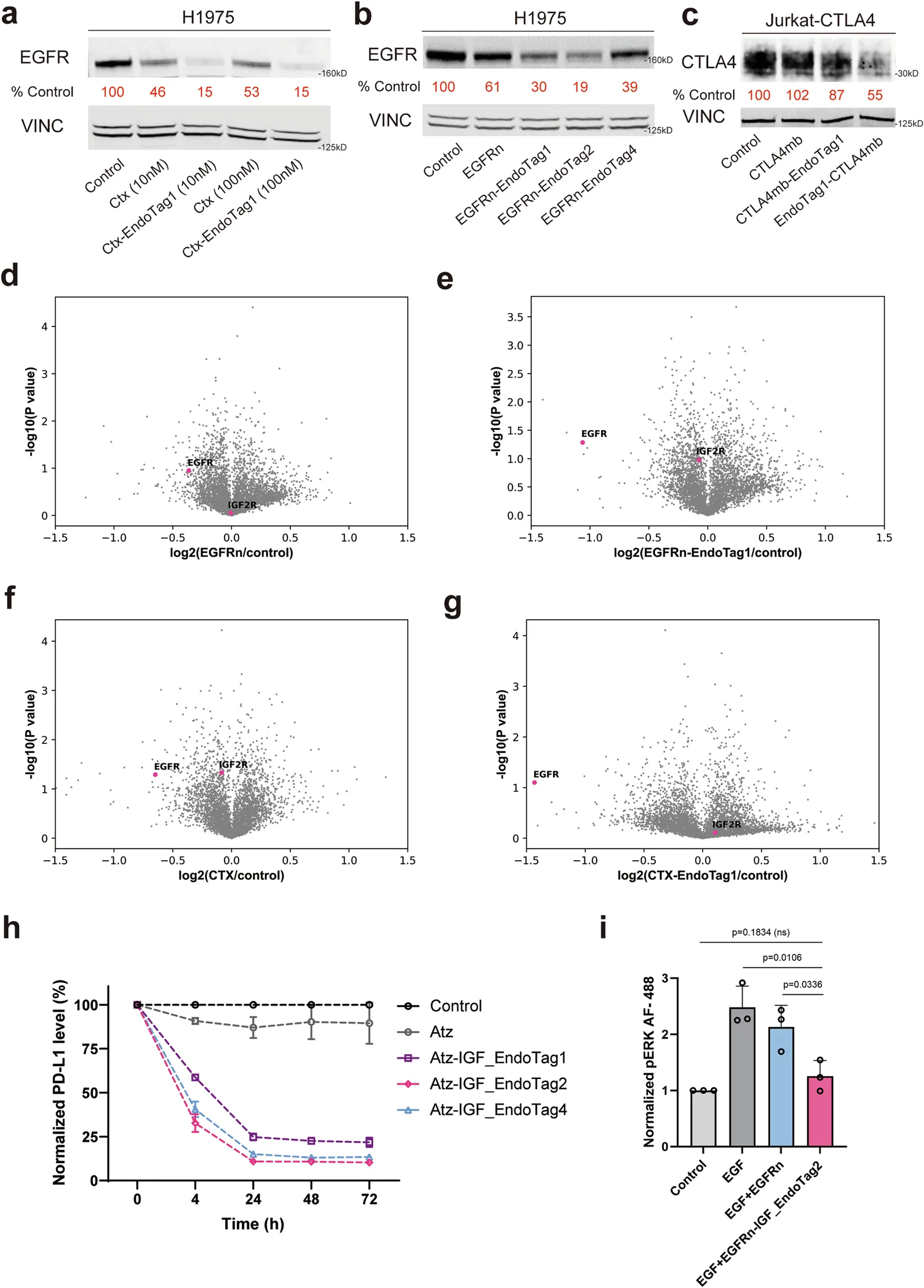
Receptor degradation with EndoTags. **a**, Levels of EGFR after treatment with 10 nM or 100 nM CTX-M6P or CTX-IGF_EndoTag1 in H1975 cells for 48 h. **b**, Levels of EGFR after treatment with 100 nM EGFRn or EGFRn-IGF_EndoTags in H1975 cells for 48 h. **c**, Levels of CTLA4 with 200 nM of CTLA4mb or CTLA4mb-IGF_EndoTag1 in Jurkat-CTLA4 cells after treatment for 3 h. **d**, Fold change in abundance of EGFR with treatment of EGFRn compared with control (untreated group). **e**, Fold change in abundance of EGFR with treatment of EGFRn-IGF_EndoTag1 compared with control (untreated group). **f**, Fold change in abundance of EGFR with treatment of CTX compared with control (untreated group). **g**, Fold change in abundance of EGFR with treatment of CTX-IGF_EndoTag1 compared with control (untreated group). **h**, Flow cytometry analysis of surface PD-L1 levels in MDA-MB-231 cells after treatment with 200 nM ATZ or ATZ-pLYTACs. MFI was normalized by the PD-L1 level of untreated groups. The data was collected as mean values ± SEM across n = 3 biologically independent samples. **i**, **Functional EGF signaling assay**. Hela WT cells were pre-treated with 100 nM of EGFRn, EGFRn-IGF_EndoTag2 or PBS control for 24 h, and then washed and stimulated with 100 nM of human EGF for 15 min followed by phosphorylation flow cytometry using anti-pERK AF-488. Data represents mean of biological triplicates and error bar indicates standard deviation. P values were determined by unpaired two-tailed t-test. For **d**-**g**, the proteomic data was collected in H1975 cells with the treatment 100 nM of corresponding reagents for 48 h and data collected is the replicated with sample size n = 2. P values were calculated by a two-tailed unpaired t-test with Welch’s correction.

We next sought to make systemically active pLYTACs that act through the ubiquitously expressed IGF2R. Addition of IGF_EndoTag–EGFRn fusions to H1975 or HeLa cells reduced EGFR levels (Fig. 2e,f, Extended Data Fig. 5b and Supplementary Fig. 21); EGFRn–IGF_EndoTag2 was the most effective opf these fusions, leading to more than 80% clearance of EGFR. Mass spectrometry-based proteomic analyses showed that EGFRn–IGF_EndoTag2 and EGFRn–IGF_EndoTag1 reduced EGFR levels in HeLa and H1975 cells without affecting IGF2R levels (Fig. 2g, Extended Data Fig. 5e and Supplementary Table 5); EGFRn without EndoTag had no effect on EGFR levels (Fig. 2g and Extended Data Fig. 5d). The EGFR reduction induced by EGFRn–IGF_EndoTag2 in HeLa cells was eliminated by IGF2R knockout (Fig. 2f), further confirming the receptor dependence of the degradation mechanism. Consistent with this, mutations in IGF_EndoTag2 predicted to eliminate IGF2R binding largely ablated the EGFR degradation-inducing activity (Supplementary Fig. 20). To investigate the functional consequences of EGFR knockdown, HeLa cells pre-treated with 100 nM EGFRn–IGF_EndoTag or EGFRn control for 24 h were stimulated with EGF and downstream phospho-ERK signalling was detected by phosphorylation flow cytometry. Compared with EGFRn control, pre-incubation of EGFRn–IGF_EndoTag largely ablated EGF signalling (Extended Data Fig. 5i).

To compare these systemically active pLYTACs with the original M6P-based LYTACs, we generated genetic fusions of EndoTag1 with cetuximab (CTX), a clinically approved therapeutic antibody that targets EGFR with high affinity^32^. In H1975 cells, CTX–IGF_EndoTag1 led to more effective degradation of EGFR than the M6P-based LYTAC^1^ (Fig. 2e). Proteomic analyses demonstrated that CTX–IGF_EndoTag1 elicited a significantly greater reduction in EGFR levels than CTX alone (Extended Data Fig. 5f,g), with little effect on the amount of IGF2R; incubation with 10 nM CTX–IGF_EndoTag1 led to an 85% reduction in EGFR (Extended Data Fig. 5a).

We next investigated targeted degradation of PD-L1, an immune checkpoint used in cancer immunotherapy^33^. We genetically fused pLYTACs to the C terminus of the PD-L1 antibody atezolizumab^34–36^ (ATZ) and tested the ability of this construct to clear PD-L1. The ATZ–EndoTag fusions reduced the amount of PD-L1 in MDA-MB-231 cells within 4 h (Extended Data Fig. 5h), and 77% of the PD-L1 in the cells was eliminated after 48 h (Fig. 2h). Similar to PD-L1, CTLA4 is an immune checkpoint component for which inhibitors have shown promising anti-tumour effects^18,19^. A fusion of EndoTag1 and a minibinder against CTLA4^37^ (CTLA4mb) resulted in a 45% decrease of CTLA4 in Jurkat cells expressing CTLA4 (Jurkat-CTLA4) cells after 3 h (Extended Data Fig. 5c).

To evaluate EndoTag function in vivo, we compared the efficacy of the ATZ–EndoTag fusions described above in a mouse tumour model compared with ATZ alone. BALB/c mice were inoculated subcutaneously with A20 cells to initiate tumour growth, and were treated intratumourally with EndoTag constructs when the tumour size reached around 100 mm^3^. The proteins were administered every 3 days for 9 days at 5 mg kg^−1^ per injection. When the tumour volume in the isotype control group reached around 1,000 mm^3^, the mice were euthanized and the tumours were weighed and collected for western blot analysis (Fig. 2i). ATZ–IGF_EndoTag3 and ATZ–IGF_EndoTag4 were considerably more effective than ATZ alone in reducing tumour size and mass (Fig. 2j,k). ATZ–IGF_EndoTag4 markedly increased overall survival compared with ATZ alone or isotype control: after 55 days, half of the mice treated with the EndoTag fusion remained alive, whereas all mice treated with ATZ alone had died (Fig. 2l). Western blot analysis showed that ATZ–IGF_EndoTag3 and ATZ–IGF_EndoTag4 triggered significant degradation of PD-L1 compared with ATZ alone or isotype control (Extended Data Fig. 8). Body weight assessment showed that the EndoTag treatments were well-tolerated (Fig. 2m). Thus, the efficacy of antagonistic antibodies can be enhanced by fusion to EndoTags; such fusions not only block disease-asociated interactions of the target (like the unfused antibody) but also induce cellular uptake and degradation of the target in the lysosome.

## Clearance of soluble proteins

**Fig. 3:**
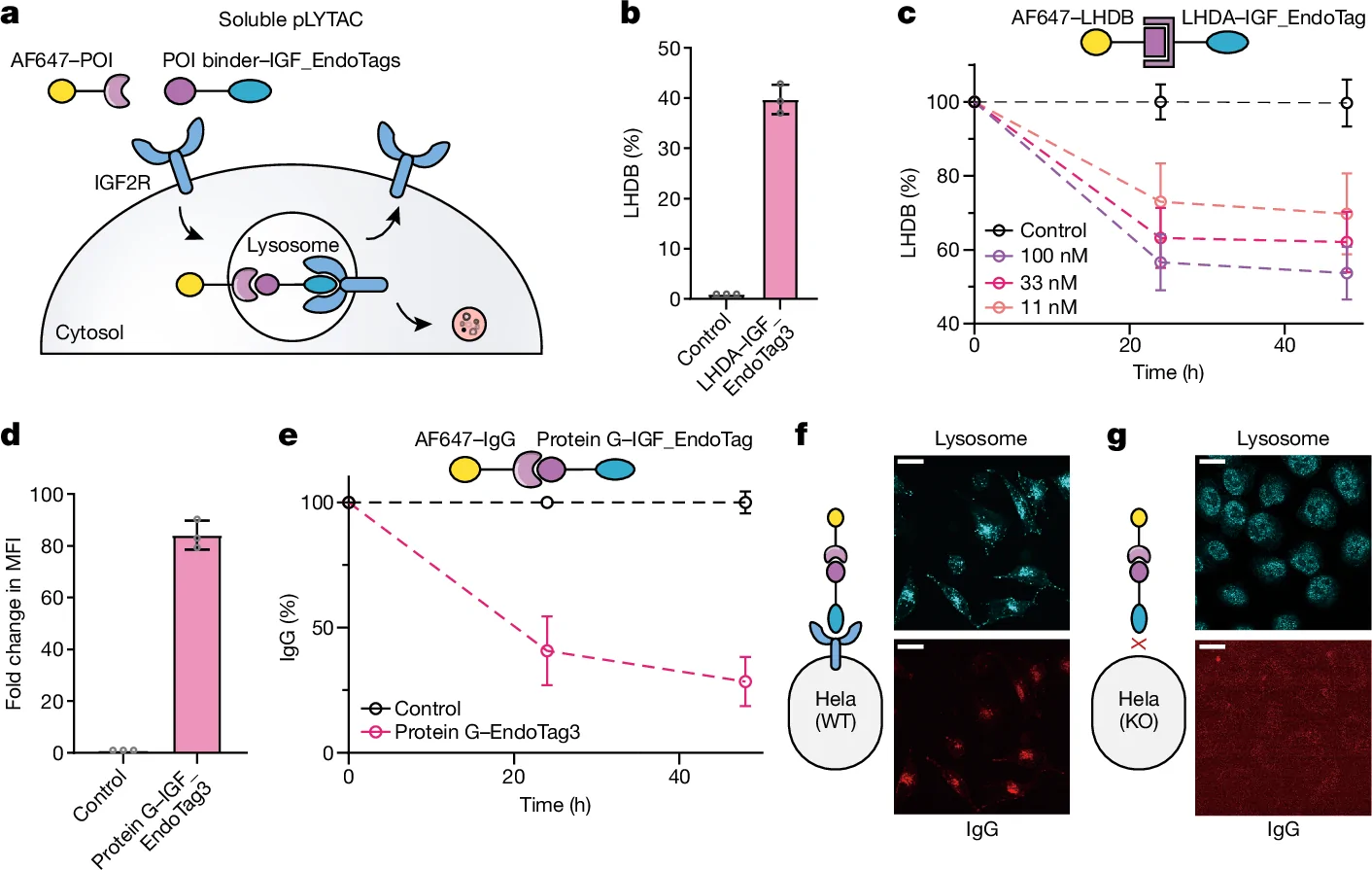
Clearance of soluble proteins by IGF2R pLYTACs. **a**, Schema for the use of soluble pLYTACs with IGF_EndoTags. **b**, Cellular uptake of LHDB–AF647 via LHDA–IGF_EndoTags in Jurkat cells. Cells were incubated with 33 nM LHDB–AF647 with or without 1 μM LHDA–IGF_EndoTags for 24 h, washed twice with cold PBS and analysed by flow cytometry. **c**, Remaining supernatant LHDB–AF647 levels in Jurkat cells. Jurkat cells were incubated with 100 nM LHDB–AF647 with or without 500 nM LHDA–IGF_EndoTags. At timepoints 24 h and 48 h, the cells were pelleted down, and IgG in the supernatant was quantified using a Neo2 plate reader. IgG level was normalized to the IgG-alone control group. **d**, Cellular uptake of IgG–AF647 via protein G–IGF_EndoTags in K562 cells. Cells were incubated with 33 nM IgG–AF647 with or without 1 μM protein G–IGF_EndoTag3 for 24 h, washed twice with cold PBS and analysed by flow cytometry. The fold change in MFI was calculated by normalizing to the IgG–AF647-alone group. **e**, Remaining IgG–AF647 levels in the supernatant of Jurkat cells. Jurkat cells were incubated with 133 nM IgG–AF647 with or without 100 nM protein G–IGF_EndoTag3. At timepoints 24 h and 48 h, the cells were pelleted down, and IgG–AF647 in the supernatant was quantified using a Neo2 plate reader. The IgG–AF647 level was normalized to the IgG–AF647-alone control group at each timepoint. **f**, Confocal imaging of IgG–AF647 co-localization with lysosome in HeLa cells. **g**, Confocal imaging of IgG–AF647 co-localization with lysosome in HeLa (IGF2R-knockout) cells. Representative images of three replicated samples. **f,g**, Cells were incubated with 200 nM IgG–AF647 and 1 μM protein G–IGF_EndoTag3 for 24 h, washed and stained with LAMP2A antibody followed by AF488-labelled secondary antibody. Scale bars, 20 µm. Data in **b**–**e** are mean ± s.e.m. of *n* = 3 biologically independent samples.

**Extended Data Fig. 6:**
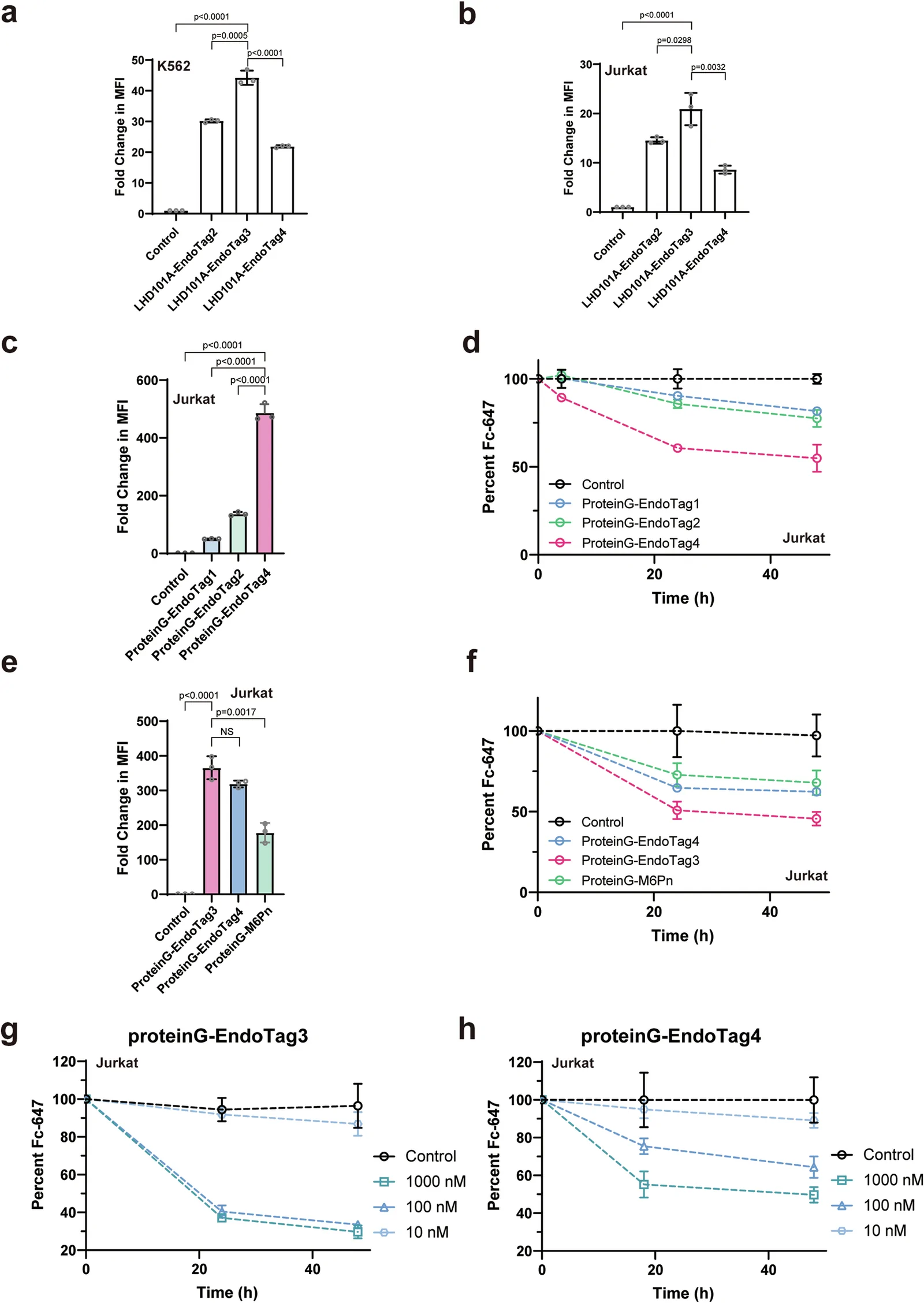
Clearance of soluble proteins with EndoTags. **a**, LHDB-AF647 cellular uptake ability comparison among flexible and rigid LHDA-IGF_EndoTags in K562 cells. **b**, LHDB-AF647 cellular uptake ability comparison among flexible and rigid LHDA-IGF_EndoTags in Jurkat cells. **c**, IgG-AF647 cellular uptake ability comparison between flexible and rigid designs. **d**, Quantitative clearance of IgG-AF647 in cell media comparison between flexible and rigid designs. **e**, IgG-AF647 cellular uptake ability comparison between pLYTACs and M6Pn. **f**, Quantitative clearance of IgG-AF647 in cell media comparison between pLYTACs and M6Pn. **g**, Quantitative clearance of IgG-AF647 in cell media with titrated proteinG-EndoTag3. **h**, Quantitative clearance of IgG-AF647 in cell media with titrated proteinG-EndoTag4. For **a**,**b**, cells were incubated with 100 nM LHDB-biotin + 33 nM Strapavidin-AF647 with/without 500 nM LHDB-pLYTACs for 48 h, washed twice with cold PBS and analyzed by flow cytometry. The fold change in MFI (mean fluorescence intensity) was calculated by normalizing the LHDB-AF647 alone group. For **c**,**e**, cells were incubated with 33 nM IgG-AF647 with/wihtout 1uM proteinG-IGF_EndoTags for 24 h, washed twice with cold PBS and analyzed by flow cytometry. The fold change in MFI (mean fluorescence intensity) was calculated by normalizing the IgG-AF647 alone group. For **a**,**b**,**c**,**e**, data are presented as mean values ± SEM with biologically replicates with n = 3. For **d**,**f**,**g**,**h**, cells were incubated with 33 nM IgG-AF647 with/without proteinG-IGF_EndoTags. At timepoints 24 h, 48 h, the cells were pelleted down, and supernatant IgG-AF647 levels were quantified by Neo2 plate reader. The percentage of IgG-AF647 level was normalized with the IgG-AF647 alone control group. For **d**,**f**,**g**,**h**, data are presented as mean values ± SEM with biologically replicates with n = 3. For **a**-**c**,**e**, p values were determined by unpaired two-tailed t-test.

**Extended Data Fig. 7:**
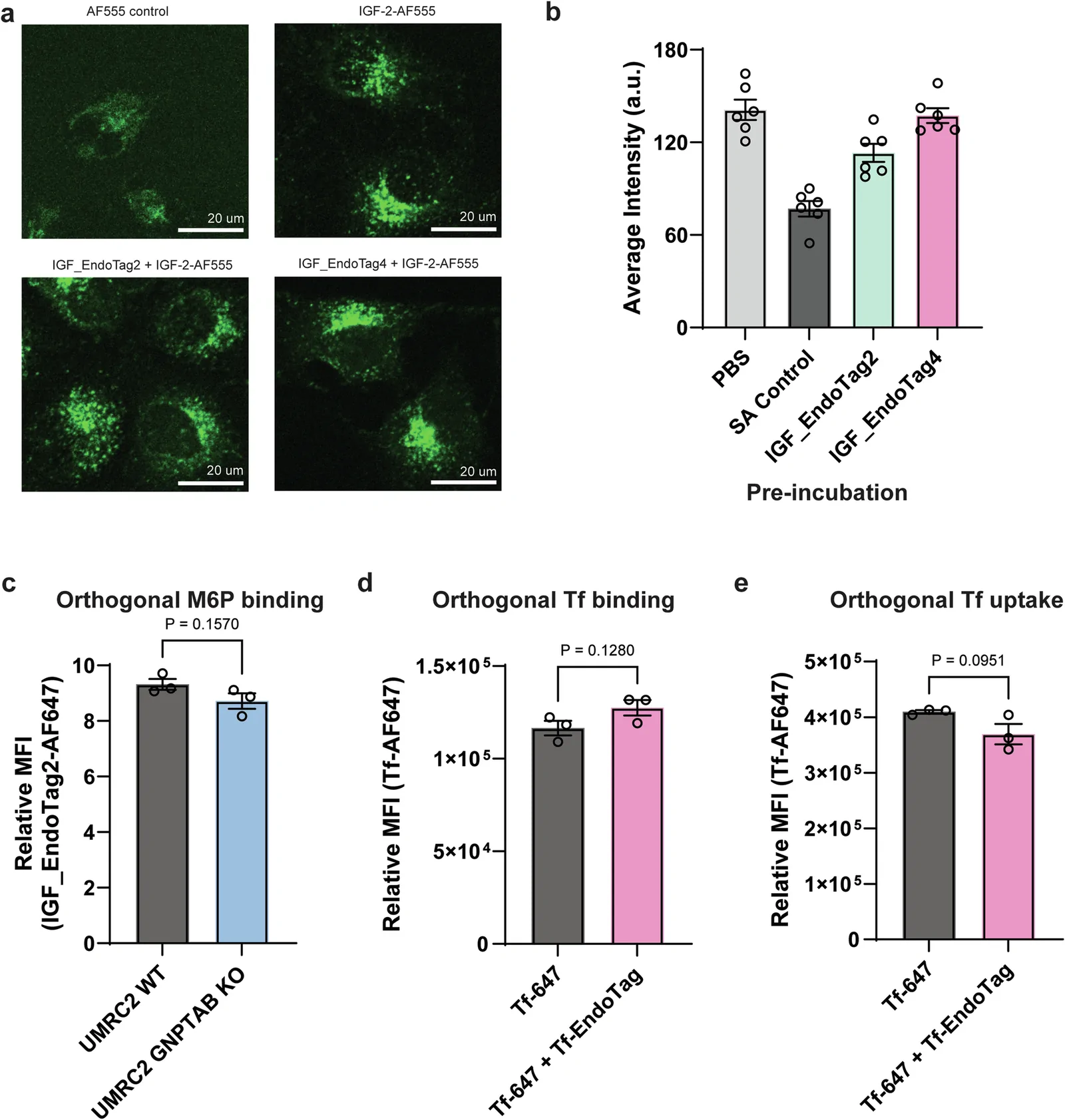
Orthogonality of EndoTag. **a**, Confocal imaging of biotinylated IGF-2 labeled with AF-555-Streptavidin (green). Hela cells were pre-incubated with 100 nM non-labelled IGF_EndoTags, PBS control for 24 h. After washing with PBS cells were incubated with AF-555 labeled Streptavidin together with biotinylated IGF-2 (the mix was preincubated for 10 min) for 4 h. Cells were washed and lysosomes were stained with AF488-labeled Lysotracker for 30 min. **b**, Averaged IGF-2 intensity in single cells based on a Lysotracker cell mask. Data represents mean and error bar indicates SEM (N = 6 images per condition with at least 10 cells per image). **c**, Binding of AF-647 labeled IGF_EndoTag2 on the cell surface of WT and GNPTAB KO UMRC2 cells on ice. **d**, Binding of AF647 labeled transferrin to HeLa cells with and without 100 nM of Tfr-EndoTag on ice. **e**, Internalization of transferrin-647 in HeLa cells with and without 100 nM TfR-EndoTag treatment. For **c**-**e**, data represents mean of 3 biological replicates and error bar indicates SEC. P values were determined by unpaired two-tailed t-test.

**Extended Data Fig. 8:**
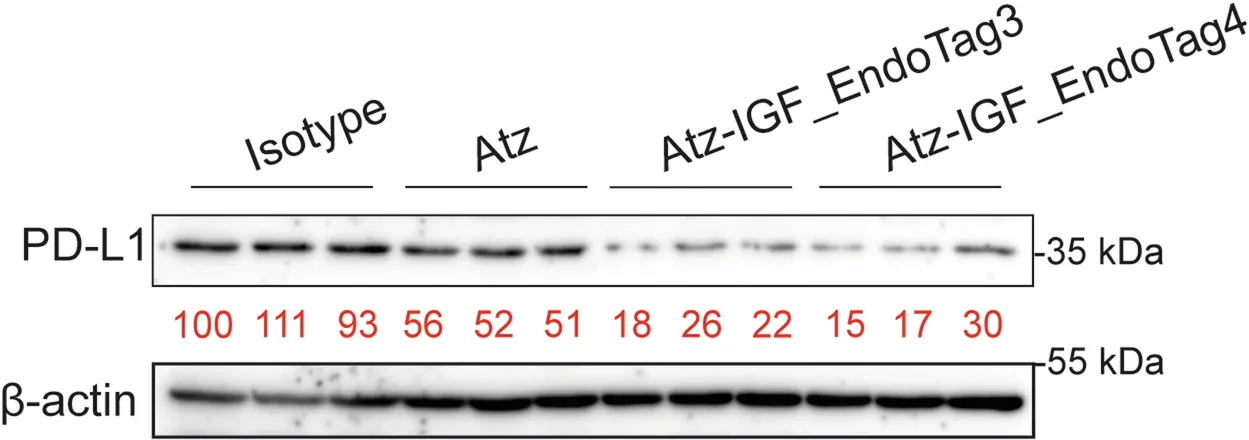
In vivo PD-L1 degradation by ATZ-pLYTAC. Western Blot analysis of PD-L1 levels in tumor tissues colleted at sacrifice at day21 from different treatment groups of mice with β-actin as loading control.

We next investigated the ability of EndoTags to degrade targeted soluble proteins (Fig. 3a). As a proof of concept, we used the nanomolar affinity de novo designed protein heterodimer pair LHD101A (LHDA) and LHD101B (LHDB) used as the basis for synthetic signalling systems^22,38^. We fused LHDA to IGF_EndoTags and LHDB to AF647, and found that the EndoTags significantly enhanced the uptake of LHDB–AF647 in Jurkat and K562 cells (Fig. 3b and Extended Data Fig. 6a,b), with IGF_EndoTag3 producing a remarkable 40-fold increase in mean fluorescence intensity compared with LHDB–AF647 alone. Incubation of Jurkat cells with 100 nM LHDA–IGF_EndoTag3 resulted in 50% clearance of 100 nM LHDB from the solution after 48 h incubation in Jurkat cells (Fig. 3c; clearance may be limited by the number of available receptors).

**Extended Data Fig. 9:**
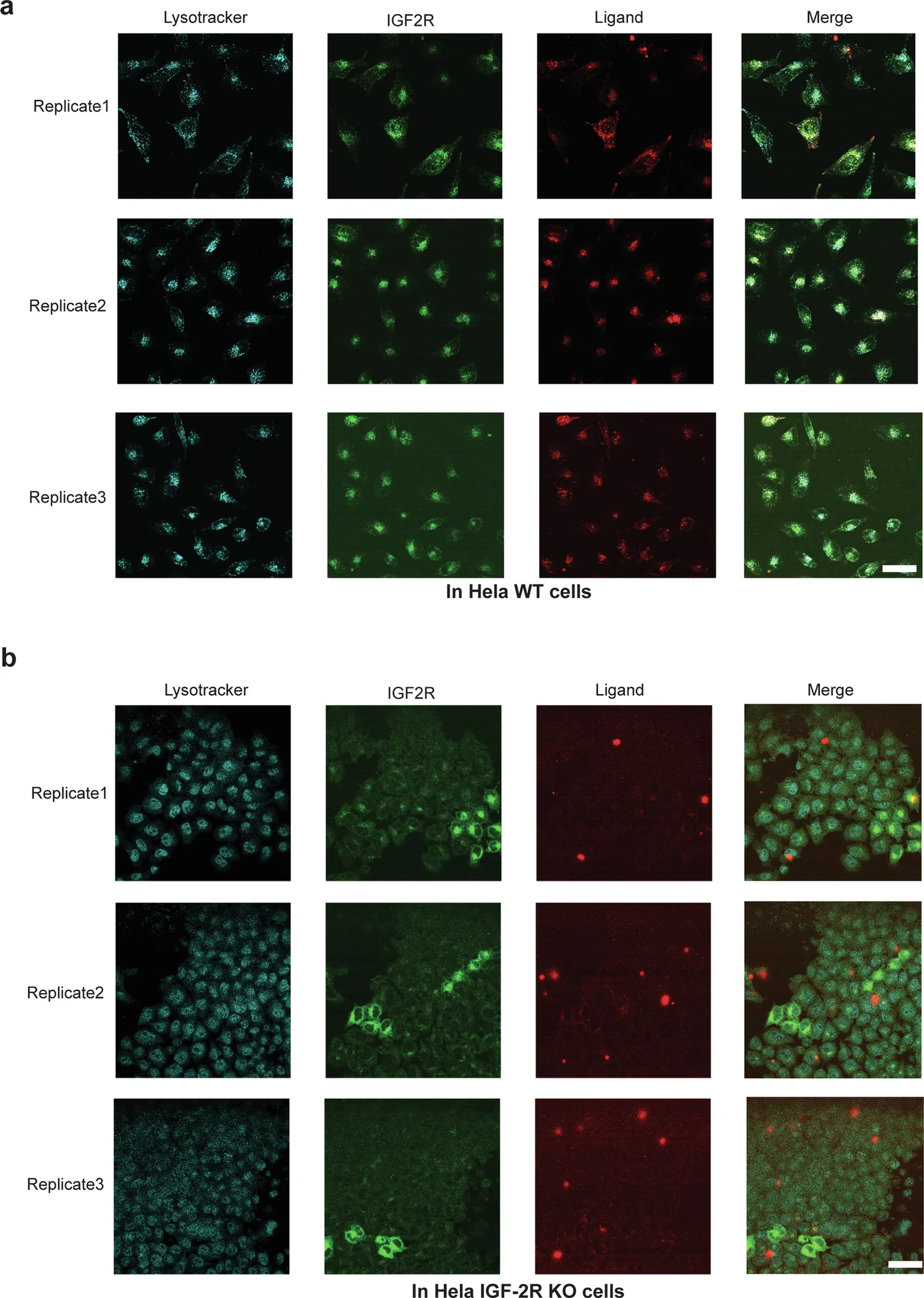
**a**, Confocal imaging of lysosome co-localization of IgG-AF647 with lysosome in Hela WT cells with ProteinG-IGF_EndoTags treatment. **b**, Confocal imaging of lysosome co-localization of IgG-AF647 with lysosome in Hela IGF2R KO cells with ProteinG-IGF_EndoTags treatment. For **a**,**b**, the cells were incubated with 200 nM IgG-AF647 and 1uM of proteinG-IGF_EndoTag for 24 h, washed and stained with anti-LAMP2A antibody followed by AF488-labelled secondary antibody. The scale bar indicates 40 µm for **a** and 50 µm for **b**.

Autoantibodies that recognize self-antigens have been linked to multiple autoimmune diseases^39^. We tested whether the fusion of EndoTags with the IgG-binding protein G^40^ could clear IgG in solution. For comparison, we used protein G–M6Pn, generated by conjugating protein G with azido-NHS ester followed by M6Pn–BCN peptide. The EndoTag- and M6Pn-coupled protein G constructs triggered substantial uptake of IgG in Jurkat and K562 cells (Extended Data Fig. 6c,e). Protein G–IGF_EndoTag3 elicited twofold higher cellular uptake of IgG than protein G–M6Pn, leading to an overall 80-fold increase in IgG in K562 cells and a 360-fold increase in Jurkat cells (Extended Data Fig. 6e) compared with protein G alone. To quantify the clearance of IgG in the solution, we measured the fluorescence intensity in the cell culture supernatant normalized to the supernatant of control cells treated with IgG–AF647 alone. Incubation of Jurkat cells with 100 nM protein G–IGF_EndoTag3 and 133 nM IgG for 48 h resulted in depletion of 70% of the IgG (Fig. 3e); less clearance was observed with the other IGF EndoTags (Extended Data Fig. 6d,f,h). Utilizing confocal microscopy of HeLa cells, we observed enhanced co-localization of IgG with lysosomes following treatment with protein G–IGF_EndoTag3 for 24 h (Fig. 3f and Extended Data Fig. 9a); similar co-localization was not observed in HeLa cells that did not express IGF2R (Fig. 3g and Extended Data Fig. 9b).

## Logic-gated and secretable degradation factors

**Fig. 4:**
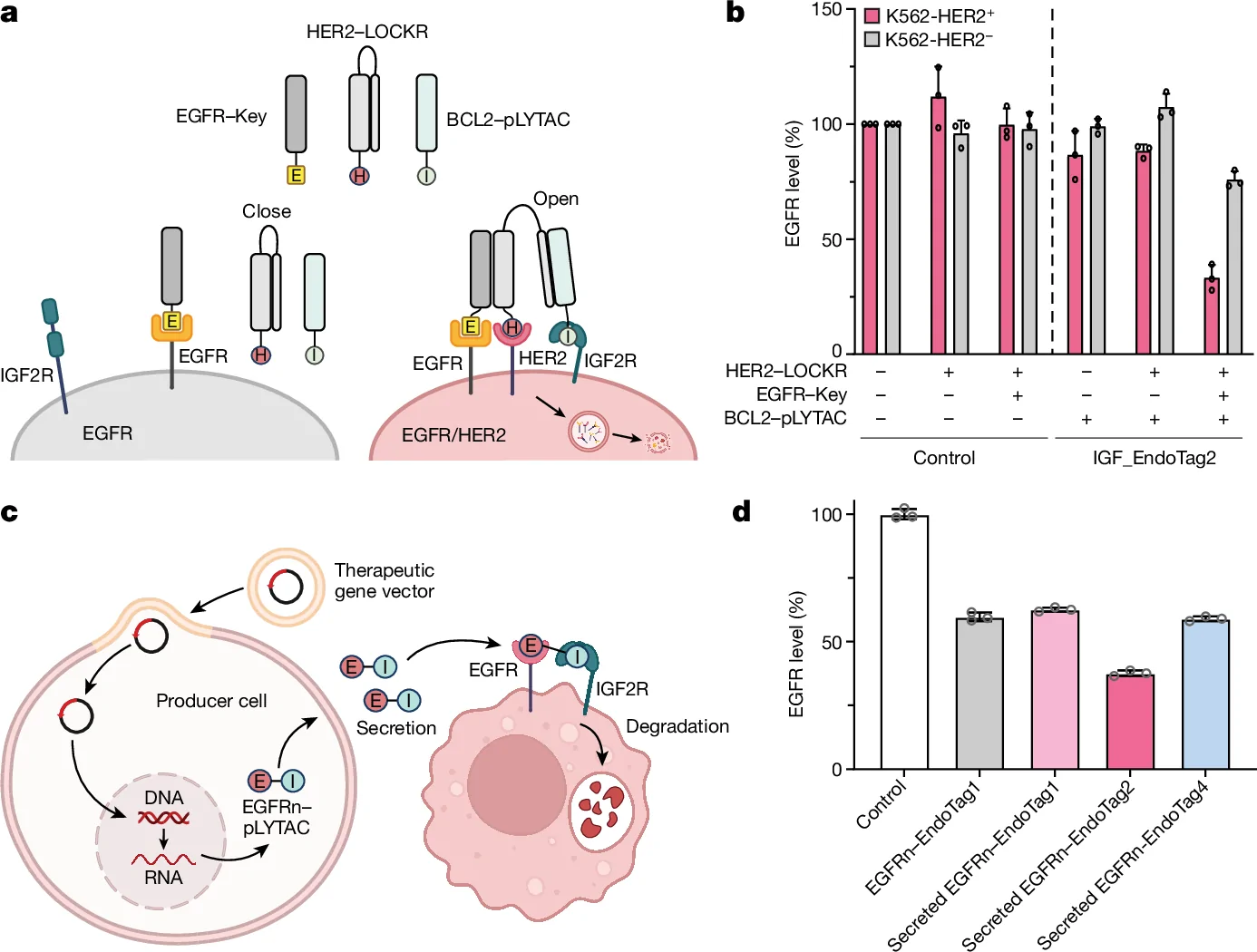
Logic-gated targeted degradation and locally secretable degraders. **a**, Schematic illustration of AND gate logic for EGFR degradation in the presence of HER2. EGFR–Key, designed fusion protein composed of an EGFR-binding domain and a ‘key’ domain; HER2–LOCKR, designed fusion protein composed of an HER2-binding domain and a ‘LOCKR’ domain with a BCL2-recognizing peptide designed to bind to LOCKR domain at weak affinity. At close proximity, the key domain will bind to LOCKR and release the BCL2-recognizing peptide. **b**, Flow cytometry quantification of EGFR on the cell surface. K562 cells overexpressing EGFR only (K562-HER2^−^) or K562 cells overexpressing both EGFR and HER2 (K562-HER2^+^) were incubated with combinations of 100 nM of EGFRn–Key, HER2–LOCKR and BCL2–EndoTag2 for 24 h. **c**, Schematic of the use of EGFR–pLYTAC secretion to degrade EGFR in target cells. **d**, Flow cytometry quantification of cell surface EGFR in cells treated with cell supernatant or exogenous EGFRn–IGF_EndoTag1 for 24 h. For the secretion groups, IGF2R-knockout HeLa cells were transfected with viral vectors encoding EGFRn–IGF_EndoTag1 or LHDA–pLYTACs. Cell supernatants were collected and incubated with K562 cells overexpressing EGFR. Data in **b**,**d** are mean ± s.e.m. of *n* = 3 biologically independent samples.

Target degradation conditioned on the presence of a specific marker in the tumour microenvironment could help to avoid undesired effects on healthy cells. Such logic-gated targeted degradation has not been achieved with current extracellular protein-degradation systems. To address this limitation, we utilized the co-localization-dependent protein switch (Co-LOCKR) system^41,42^, which functions as an AND logic gate by only exposing a recruitment motif when two target cell markers are present on the same cell (Fig. 4a). We utilized Co-LOCKR to selectively degrade EGFR only when HER2 was also present on the surface of cancer cells^41^. We fused EndoTag with BCL2, which binds the Bim peptide that is exposed upon coincident binding in this version of Co-LOCKR, and evaluated EGFR degradation in HER2^+^ and HER2^−^ cells. In K562 cells overexpressing both EGFR and HER2, addition of BCL2–IGF_EndoTag2 resulted in 80% degradation of EGFR, whereas in K562 cells expressing EGFR without HER2, the EGFR level remained unchanged (Fig. 4b). Thus, the EndoTag system can be precisely targeted to specific cells based on combinations of surface markers.

Local secretion of an EndoTag fusion introduced via mRNA delivery or as part of an adoptive cell therapy could focus degradation activity where needed and overcome depletion from lysosomal targeting. Unlike the M6P-based LYTAC system, EndoTags can be deployed in this way, as they are fully protein-based and consequently can be secreted locally with high specificity and efficiency^43,44^. To investigate this possibility, we transiently transfected IGF2R-knockout HeLa cells with plasmid encoding EGFRn–IGF_EndoTag and incubated the supernatants with EGFR^+^ K562 cells (Fig. 4c). Cell supernatants containing EGFRn–IGF_EndoTag1 and EGFRn–IGF_EndoTag2 cleared EGFR as efficiently as the purified proteins (Fig. 4d). Thus, EndoTags remain functional when secreted from cells, providing a means for adoptive cell therapies to degrade proteins in the surrounding environment for greater efficacy, and to degrade self proteins for feedback control.

## Activation of cell signalling

**Fig. 5:**
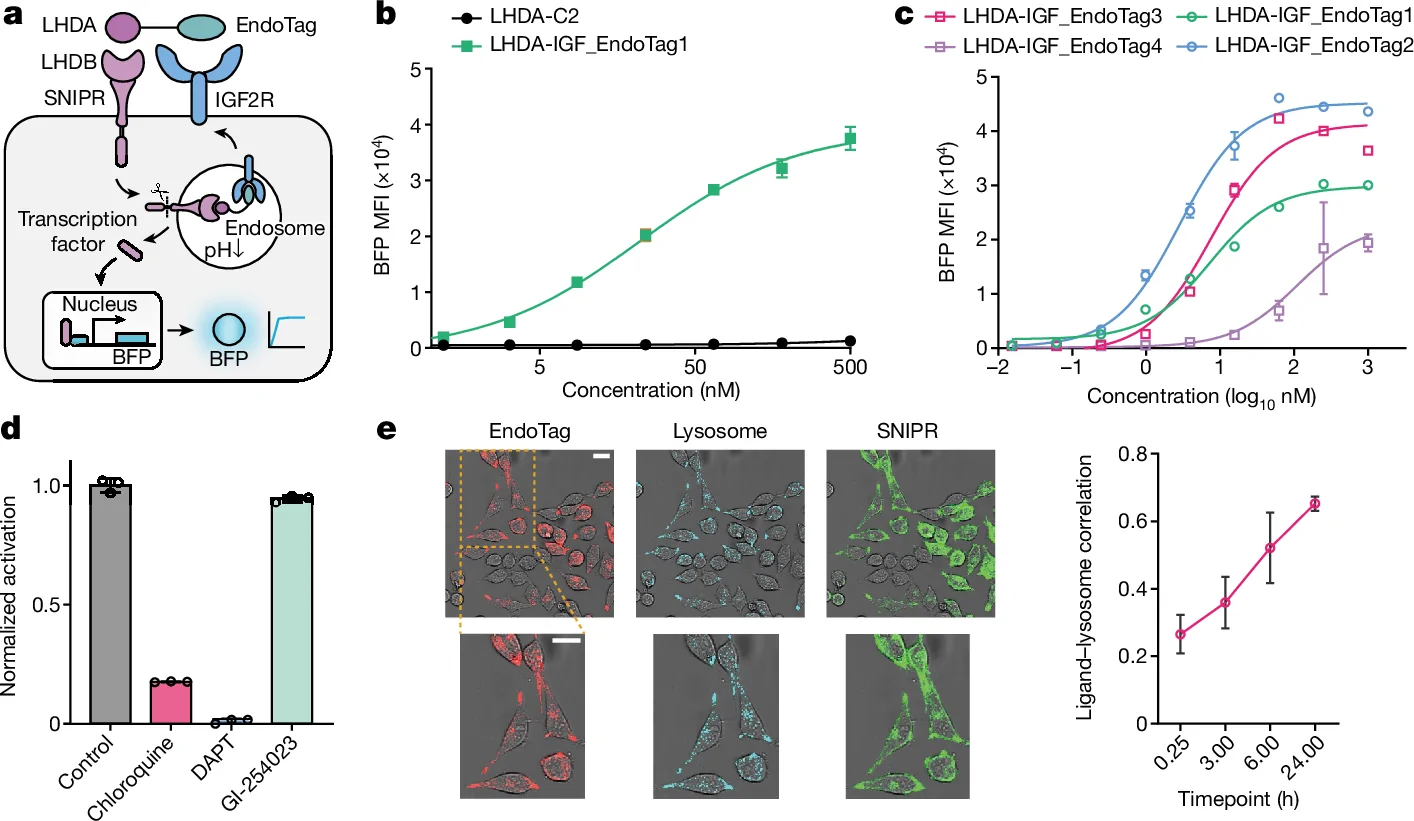
EndoTags enhance signalling. **a**, Schematic illustrating the designed SNIPR system consisting of an extracellular LHDB protein that recognizes the LHDA ligand, a cleavable membrane proximal domain and an intracellular domain that releases transcription factor, which induces expression of blue fluorescent protein (BFP). Upon ligand binding of LHDB to LHDA SNIPR, the IGF_EndoTag triggers the endocytosis of the complex; signalling activation is quantified by BFP fluorescence intensity. **b**, Activation of an LHDA-responsive SNIPR driving a BFP reporter circuit in Jurkat T cells by a non-EndoTag ligand (LHDA-C2, a homodimer composed of two LHDA molecules in C2 rotational symmetry) is much weaker than by a similarly flexibly linked EndoTag-containing ligand (IGF_EndoTag1) (*n* = 3; mean ± s.e.m.). **c**, Dose–response curve of signal activation with LHDA fusion with IGF_EndoTags. **b**,**c**, Jurkat T cells expressing LHDB SNIPR were incubated with IGF_EndoTags at the titrated concentration. *n* = 3 replicates. Data are mean ± s.e.m. **d**, Relative activation of Jurkat T cells by IGF_EndoTag1 ligand in the presence of chemical inhibitors. Data are mean ± s.e.m. of measurements normalized to the activation of an inhibitor-free vehicle control sample; *n* = 3 biologically independent samples. **e**, Left, live confocal imaging of lysosome co-localization with EndoTag ligands and SNIPR receptors at 24 h. The lysosomes were stained with AF488-labelled LysoTracker. Right, images were acquired at 0.25 h, 3 h, 6 h and 24 h for the experiment in **c** and analysed for co-localization between the labelled EndoTag ligand and LysoTracker signal. Scale bars, 20 µm for **e**. *n* = 4 images per timepoint with at least 10 cells per image; data are mean ± s.e.m.

Transmembrane signalling resulting from extracellular ligands binding to plasma membrane receptors is frequently accompanied by endocytosis of the ligand, and in some cases, signalling may take place in part or primarily in the endosome^45^. We reasoned that in such cases, EndoTags could enhance signalling by increasing the fraction of the ligand–receptor complex that is in the endosome. As a model system, we used a minimalist Notch-derived synthetic signalling system, ortho-SNIPR^38^, which inhibitor experiments suggested was primarily activated in the endosome. Ortho-SNIPR is based on de novo-designed LHDA–LHDB heterodimer pair^22^, with an LHDA-containing synthetic ligand and a receptor comprising of a LHDB extracellular domain fused to the Notch transmembrane segment; binding of the ligand to the receptor results in cleavage and release of an intracellular transcription factor domain, which activates downstream BFP expression (Fig. 5a). We found that fusion of IGF_EndoTags to LHDA resulted in an increase of up to 100-fold (in the case of IGF_EndoTag2) in signal activation (Fig. 5b,c). EndoTag-enhanced signalling was not affected by small molecules that block engagement with cell surface proteases, but was blocked by chloroquine, which disrupts endosomal acidification, suggesting that EndoTag-enhanced signalling occurs in the endosome (inhibition of γ-secretase, which carries out the proteolytic cleavage needed to free the transcription factor, also blocked signalling) (Fig. 5d). Confocal microscopy showed rapid lysosomal targeting of the LHDA–IGF_EndoTag2 construct (Fig. 5e). This marked enhancement of signalling, together with the ability to localize responses to specific target cells using tissue-specific EndoTags or Co-LOCKR targeting, should make the ortho-SNIPR system a powerful tool for synthetic biology and adoptive cell therapy applications. Further studies will be required to determine whether EndoTags can enhance signalling through endogenous pathways. Conversely, for pathways that are downregulated by endocytosis, EndoTags could be used to shorten the signalling half-life, which could have utility in applications such as T cell receptor signalling, in which overstimulation can lead to exhaustion and reduction of downstream signalling.

## Conclusion

The designed EndoTag approach considerably expands the possibilites for targeted degradation as a therapeutic modality. First, whereas native ligands can trigger off-target signalling and competition with endogenous proteins can reduce potency^8^, the designed EndoTags, as illustrated by the sortilin, TfR and ASGPR models (Fig. 1b,e,h), can be targeted to sites on endocytosing receptors that are not bound by native ligands. Second, high-valency chemical modification^1,2,46^ has been used to enhance endocytosis, but this complicates manufacturing^1,14,23^; as illustrated by the multidomain ASGPR EndoTags, small synthetic domains can be readily combined to create all protein receptor clustering and endocytosis stimulating proteins. The all-protein nature of our pLYTACs simplifies manufacturing and enables deployment of targeted degradation approaches in adoptive cell therapies using secretion from engineered cells (Fig. 4c). Although a de novo designed IL2 mimic has been shown to be not strongly immunogenic in humans^47^, as with any new therapeutic agent, it will be important to assess and, if necessary, reduce the immunogenicity of EndoTags, and catalytic versions that recycle to the plasma membrane following delivery of target to the lysosome could enable considerable dose sparing. The small, stable and readily producible EndoTags could be useful both for therapeutic applications and as molecular tools for probing how receptor conformational and oligomerization state modulates cellular trafficking.

There are many avenues for future work using our computational design approach to generating EndoTag-stimulated enhancers of cell surface receptor endocytosis and trafficking. First, there are likely to be many more receptor targets that can undergo rapid endocytosis upon suitable triggering at the cell surface; the ability to design endocytosis stimulators without requiring native ligands or identification of chemical modifications should enable utilization of the full range of these receptors to achieve more tissue-restricted targeting and modulatable intracellular trafficking (different receptors are likely to have different intracellular compartment residence times and transition dynamics). The increase in survival of mice treated with EndoTag–anti-PD-L1 fusions compared with the antibody alone (Fig. 2l) highlights the potential of EndoTag fusion to enhance the activity of antagonistic therapeutic antibodies. In addition to the targeted degradation application pursued here, such designed endocytosis stimulators could be of great utility for enhancing uptake of nucleic acids (such as short interfering RNAs) and small-molecule drug conjugates. Second, there are likely to be natural signalling pathways which, like the synthetic ortho-SNIPR system, can be more potently activated by promotion of receptor endocytosis. We achieved a 100-fold enhancement of maximum signalling effect by fusing the SNIPR ligand to EndoTags—if similar levels of signalling enhancement or modulation can be achieved by fusing natural signalling molecules to EndoTags, there could be many applications in therapeutics and biotechnology. Third, as illustrated by the use of the co-LOCKR system to make logic-gated protein-degradation systems and the secretion of EndoTag-degrader constructs from cells, the robustness and modularity of de novo designed proteins and capability for logic-gated activation and cell-based expression open the door to a wide range of more precise and controllable targeted degradation strategies for protein and cell-based therapies.

## Methods

### Computational design of sortilin minibinders as Sort_EndoTags

Using a Rosetta-based binder design protocol^15^, 21,000 binders were generated to each of 2 sites on the sortilin. The epitope of site1 comprises five amino acids (UniProt numbering: F92, V93, T546, T559 and T561), chosen since it provided a modest patch of exposed hydrophobicity while avoiding any sites of known interactions. The selected epitope has the added feature that a binder to this location would be pH-dependent, since this region undergoes considerable structural change at low pH^18^. As previously described^15^, we used a set of scaffold libraries to generate several million docks to each of the sites. As in the protocol, 100,000 docks were sub-selected and sequence was designed. Helical motifs were extracted, and 3,000 designs were selected, grafted and subjected to further design. Designs were filtered based on their Rosetta ddG and ContactMolecularSurface to the hydrophobic residues listed above. This resulted in 42,000 designs that were tested experimentally. The final designed sequences for Sort_EndoTag are provided in Supplementary Table 2.

### Computational design of IGF2R and ASGPR minibinders

The minibinders against IGF2R domain 6 and domain 11 were computationally designed via a Rosetta-based approach as described^15^. In brief, the structures of IGF2R domain 6 (Preotein Data Bank (PDB) 6UM2) and IGF2R domain 11 (PDB 1GP0) were refined using Rosetta Fastrelax with coordinate constraints. The residues at the IGF2-binding site for each domain were selected as ‘hotspot’ residues. Helical protein scaffolds were docked against the hotspot residues via the Patchdock followed by the Rifdock protocol. After sequence optimization with Rosetta FastDesign and filtering with Rosetta interface metrics including ddg and contact_molecular_surface, the top candidates were then resamplered with Rosetta Motifgraft^44^ and FastDesign. Candidates passing previous filters were then filtered again with exposed hydrophobicity (sap_score) and optimized with a net-charge of −7. The final designed sequences for IGF_EndoTag are provided in Supplementary Table 1.

The minibinders against ASGPR were designed with a Rosetta-based approach integrated with ProteinMPNN and AlphaFold2. The crystal structure of ASGPR (PDB 5JQ1) was refined and helical protein scaffolds were docked against the exposed hydrophobic residues via Patchdock followed by Rifdock. The sequences were optimized with protein-MPNN and interface scores were calculated with Rosetta Fastrelax. The models were then predicted by AlphaFold2 and scored after Fastrelax. Designs with pae_interaction<10 and relaxed_ddg < −40 were selected for resampling with another round of protein-MPNN prediction followed by Rosetta Fastrelax. After final round filtering with pae_interaction, relaxed_ddg and sap_score, the sequences were further optimized to have a net-charge of −7. The final designed sequences for AS_EndoTag are provided in Supplementary Table 4.

### Computational design of IGF_EndoTags

To generate flexible IGF2R agonists, all combinations of GS linkers with various lengths linking D6mb and D11mb were modelled with AlphaFold2^25^. The designs with poor monomer plddt (plddt < 85) were dropped.

To generate rigid IGF_EndoTags, the major binding helix from D11mb or the native IGF2-binding helix, and two interface helices from D6mb were extracted. Crystal structures obtained for D6mb and D11mb in complex with IGF2 and IGF2R were used as starting points for design. Domains 6 and 11 of the complex structures were aligned with the respective domains of IGF2R in the putative receptor internalizing conformation available in the Protein Data Bank (PDB: 6UM2). In this orientation, the two interface helices from D6mb and the single interface helix from D11mb were extracted and used as motifs to scaffold by protein inpainting^24^. To increase the likelihood of design success, the D11mb structure was adjusted to form an ideal three helical bundle with the two domain 6 helices. Protein inpainting was implemented such that the interacting residues within 3 Å of the receptor maintained the same identity as in the original minibinders. To increase design diversity, the domain 11 helix motif was randomly perturbed by rigid-body translations (up to 5 Å) and rotations (up to 10 radians) for each design prior to inpainting a scaffold between the motifs. The best inpainting outputs were selected by RosettaFold LDDT metrics (>0.5) for the inpainted region and used for sequence design with ProteinMPNN. ProteinMPNN sequence design was performed on the inpainted outputs in their desired complex orientation (with both domains 6 and 11 present) while fixing the original minibinder identities of interface residues (D6mb: R4, V8, Q11, D15, V20, K24, M25, I27, I31 and E34; D11mb: M1, A4, L7, L8 and W11). After 2,000 sequences were generated for each ProteinMPNN input, designs were filtered by predicted Rosetta ddG. AlphaFold2 structure predictions of the designed sequences were filtered by the pLDDT metric (keeping those with pLDDT > 90), and designs with a sub-angstrom backbone atom root mean squared deviation to the original design models realigned to D6mb and D11mb crystal structures (in complex with the IGF2–M6PR target domains). Finally, the complexes were assessed by Rosetta FastRelax. Designs with ddG metrics less than −40 and spatial aggregation propensity scores less than 35 were selected for expression and experimental assays.

### N-linked glycan verification of epitope

To verify the epitope of the designed binders, an N-linked glycan scan was performed. This was performed to rapidly determine if the computational designed binder was interacting with the chosen interface^50^. Four engineered N-linked glycan variants (NN-0975, NN-0979, NN-0981 and NN-0977) with mutation close to the Sort_EndoTag-binding site were designed and expressed. For design, the computational models were used as a starting point for the computational screen. All positions 10 Å away from the interface were screened using RosettaMatch^51^ followed by a design step to introduce the NXS/T motif into the protein. Computational models were minimized and filtered based on geometrical restraints, CST-score <5. Next, the four variants were used as bait in the yeast display assay against the computational designed binder, Sort_EndoTag, which was displayed on the surface of yeast.

### Yeast surface display screening with FACS

The yeast surface display screening was performed as described^15,17^. In brief, DNAs encoding the minibinder sequences were transformed into EBY-100 yeast strain. The yeast cells were grown in CTUG medium and induced in SGCAA medium. After washing with PBSF (PBS + 1% BSA), the cells were incubated with 1 μM biotinylated target proteins (IGF2R, ASGPR or sortilin) together with Streptavidin–phycoerythrin (SAPE, Thermo Fisher, 1:100) and anti-Myc fluorescein isothiocyanate (FITC, Miltenyi Biotech, 6.8:100) for 30 min. After washing twice with PBSF, the yeast cells were then resuspended in PBSF and screened via FACS. Only cells with PE and FITC double-positive signals were sorted for next-round screening. After another round of enrichment, the cells were titrated with biotinylated target protein at different concentrations for 30 min, washed, and further stained with both Streptavidin–phycoerythrin (SAPE, Thermo Fisher) and anti-Myc fluorescein isothiocyanate (FITC, Miltenyi Biotech) at 1:100 ratio for 30 min. After washing twice with PBSF, the yeast cells at different concentrations were sorted individually via FACS and regrown for 2 days. Next the cells from each subpool were lysated and their sequences were determined next-generation sequencing or MiSeq. FACS data were collected with the Sony SH800 software suite.

For N-linked glycan verification, yeast cells displaying Sort_EndoTag were incubated with 100nM N-glycan variants of sortilin (NN-0975, NN-0979, NN-0981 and NN-0977), separately. The percentage of yeast cells located within the pre-set gate was calculated for each N-glycan variants group and compared with the wild-type sortilin group.

### Biolayer interferometry

The binding affinity for the minibinders were determined using an Octet RED96 (ForteBio). To measure the binding affinity, Streptavidin-coated biosensors (ForteBio) were first loaded with biotinylated target proteins at 50–100 nM concentration, washed with Octet buffer (10 mM HEPES, 150 mM NaCl, 3 mM EDTA, 0.05% surfactant P20 and 1% BSA), and incubated with titrated concentrations of corresponding binders. To measure the off rate (*K*_off_), the biosensors were then dipped back into the Octet buffer. The on rate (*K*_on_), *K*_off_ and *K*_d_ were further estimated with the Octet Analysis software.

### Protein production and purification

Minibinders and minibinder fusions were expressed in *E. coli* BL21 as previously described^1^. In brief, the DNA fragments encoding the design sequences were assembled into PET-29 vectors via Gibson assembly and further transformed into BL21 strain with heat-shock. Protein expression was induced by the autoinduction system and proteins were purified with Immobilized metal affinity chromatography (IMAC) approach. Next the elutions were purified by FPLC SEC using Superdex 75 10/300 GL column (GE Healthcare). Protein concentrations were determined by NanoDrop (Thermo Scientific) and normalized by extinction coefficients.

Antibody–EndoTag fusions were produced with a mammalian expression system. Light chain of CTX/ATZ antibody and heavy chain fused with EndoTag at C-terminal constructs were ordered in CMVR from Genscript. Antibody–EndoTag fusions were then expressed via transient co-transfection of the EndoTag-heavy and light chains into Expi293F cells (Life Technologies) via PEI-MAX (Polyscience). In brief, 800 ml cultures of Expi293F cells were transfected at a density of 3 × 10^6^ cells per millilitre of culture using 1 μg plasmid DNA and 3 μg PEI per millilitre of culture. These cultures were grown in Expi293F expression medium (Life Technologies) at 37 °C in a humidified, 8% CO_2_ incubator rotating at 125 rpm.

After 6 days of expression, culture supernatants were harvested via 5 min of centrifugation at 4,000*g*, 5 min of incubation with PDADMAC solution (Sigma Aldrich) added to a final concentration of 0.0375%, followed by an additional 5 min of centrifugation at 4,000*g*. Supernatants were clarified via 0.22-μm vacuum filtration and then treated to a final concentration of 50 mM Tris-HCl (pH 8) and 350 mM NaCl for IMAC. Gravity IMAC was performed by batch binding the clarified supernatants with 10 ml of Ni Sepharose Excel resin (GE Healthcare). After 20–30 min of incubation, the resin bed was washed with 10 column volumes of 20 mM Tris-HCl (pH 8), 300 mM NaCl solution. The proteins were then eluted with 3 column volumes of 20 mM Tris-HCl (pH 8), 300 mM NaCl, 300 mM imidazole solution. The batch bind process was then repeated with half the amount of resin (5 ml) and the eluates from both batch binds were combined. SDS–PAGE was performed on the IMAC eluates to assess purity.

The purified antibody–EndoTag fusions were subsequently concentrated in a 10 K MWCO Amicon Ultra centrifugal filter unit (Millipore) and polished via SEC using a Hiload 26/600 Superose 200 column (GE Healthcare) in DPBS (Gibco). The SEC fractions were re-concentrated in the same manner as before to a final concentration of 5 mg ml^−1^. Endotoxin levels were assayed via Endosafe LAL Endotoxin tests (Charles River) and analytical SEC was performed using a Superdex 200 Increase 5/150 column (GE Healthcare) to obtain a high-resolution size profile. Pre- and post-freeze stability was assessed via UV-vis spectrophotometry as well as SDS–PAGE.

### Cellular uptake evaluation and receptor degradation via flow cytometry

For cellular uptake assays using suspension cell lines (K562, Jurkat), the cells were incubated with corresponding fluorescence-labelled protein constructs at 37 °C for indicated time, then spun down at 500*g* for 5 min, resuspended and washed with cold PBS. After three washes, the cells were resuspended and transferred to a 96-well plate. For cellular uptake assays using adherent cell lines (U-251MG, Hep3B, HeLa and H1975), the cells were incubated with corresponding fluorescence-labelled protein constructs at 37 °C for indicated time, then washed with cold PBS for three times. The cells were then treated with 50 μl trypsin and incubated at 37 °C for 10 min followed by adding 50 μl DMEM. The resuspended cells were then transferred to a 96-well plate followed by 2 PBS washes. Flow cytometry was then performed in Attune NxT flow cytometer (Thermo Fisher). The data were analysed in FlowJo v9 software.

For cell surface receptor-degradation experiments, the cells were first incubated with corresponding protein reagents for indicated time at 37 °C, then washed with cold PBS 3 times. For suspension cell lines, the cells were resuspended and transferred to the 96-well plate; for adherent cell lines, the cells were first treated with trypsin for 10 min then transferred to the 96-well plate. The cells were then stained with corresponding fluorescence-labelled antibodies against the corresponding receptor for 1 h at room temperature. After washing three times with cold PBS for flow cytometry, flow cytometry was performed in Attune NxT flow cytometer (Thermo Fisher). The data were analysed in FlowJo v9 software. Representative gating strategy for flow cytometry is provided in Supplementary Figs. 1–17.

### Monitoring protein degradation via western blot

Cells were cultured in T75 flasks at 37 °C in a 5% CO_2_ atmosphere. HEP3B (ATCC), HeLa (ATCC), and MDA-MB-231 were cultured in DMEM supplemented with 10% heat-inactivated fetal bovine serum (FBS) and 1% penicillin/streptomycin. Jurkat-CTLA4 (Promega, JA3001) and H1975 were cultured in RPMI supplemented with 10% heat-inactivated fetal bovine serum (FBS) and 1% penicillin/streptomycin. Adherent cells were plated (100,000 cells per well in a 24-well plate) one day before the experiment, whereas suspension cells were plated on the day of the treatment. Cells were incubated with 250 µl of complete growth media with pLYTAC or untreated controls for indicated time. Cells were then washed with PBS 3 times and lysed with RIPA buffer supplemented with protease inhibitor cocktail (Roche), 0.1% Benzonase (Millipore-Sigma), and phosphatase inhibitor cocktail (Roche) on ice for 30 min. The cells were scraped, transferred to Eppendorf tubes, and spun down at 21,000*g* for 15 min at 4 °C. The supernatant was collected and the protein concentration was determined by BCA assay (Pierce). Equal amounts of lysates were loaded onto 4–12% Bis-Tris gel and separated by SDS–PAGE. Then, the gel was transferred onto a nitrocellulose membrane and stained with REVERT Total Protein Stain (LI-COR), then blocked with Odyssey Blocking Buffer (TBS) (LI-COR) for 1 h at room temperature. The membrane was incubated with primary antibodies (rabbit anti-EGFR D38B1 Cell Signaling Technologies, rabbit anti-HER2 2242 Cell Signaling Technologies, rabbit anti-PD-L1 E1L3N Cell Signaling Technologies, rabbit anti-CTAL4 E1V6T Cell Signaling Technologies, mouse anti-vinculin V284 Bio-Rad) overnight at 4 °C, washed 3 times with TBST. Subsequently, the membrane was incubated with secondary antibody (800CW goat anti-mouse or goat anti-rabbit LI-COR 926-32211) for 1 h at room temperature, and washed 3 times with TBST for visualization with an Odyssey CLx Imager (LI-COR). Image Studio (LI-COR) was used to quantify band intensities. Full scans of western blot gels are provided in Supplementary Figs. 1–16.

### Fluorescence imaging

Wild-type HeLa (ATCC CCL-2) were cultured at 37 °C with 5% CO_2_ in flasks with Dulbecco’s modified Eagle medium (DMEM) (Gibco) supplemented with 1 mM l-glutamine (Gibco), 4.5 g l^−1^ d-glucose (Gibco), 10% fetal bovine serum (FBS) (Hyclone) and 1% penicillin-streptomycin (PenStrep) (Gibco). To passage, cells were dissociated using 0.05% trypsin EDTA (Gibco) and split 1:5 or 1:10 into a new tissue culture-treated T75 flask (Thermo Scientific ref 156499).

For imaging 35-mm glass bottom dishes were seeded at a density of 20,000 cells per dish. A final monomeric concentration of 100 nM of ligands were incubated with cultured cells. Cells were fixed 4% paraformaldehyde, permeabilized with 100% methanol, and blocked with PBS + 1% BSA. Cells were immunostained with LAMP2A antibody (Abcam ab18528) followed by goat anti-rabbit IgG Alexa Fluor 488 secondary antibody (Thermo Fisher A-11034) and 4′,6-diamidino-2-phenylindole (DAPI) (Thermo Fisher D1306) and stored in the dark at 4 °C until imaging.

Cells were washed twice with HBSS and subsequently imaged in HBSS in the dark at 37 °C. Right before imaging, cells were incubated with 25 µM DTZ. Epifluorescence imaging was conducted on a Yokogawa CSU-X1 microscope equipped with a Hamamatsu ORCA-Fusion scientific CMOS camera and Lumencor Celesta light engine. Objectives used were: 10×, NA 0.45, WD 4.0 mm, 20×, NA 1.4, WD 0.13 mm, and 40×, NA 0.95, WD 0.17–0.25 mm with correction collar for cover glass thickness (0.11 mm to 0.23 mm) (Plan Apochromat Lambda). All epifluorescence experiments were subsequently analysed using NIS Elements software.

### Generation of knockout lines

IGF2R-knockout HeLa cells were a generous gift form S. Banik. SORT1 and TfR KO cells were generated using Gene Knockout Kit v2 (Synthego) using the manufacturer’s protocols.

### Confocal microscopy

Indicated cells were seeded in 18-well glass bottom µ-Slides (Ibidi, 81817) at a density of 15,000 cells per well. Fluorescently labelled ligands were incubated with the cultured cells for 0.25, 3, 6 or 24 h. Thirty minutes before image acquisition, cells were additionally incubated with LysoTracker (Thermo Fisher Scientific, L7528, L7526, L12492) was added for 30 min. Fluorescently labelled anti-IGF2R (Novus Biological, NB300-514AF647) was added for 30 min. Cells were washed 3× in PBS and immediately proceeded to imaging.

Confocal laser scanning microscopy was performed on a Nikon A1R HD25 system equipped with a LU-N4 laser unit (Lasers used: 488 nm, 561 nm, 640 nm). Data were acquired using a 20×, NA 0.75, WD 1.00 mm air objective (Plan Apochromat Lambda) in combination with 1 multialkaline (EM 650 LP) and 2 GaAsP detectors (DM 560 LP EM 524/42 (503-545) and DM 652 EM 600/45 (578-623)). Acquisition was controlled via NIS Elements software and data were analysed via Fiji and custom-written Python scripts.

### Mass spectrometry and proteomics

Cell pellets were thawed on ice and lysed in a lysis buffer (400 μl, 1 tablet of Pierce EDTA-free Protease Inhibitor Tablets dissolved in 50 ml of PBS) using a probe sonicator (3× 3 pulses). Protein concentration was adjusted to 2.0 mg ml^−1^ and the samples (100 μl, 200 μg protein) were transferred to new Eppendorf tubes (1.5 ml) containing urea (48 mg per tube, final urea concentration: 8 M). DTT (5 μl, 200 mM fresh stock in H_2_O, final DTT concentration: 10 mM) was then added to the tubes and the samples were incubated at 65 °C for 15 min. Following this incubation, iodoacetamide (5 μl, 400 mM fresh stock in H_2_O, final iodoacetamide concentration: 20 mM) was added and the samples were incubated in the dark at 37 °C with shaking for 30 min. Ice-cold methanol (600 μl), CHCl_3_ (200 μl), and H_2_O (500 μl) were then added, and the mixture was vortexed and centrifuged (10,000*g*, 10 min, 4 °C) to afford a protein disc at the interface between CHCl_3_ and aqueous layers. The top layer was aspirated without perturbing the disk, additional methanol (600 μl) was added, and the proteins were pelleted (10,000*g*, 10 min, 4 °C) and used in the next step or stored at −80 °C overnight.

The resulting protein pellets were resuspended in EPPS buffer (160 μl, 200 mM, pH 8) using probe sonicator (3× 3 pulses). Trypsin (10 μl, 0.5 μg μl^−1^ in trypsin reconstitute buffer) and CaCl_2_ (1.8 μl, 100 mM in H_2_O) were added and the samples were incubated at 37 °C with shaking overnight.

Peptide concentration was determined using the microBCA assay (Thermo Scientific) according to the manufacturer’s instructions. For each sample, a volume corresponding to 25 μg of peptides was transferred to a new Eppendorf tube and the total volume was brought up to 35 μl with EPPS buffer (200 mM, pH 8). The samples were diluted with CH_3_CN (9 μl) and incubated with the corresponding TMT tags (3 μl per channel, 20 μg μl^−1^) at room temperature for 30 min. An additional TMT tag (3 μl per channel, 20 μg μl^−1^, 30 min) was added and the samples were incubated for another 30 min. Labeling was quenched by the addition of hydroxylamine (6 μl, 5% in H_2_O). Following a 15 min incubation at room temperature, formic acid was added (2.5 μl, final formic acid concentration: 5%). Twenty microlitres of labelled peptides for each channel were combined into a 2.0 ml low-binding Eppendorf tube, and 25 μl of 20% formic acid was added. The resulting mixture was lyophilized to remove the solvents before high pH fractionation.

The spin columns from Pierce High pH Reversed-Phase Peptide Fractionation Kit were pre-equilibrated prior to use. In brief, the columns were placed in Eppendorf tubes (2 ml), spun down to remove the storage solution (5,000*g*, 2 min), and washed with CH_3_CN (2× 300 μl, 5,000*g*, 2 min) and buffer A (2× 300 μl, 95% H_2_O, 5% CH_3_CN, 0.1% formic acid, 5,000*g*, 2 min). TMT-labelled peptides were re-dissolved in buffer A (300 μl, 95% H_2_O, 5% CH_3_CN, 0.1% formic acid) and loaded onto pre-equilibrated spin columns for high pH fractionation. The columns were spun down (2,000*g*, 2 min) and the flow through was used to wash the original Eppendorf tube and passed through the spin column again (2,000*g*, 2 min). The column was then washed with buffer A (300 μl, 2,000*g*, 2 min) and 10 mM aqueous NH_4_HCO_3_ containing 5% CH_3_CN (300 μl, 2,000*g*, 2 min), and the flow through was discarded. The peptides were eluted from the spin column into fresh Eppendorf tubes (2.0 ml) with a series of 10 mM NH_4_HCO_3_/CH_3_CN buffers (2,000*g*, 2 min). The following buffers were used for peptide elution (CH_3_CN (%)): 7.5, 10, 12.5, 15, 17.5, 20, 22.5, 25, 27.5, 30, 32.5, 35, 37.5, 40, 42.5, 45, 47.5, 50, 52.5, 55, 57.5, 60, 62.5, 65, 67.5, 70, 72.5, 75, 80 and 95. Every tenth fraction was combined into a new clean Eppendorf tube (2 ml) and the solvent was removed using a benchtop lyophilizer and stored at −20 °C before analysis.

The resulting 10 combined fractions were resuspended in buffer A (25 μl) and analysed on the Orbitrap Fusion mass-spectrometer (4 μl injection volume) coupled to a Thermo Scientific EASY-nLC 1200 LC system and autosampler. The peptides were eluted onto a capillary column (75 μm inner diameter fused silica, packed with C18) and separated at a flow rate of 0.3 μl/min^−1^ using the following gradient: 5% buffer B in buffer A from 0–10 min, 5%–35% buffer B from 10–129 min, 35%–100% buffer B from 129–130 min, 100% buffer B from 130–139 min, 100%–5% buffer B from 139–140 min, and 5% buffer B from 140–150 min (buffer A: 100% H_2_O, 0.1% formic acid; buffer B: 20% H_2_O, 80% CH_3_CN, 0.1% formic acid). Data were acquired using an MS3-based TMT method. In brief, the scan sequence began with an MS1 master scan (Orbitrap analysis, resolution 120,000, 375 − 1,600 *m*/*z*, cycle time 3 s) with dynamic exclusion enabled (repeat count 1, duration 30 s). The top precursors were then selected for MS2/MS3 analysis. MS2 analysis consisted of: quadrupole isolation (isolation window was set to 1.2 for charge state *z* = 2; 0.7 for charge state *z* = 3; 0.5 for charge states *z* = 4–6) of precursor ion followed by collision-induced dissociation (CID) in the ion trap (normalized collision energy 35%, maximum injection time 50 ms, MS2 resolution was set to turbo). Following the acquisition of each MS2 spectrum, synchronous precursor selection (SPS) enabled the selection of MS2 fragment ions for MS3 analysis (SPS isolation window was set to 1.3 for charge state *z* = 2; 0.7 for charge state *z* = 3; 0.5 for charge states *z* = 4–6). MS3 precursors were fragmented by HCD and analysed using the Orbitrap (collision energy 65%, maximum injection time 120 ms). The raw files were converted to mzML files using the MSConvert tool from ProteoWizard (version 3.0.22088). A reverse concatenated, non-redundant variant of the Human UniProt database (29 November 2022) was searched using FragPipe (version 18.0) with the built-in TMT10-MS3 workflow. The virtual references were used for the data sets due to the lack of a pooled sample. The quantified proteins were filtered with false discovery rate < 1% with median centreing normalization. Data are presented as the mean fold change to DMSO-treated controls. *n* = 3 per group. *P* values were calculated by a two-tailed unpaired *t*-test with Welch’s correction.

### IgG and LHDB supernatant clearance assays

Jurkat or K562 cells seeded in 96-well culture plates in 300 μl medium were incubated with AF647-conjugated IgG (Novusbio) or LHDB alone or together with protein G-EndoTag reagents. At various timepoints, the cells were pelleted down and 30 μl of supernatants were extracted and further diluted to 45 μl by using a PBS buffer. After shaking in an orbital shaker for 5 min, the fluorescence intensity was measured using a Neo2 plate reader (BioTek) at wavelength 647 nm. The percentage clearance was measured by normalizing the control group without adding protein G-EndoTag reagent.

### SEC binding assay

ASGPR protein (28.4 kDa) at 1 μM (diluted in PBS) was incubated with 3 μM AS_EndoTag-3C for 30 min and run through an ÄKTA SEC protein purification system using a S200 16/90 column. The absorbance at 230 nm was used as a readout for binding. The SEC traces of the complex was compared to the traces of individual ASGPR or AS_EndoTag-3C at same concentration.

### In vivo mouse study

Mouse lymphoma cell line A20 cell was purchased from American Type Culture Collection (ATCC, TIB-208). The cell was cultured in RPMI-1640 medium (Gibco, Thermo Scientific, 31870074), supplemented with 10% heat-inactivated fetal bovine serum (FBS) (Gibco, Thermo Scientific), 1× GlutaMAX (Gibco, Thermo Scientific), 1× penicillin/streptomycin solution (Gibco, Thermo Scientific). The cell was cultured at 37 °C in humidified condition with 5% CO_2_.

All animal experiments were conducted at the Instituto de Medicina Molecular João Lobo Antunes (IMM), Lisbon. Animal work was performed in strict accordance with Portuguese Law (Portaria 1005/92) and the European Guideline 86/609/EEC and follow the Federation of European Laboratory Animal Science Associations guidelines and recommendations concerning laboratory animal welfare. All animal experiments were approved by the Portuguese official veterinary department for welfare licensing (Direção Geral de Alimentação e Veterinária) and the IMM Animal Ethics Committee (authorization AWB_2021_03_GB_Targ CancerDrugs). Eight-week-old female BALB/c mice (purchased from Charles River) were used in this study, with 5 × 10^6^ A20 cells inoculated subcutaneously in the flank. Tumour growth was monitored over time, by performing bilateral vernier caliper measurements every day and mean tumour volumes were calculated using the formula (length × width^2^)/2. Treatments were initiated when tumours reached approximately 100 mm^3^ (approximately 10 days after tumour induction), with the mice been randomly assigned to receive ATZ, ATZ–IGF_EndoTag1 or ATZ–IGF_EndoTag4 and isotype as controls (*n* = 6 mice per group). Treatments were administered intratumourally in a total of three injections for every three days. Animals were monitored every day; tumours were measured as described before and mouse weight was evaluated throughout the study. Animals were killed whenever reaching humane endpoints: loss of 20% of body weight, breathing impairment, or poor reaction to external stimuli. No signs of animal suffering or discomfort were observed during the experiment. For efficacy study, once control (isotype-treated mice) tumours reached 1,000 mm^3^, all mice were killed (by isoflurane overdose), and the tumours were removed for western blot analysis. For survival monitor, each mouse was killed respectively when tumours reached 1,000 mm^3^. The light/dark cycle was 14 h light/10 h dark (lights on at 07:00; lights off at 21:00). The temperature was 20–24 °C and the relative humidity was 55 ± 10%, with controlled supply of HEPA-filtered air provided to individually ventilated cages. Maximum number of animals per cage was five. Social isolation was avoided whenever possible. The type of food was autoclaved diet pellets RM3A (P), from SDS Special Diets Services (801030). Food was placed in a grid inside the cage and provided ad libitum to animals. The type of water was sterile water treated by reverse osmosis. Water was provided ad libitum to animals through bottles with a capillary hole. The data collected was analysed using GraphPad Prism9.

### In vivo PD-L1 degradation of tumour samples by western blot

Tumour samples isolated from the mice were homogenized, lysed in RIPA buffer containing protease inhibitor (Roche), phosphatase inhibitors (Sigma) and 0.1% Benzonase (Sigma) on ice for 30 min. The lysates were spun at 21,000*g* for 15 min at 4 °C. The supernatant was collected, and the protein concentrations were quantified using BCA assay (Sigma). Fifty micrograms of protein were loaded per lane and separated on 12% SDS–PAGE gels, and then transferred onto polyvinylidene difluoride (PVDF) membranes (GE Healthcare). Membranes were then blocked with 5% BSA in TBS supplemented with 0.1% Tween-20 (TBST) for 1 h at room temperature, and then probed with following specific primary antibodies at 4 °C overnight. After three times of washing with TBST, secondary antibodies were added to the membrane for 1 h at room temperature. All membranes were washed three times and exposed using ECL substrate (Bio-Rad, 170–5060) and Amersham 800 Imaging System (Cytiva). The primary antibodies used included PD-L1 (sc-518027) and beta-actin (sc-47778), the secondary antibody was goat anti-mouse IgG H&L (HRP) (Abcam, ab205719). The intensities of the bands were quantified by ImageJ.

### Statistical analysis

No statistical analysis was used to determine the sample size. The sample size was determined by our ability to detect meaningful differences between treatments. Western blot experiments in vitro and BLI binding assays were conducted with sample size of one based on low variance from previous experience. Western blot experiments in mice were performed with sample size of 3 to reduce the variance of protein level across animals from our previous best practice. The data were collected as biological replicates as indicated in the figure legend. All cell experiments were done multiple times to ensure reproducibility. All images were representative of three independently replicated samples. Statistical analyses are specified in figure legends.

### Reporting summary

Further information on research design is available in the Nature Portfolio Reporting Summary linked to this article.

## Online content

Any methods, additional references, Nature Portfolio reporting summaries, source data, extended data, supplementary information, acknowledgements, peer review information; details of author contributions and competing interests; and statements of data and code availability are available at https://doi.org/10.1038/s41586-024-07948-2.

## Supplementary information

Supplementary Information This file contains Supplementary Figs. 1–23 and Supplementary Tables 1–5.

Reporting Summary

Peer Review File

## Source data

Source Data Fig. 2

## Figures and Tables

**Extended Data Table 1:**
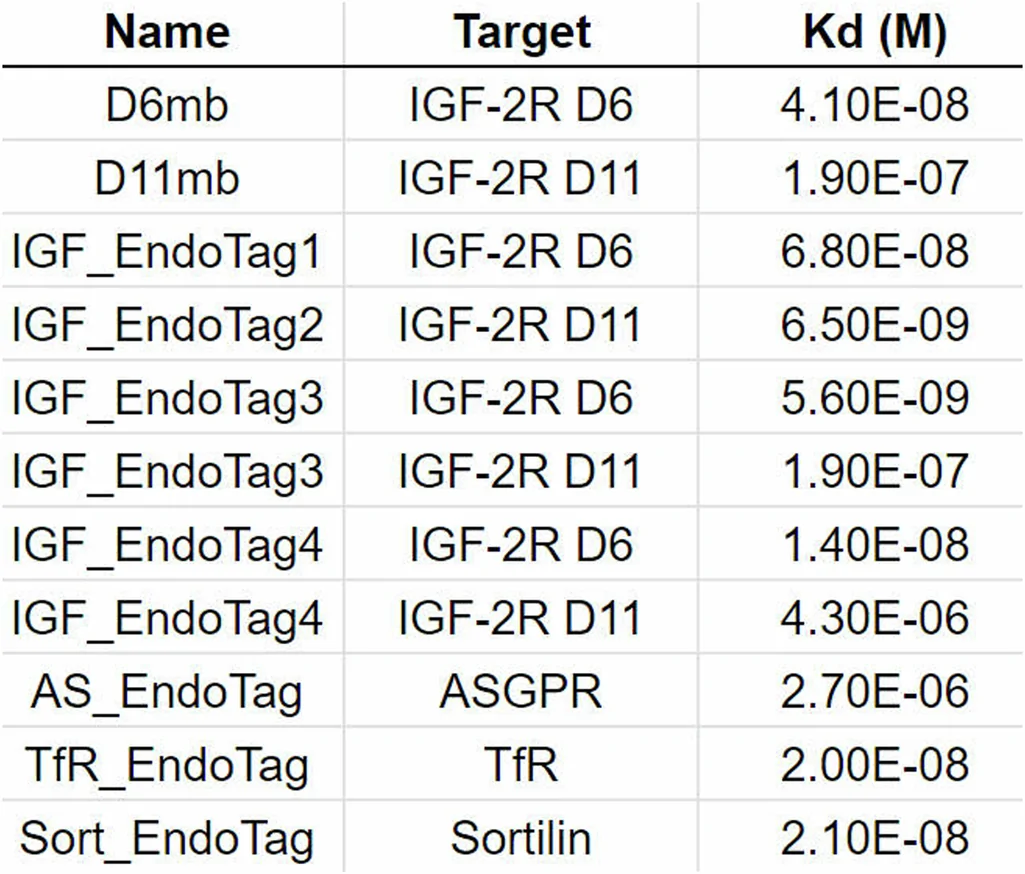
Binding affinity of EndoTag binders

All affinity data was collected by Octet R8 and binding affinity was estimated by Octet ForteBio software package.

## Data Availability

The raw data for the flow cytometry, next-generation sequencing, designed binder models and sequences are available at https://doi.org/10.5281/zenodo.11002950 (ref. ^52^). Source data are provided with this paper.

## Code availability

The Rosetta modelling suite (https://www.rosettacommons.org) is available to academic and non-commercial users for free. Commercial licences for the suite are also available through the University of Washington Technology Transfer Office. The source code for RIF docking is available at https://github.com/rifdock/rifdock. The source code for protein inpainting is available at https://github.com/RosettaCommons/RFDesign. The source code for ProteinMPNN is available at https://github.com/dauparas/ProteinMPNN. The scripts used in this paper for binder design applying the above codes, customized image processing and data processing are available at https://doi.org/10.5281/zenodo.11002950 (ref. ^52^).
