# Supplementary material for: Designed endocytosis-inducing proteins degrade targets and amplify signals
Source: Nature. 2024 Sep 25;638(8051):796–804. doi: 10.1038/s41586-024-07948-2 (PMC11839401; doi:10.1038/s41586-024-07948-2)
Supplement: Supplementary file 1 [file 41586_2024_7948_MOESM1_ESM.pdf]

nature portfolio

https://doi.org/10.1038/s41586-024-07948-2

Supplementary information

Designed endocytosis-inducing proteins degrade targets and amplify signals

In the format provided by the authors and unedited

Supplementary information for “Designed Endocytosis Inducing Proteins Degrade Targets and Amplify Signals”

Authors: Buwei Huang[1,2,3†], Mohamad Abedi[1,2†], Green Ahn[4†], Brian Coventry[1,2,5†], Isaac Sappington[1,2], Cong Tang[6], Rong Wang[7], Thomas Schlichthaerle[1,2], Jason Z. Zhang[1,2], Yujia Wang[2], Inna Goreshnik[1,2,5], Ching Wen Chiu[2], Adam Chazin-Gray[1,2], Sidney Chan[2], Stacey Gerben[2], Analisa Murray[2], Shunzhi Wang[1,2], Jason O'Neill[8], Li Yi[8], Ronald Yeh[8], Ayesha Misquith[8], Anitra Wolf[8], Luke M. Tomasovic[9,10], Dan I Piraner[11], Maria J. Duran Gonzalez[11], Nathaniel R. Bennett[1,2], Preetham Venkatesh[1,2], Maggie Ahlrichs[2], Craig Dobbins[2], Wei Yang[1,2], Xinru Wang[1,2], Danny D. Sahtoe[12], Dionne Vafeados[2], Rubul Mout[13], Shirin Shivaei[14], Longxing Cao[15], Lauren Carter[2], Lance Stewart[2], Jamie B. Spangler[8], Kole T. Roybal[11], Per Jr. Greisen[8], Xiaochun Li[7], Gonçalo J.L. Bernardes[6,16], Carolyn R. Bertozzi[4,17,18], David Baker[1,2,5]*

Affiliations:
[1]Department of Biochemistry, University of Washington, Seattle, WA, USA
[2]Institute for Protein Design, University of Washington, Seattle, WA, USA
[3]Department of Bioengineering, University of Washington, Seattle, WA, USA
[4]Department of Chemistry, Stanford University, Stanford, CA, USA.
[5]Howard Hughes Medical Institute, University of Washington, Seattle, WA, USA
[6]Instituto de Medicina Molecular João Lobo Antunes, Faculdade de Medicina, Universidade de Lisboa, Lisbon, Portugal
[7]Department of Molecular Genetics, University of Texas Southwestern Medical Center, Dallas, TX, USA.
[8]Novo Nordisk A/S, Måløv, Denmark
[9]Departments of Biomedical Engineering and Chemical and Biomolecular Engineering, Johns Hopkins University, Baltimore, MD, USA
[10]Medical Scientist Training Program, Johns Hopkins University School of Medicine, Baltimore, MD, USA
[11]Department of Microbiology and Immunology, University of California San Francisco
[12]Hubrecht Institute, Utrecht, NL
[13]Harvard Medical School, Harvard University, Boston, Massachusetts, USA
[14]Division of Biology and Bioengineering, California Institute of Technology, Pasadena, CA, USA
[15]School of Life Sciences, Westlake University, Hangzhou, China
[16]Yusuf Hamied Department of Chemistry, University of Cambridge, Cambridge, UK
[17]Howard Hughes Medical Institute, Stanford, CA, USA.
[18]Sarafan ChEM-H, Stanford University, Stanford, CA 94305, USA.

[†]These authors contributed equally to this work.

*To whom correspondence should be addressed. Email: dabaker@uw.edu

Title: Designed Endocytosis Inducing Proteins Degrade Targets and Amplify Signals

Supplementary Figs 1-16 contain full scan of gels ran in this study. Supplementary Figs 16-23 contain additional data figures to support the main figures. Supplementary Table 1-4 contain sequence information of the designs.

Supplement Figure 1. Full scan for Figure 2b
Supplement Figure 2. Full scan for Figure 2c (left)
Supplement Figure 3. Full scan for Figure 2d (right)
Supplement Figure 4. Full scan for Figure 2e
Supplement Figure 5. Full scan for Figure 2d(left),2f(left) and Extended Data Fig. 6b
Supplement Figure 6. Full scan for Figure 2f(right)
Supplement Figure 7. Full scan for Figure 2h
Supplement Figure 8. Full scan for Extended Data Fig. 6a
Supplement Figure 9. Full scan for Extended Data Fig. 6c
Supplement Figure 10. Full scan for Extended Data Fig. 10
Supplement Figure 11. Full scan for Extended Data Fig. 11(left)
Supplement Figure 12. Full scan for Extended Data Fig. 11(right)
Supplement Figure 13. Full scan for Extended Data Fig. 12a
Supplement Figure 14. Full scan for Extended Data Fig. 12b
Supplement Figure 15. Full scan for Extended Data Fig. 12c
Supplement Figure 16. Full scan for Extended Data Fig. 15
Supplement Figure 17. Flow cytometry gating strategy (representative)
Supplement Figure 18. Confocal imaging of co-localization of EndoTag with lysosome
Supplement Figure 19. Epitope verification using N-linked glycan screen of computational designed Sortilin binder Sort_EndoTag using Yeast Display
Supplement Figure 20. EGFR degradation with EndoTag interface knockout scaffolds
Supplement Figure 21. IGF-2R expression level and degradation capacity across cell lines
Supplement Figure 22. Validation of knockout of TfR or sortilin in HeLa cells
Supplement Figure 23. ASGPR trimer formation mechanism of AS_EndoTag
Supplement Table 1. IGF_EndoTag sequences
Supplement Table 2. Sortilin_EndoTag sequences
Supplement Table 3. TfR_EndoTag sequences
Supplement Table 4. ASGPR_EndoTag sequences
Supplement Table 5. Highlighted quantitative proteomic analysis of significantly downregulated proteins

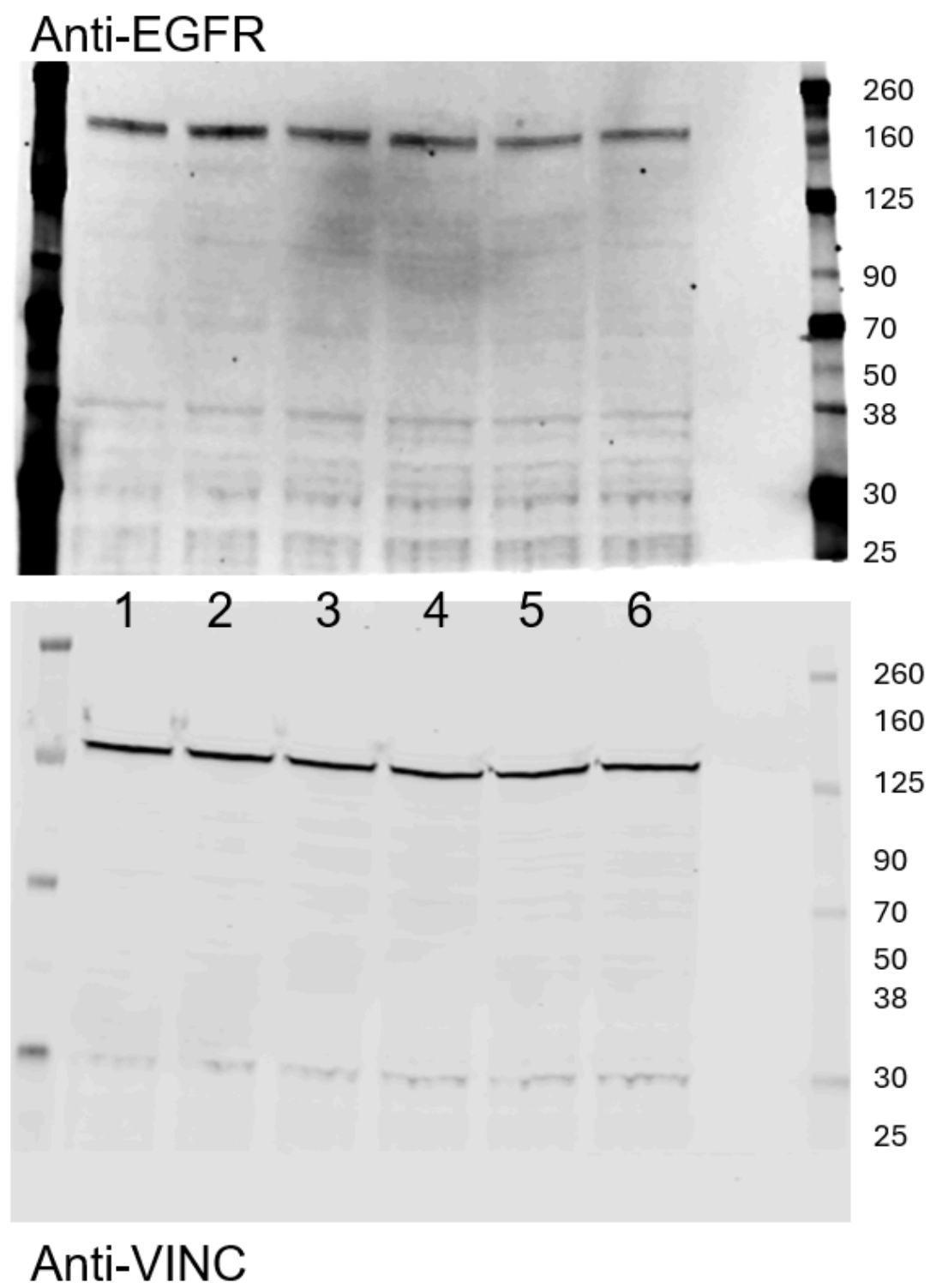

Supplement Figure 1. Full scan for Figure 2b

Annotations for each lane (left to right):

1:Untreated, 2:EGFRmb, 3:AS_EndoTag-EGFRn,

4:EGFRn-AS_EndoTag, 5:EGFRn-AS_EndoTag2C, 6:EGFRn-AS_EndoTag3C

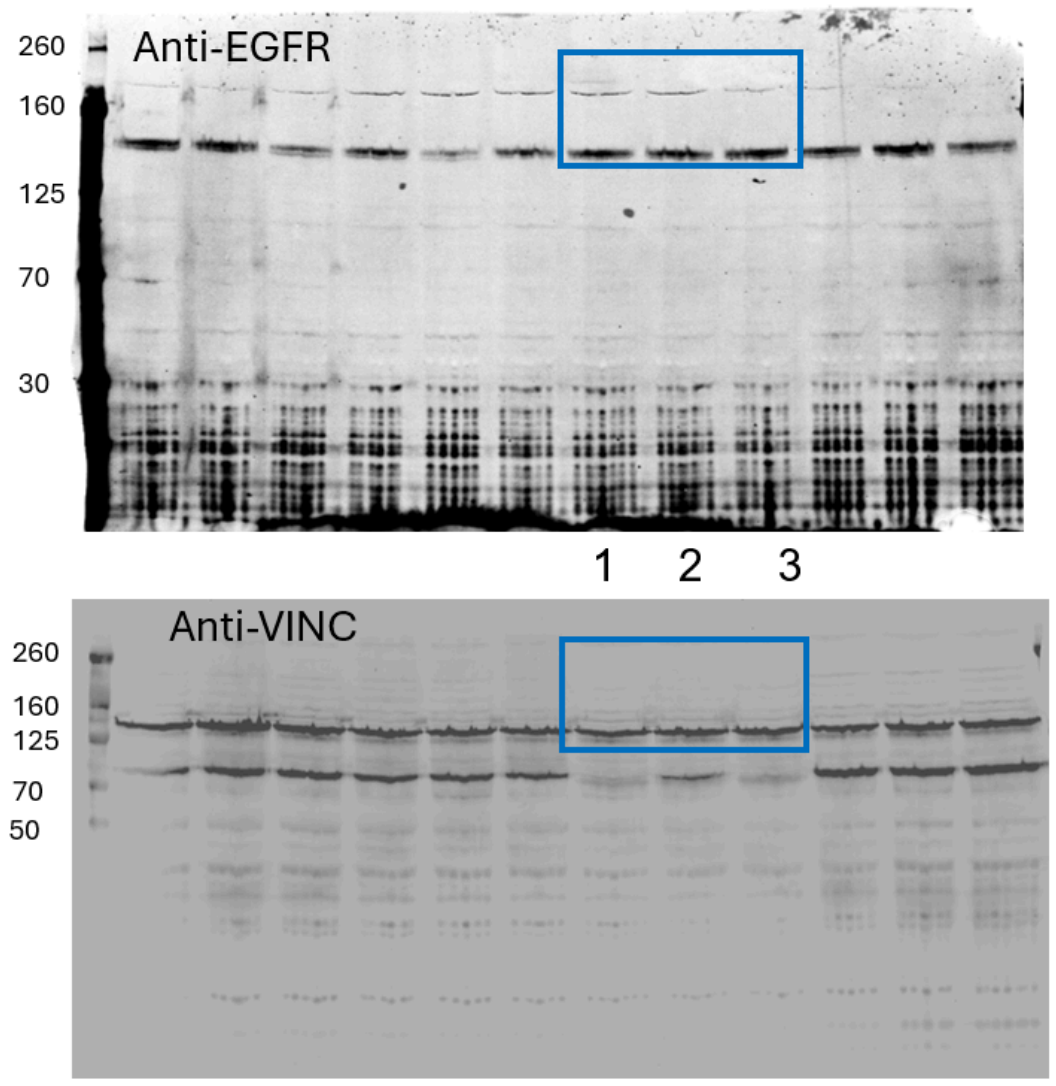

Supplement Figure 2. Full scan for Figure 2c (left)

Annotations for each lane:

1:Untreated, 2:EGFRmb, 3:EGFRmb-TfR_EndoTag

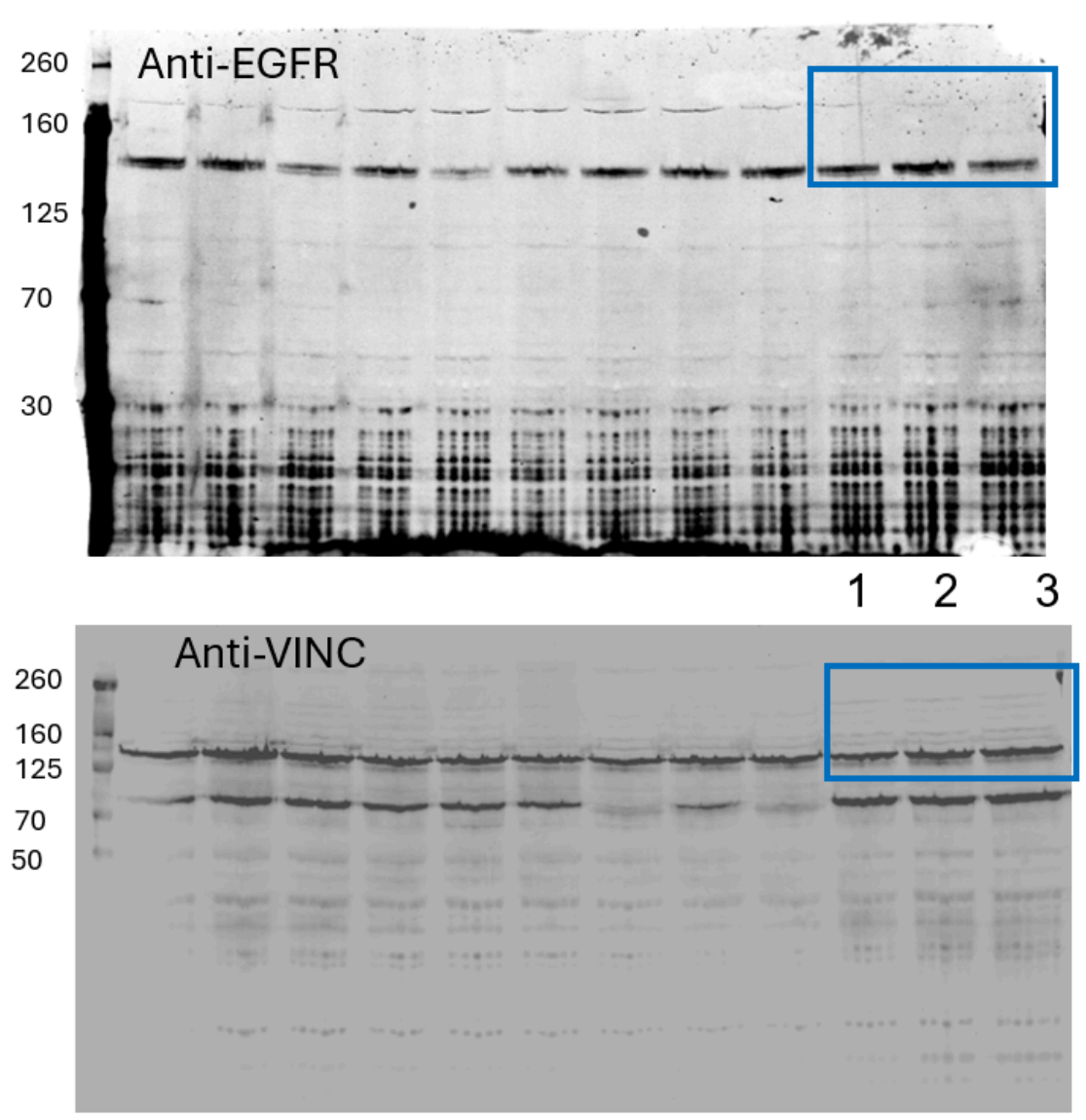

Supplement Figure 3. Full scan for Figure 2d (right)

Annotations for each lane:

1:Untreated, 2:EGFRmb, 3:EGFRmb-Sort_EndoTag

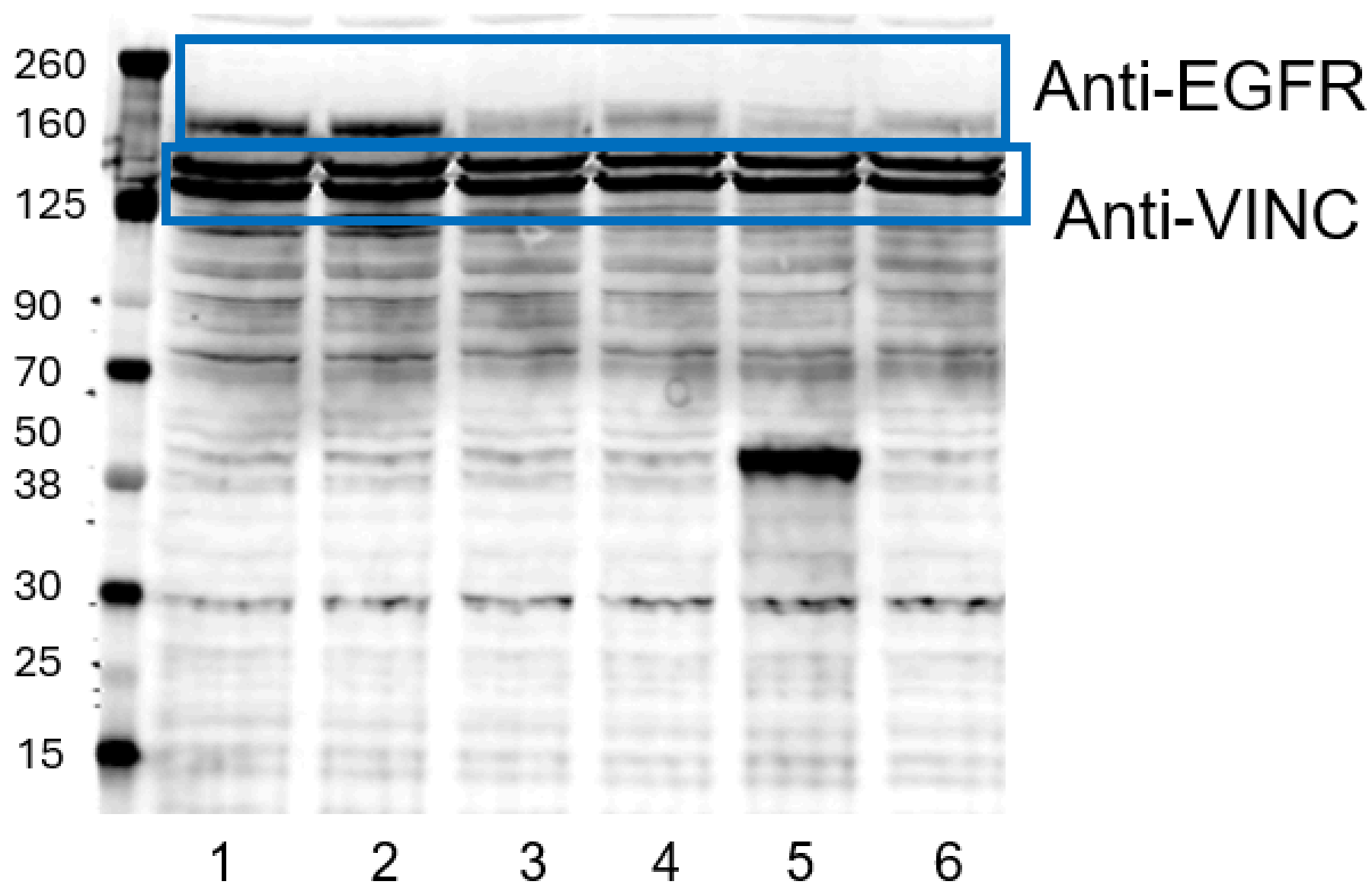

Supplement Figure 4. Full scan for Figure 2e

Annotations for each lane:

1:Untreated, 2:EGFRn, 3:EGFRn-IGF_EndoTag1,

4:Ctx, 5:Ctx-IGF_EndoTag1, 6:Ctx-M6pn

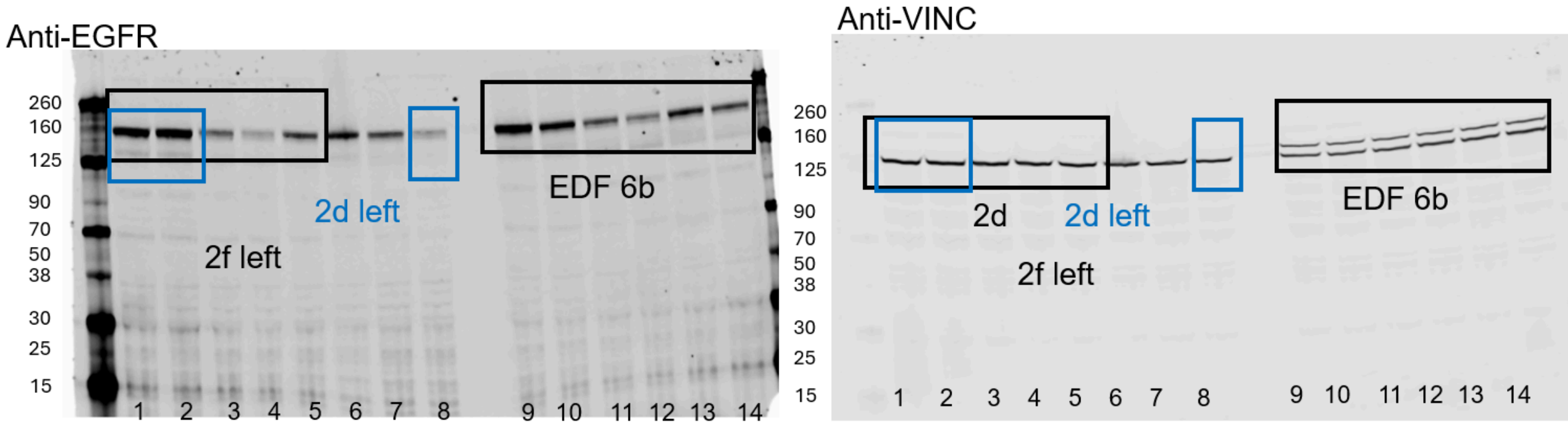

Supplement Figure 5. Full scan for Figure 2d(left),2f(left) and Extended Data Fig. 6b.

Annotations for each lane:

1:Untreated, 2:EGFRmb, 3:EGFRn-IGF_EndoTag1, 4:EGFRn-IGF_EndoTag2

5:EGFRn-IGF_EndoTag4, 8:EGFRn-Sort_EndoTag

9:Untreated, 10:EGFRnmb, 11:EGFRn-IGF_EndoTag1, 12:EGFRn-IGF_EndoTag2

13:EGFRn-IGF_EndoTag4, 14:EGFRn-Sort_EndoTag

The blue block is cropped for Fig.2d. The left black block is cropped for Fig.2f. The right black block is for Fig.2e and Extended Data Fig.6b

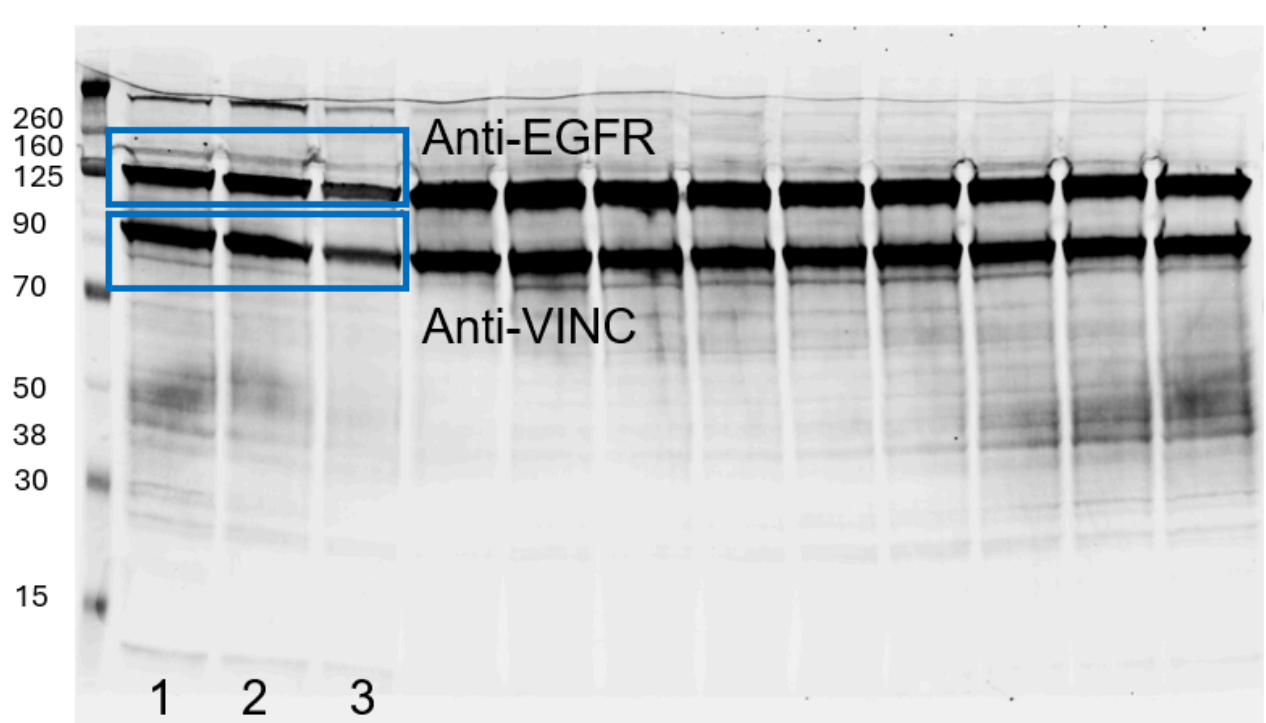

Supplement Figure 6. Full scan for Figure 2f(right)

Annotations for each lane:

1:Untreated, 2:EGFRmb, 3:EGFRmb-IGF_EndoTag2

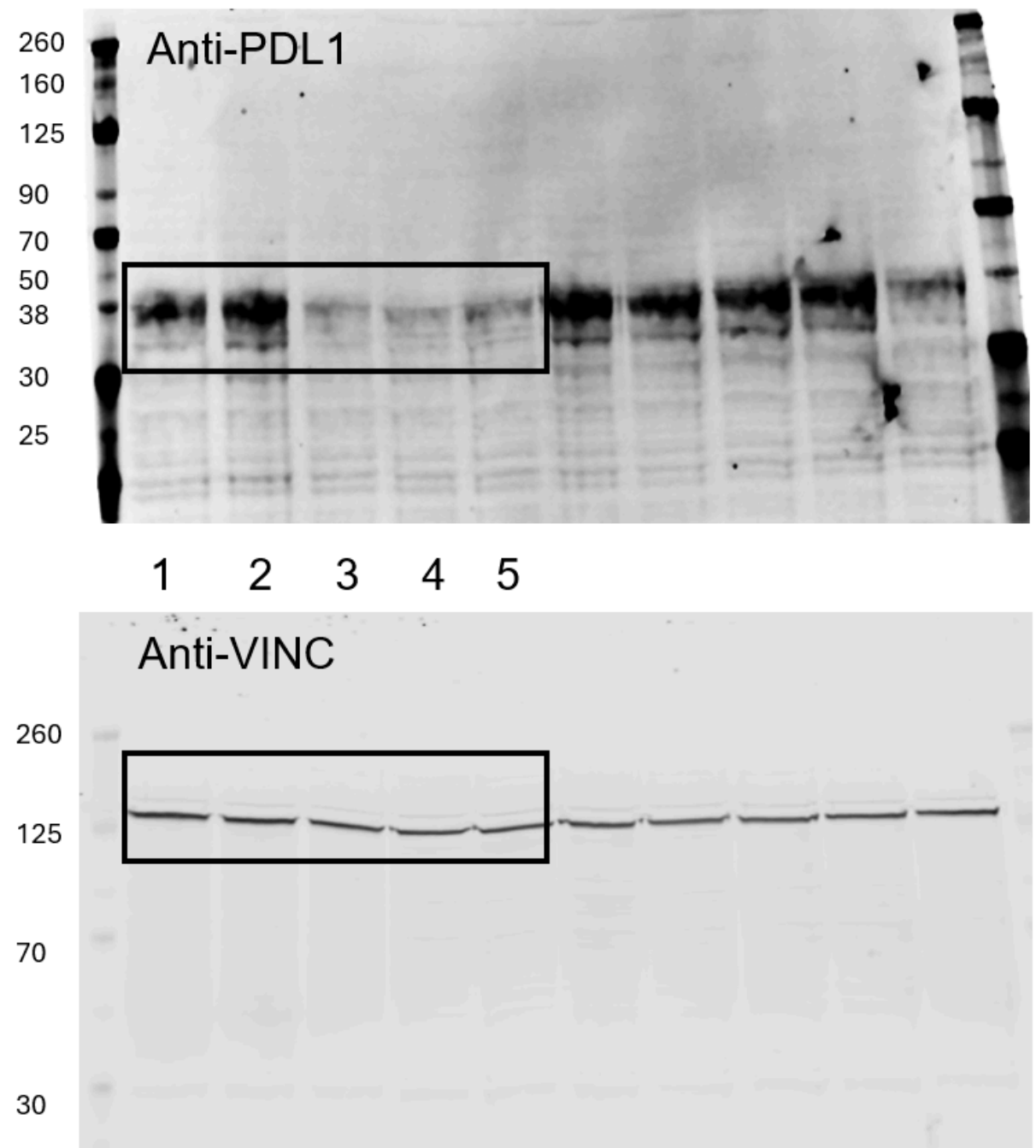

Supplement Figure 7. Full scan for Figure 2h

Annotations for each lane:

1:Untreated, 2:Atz, 3:Atz-IGF_EndoTag1, 4:Atz-IGF_EndoTag2, 5:Atz-IGF_EndoTag4

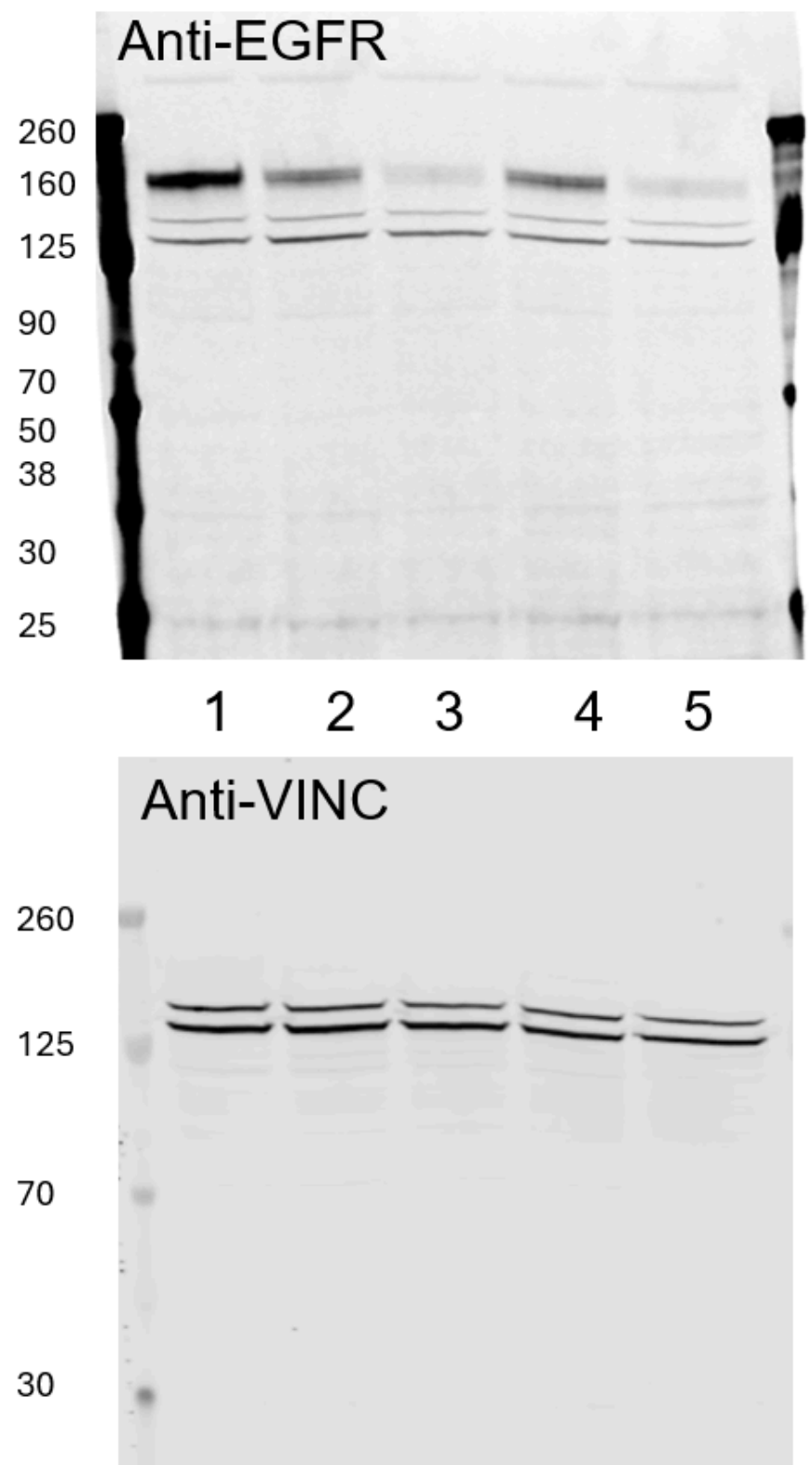

Supplement Figure 8. Full scan for Extended Data Fig. 6a

Annotations for each lane:

1:Untreated, 2:10nM Ctx, 3:10nM Ctx-IGF_EndoTag1

4:100nM Ctx, 5:100nM Ctx-IGF_EndoTag1

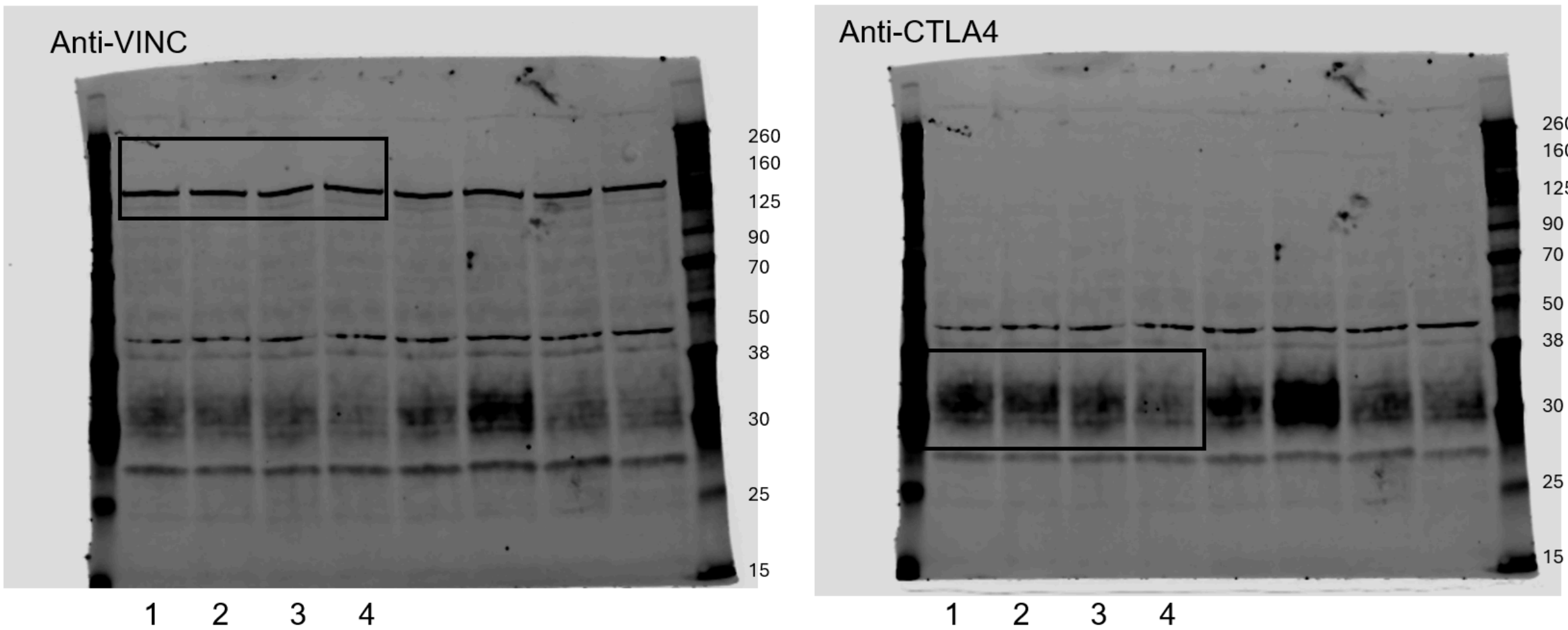

Supplement Figure 9. Full scan for Extended Data Fig. 6c

Annotations for each lane:

1:Untreated, 2:CTLA4mb, 3:CTLA4mb-IGF_EndoTag1, 4:IGF_EndoTag1-CTLA4mb

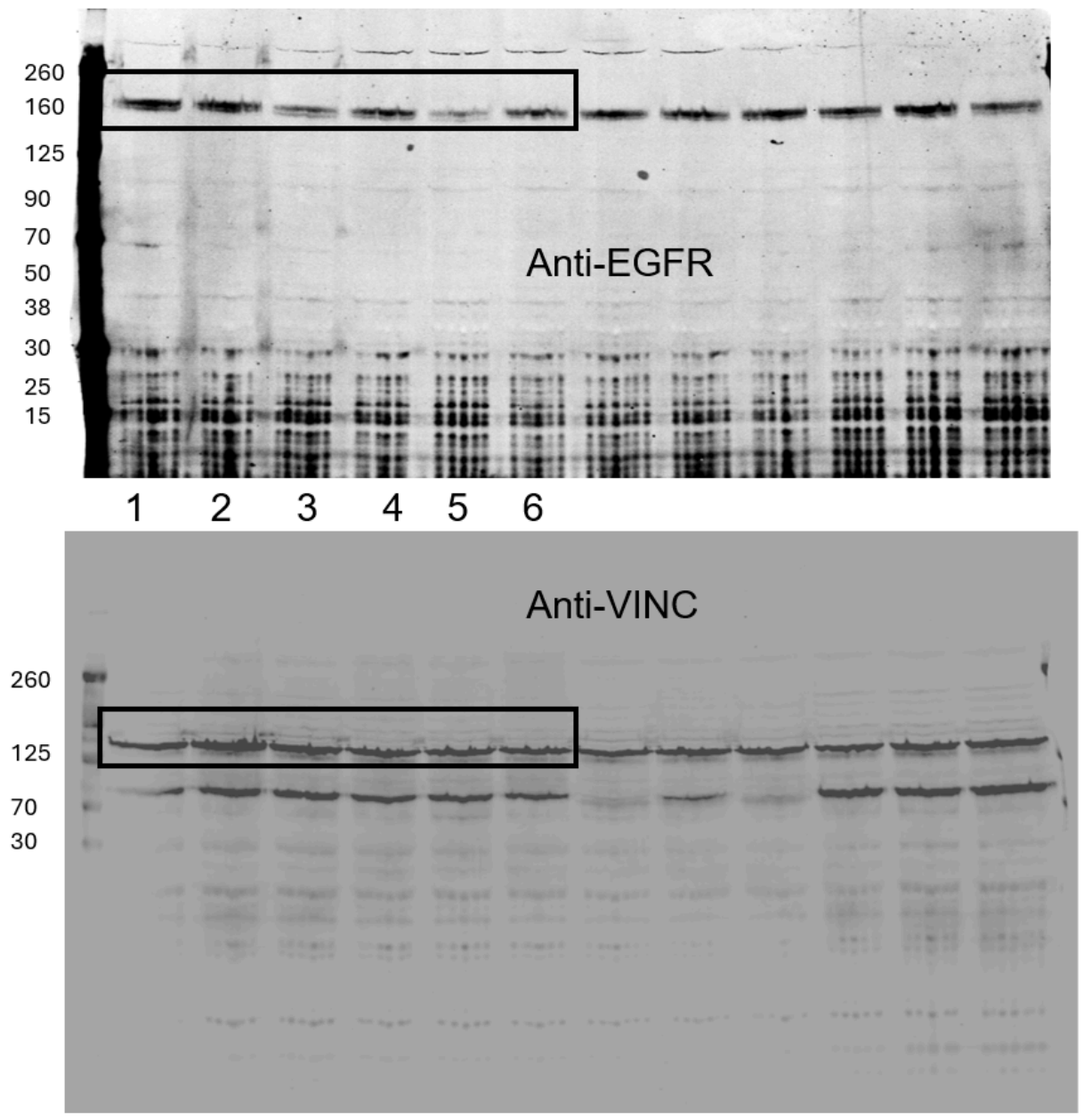

Supplement Figure 10. Full scan for Extended Data Fig. 10

Annotations for each lane:

1:Untreated, 2:EGFRnmb, 3:EGFRn-IGF_EndoTag2, 4:EGFRn-IGF_EndoTagKO

5:EGFRn-Sort_EndoTag, 6:EGFRn-Sort_EndoTagKO

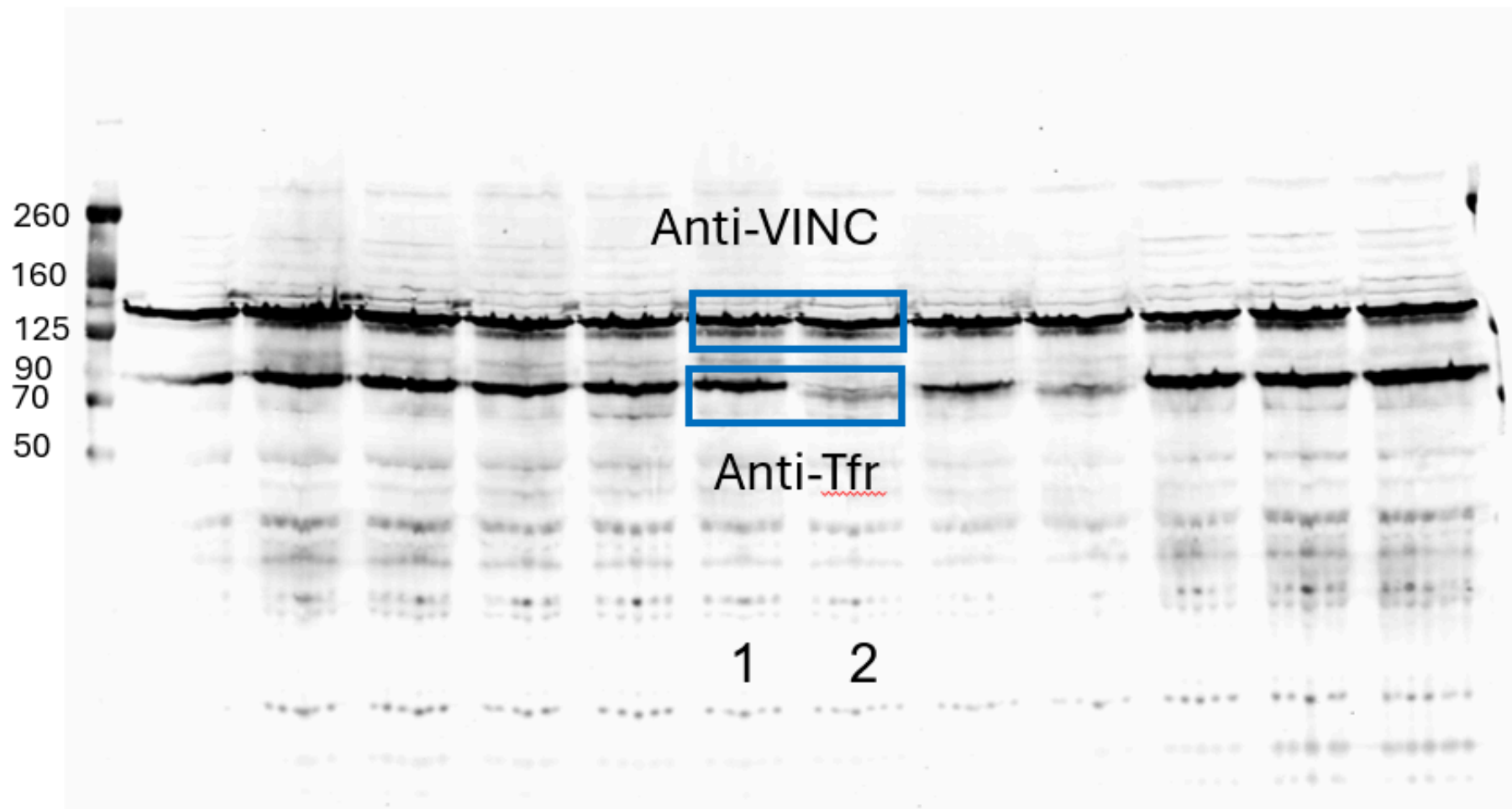

Supplement Figure 11. Full scan for Extended Data Fig. 11(left)

Annotations for each lane:

1:Untreated, 2:TfR KO

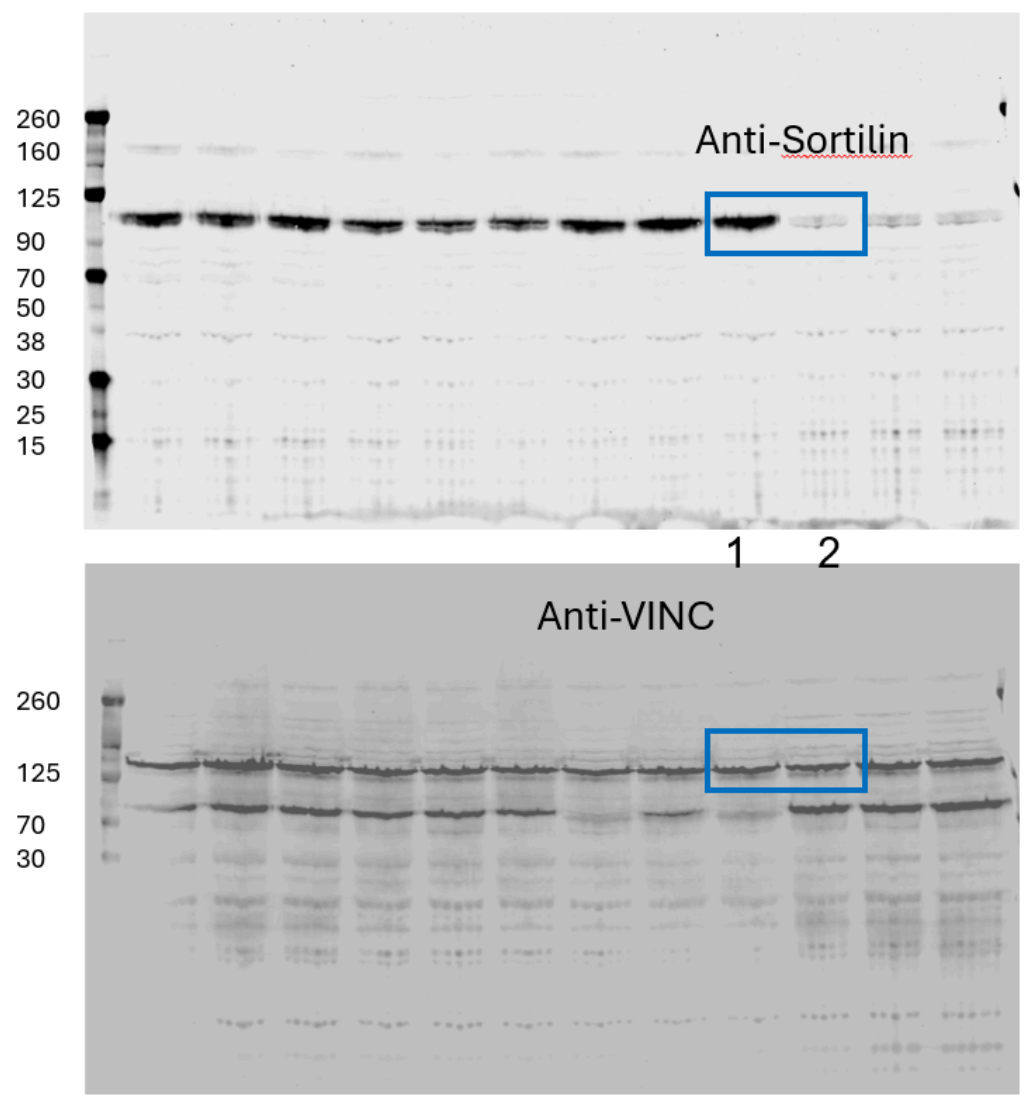

Supplement Figure 12. Full scan for Extended Data Fig. 11(right)

Annotations for each lane:

1:Untreated, 2:Sortilin KO

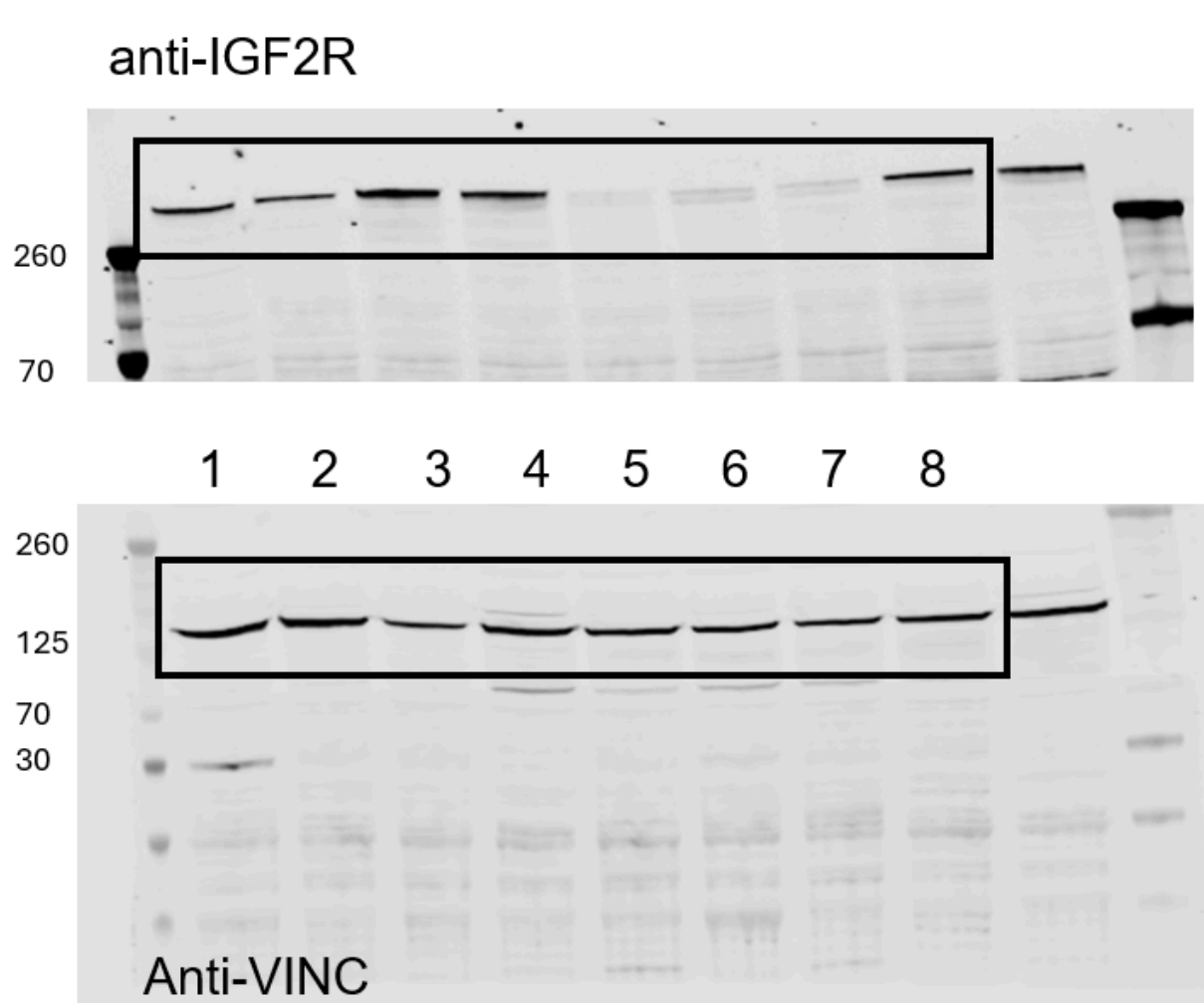

Supplement Figure 13. Full scan for Extended Data Fig. 12a

Annotations for each lane:

1:UMRC2, 2:HeLa, 3:HEPG2, 4:U87MG, 5:A431, 6:A549, 7:NCI H1666, 8:K562

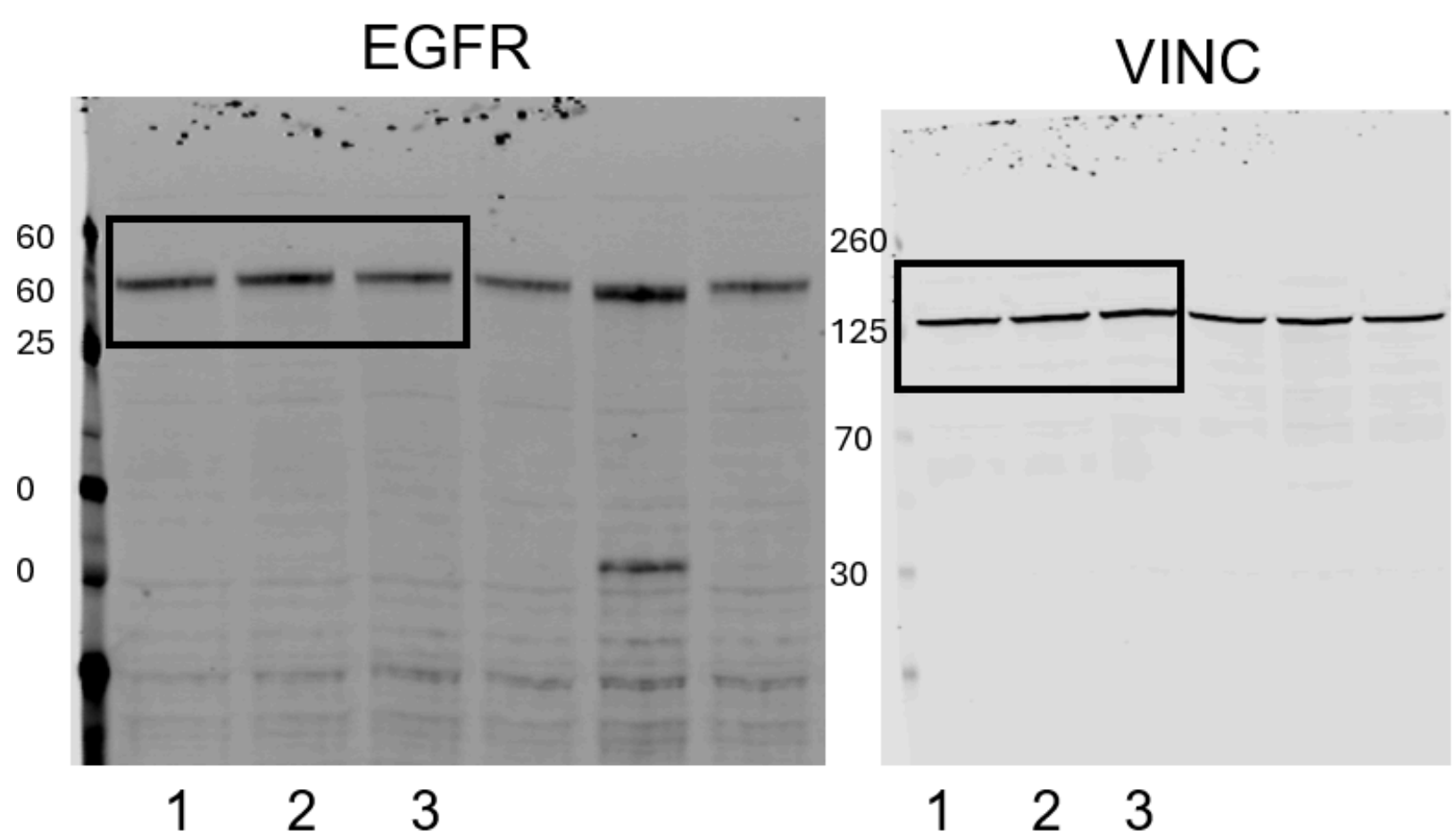

Supplement Figure 14. Full scan for Extended Data Fig. 12b

Annotations for each lane:

1:Untreated, 2:EGFRnmb, 3:EGFRn-IGF_EndoTag1

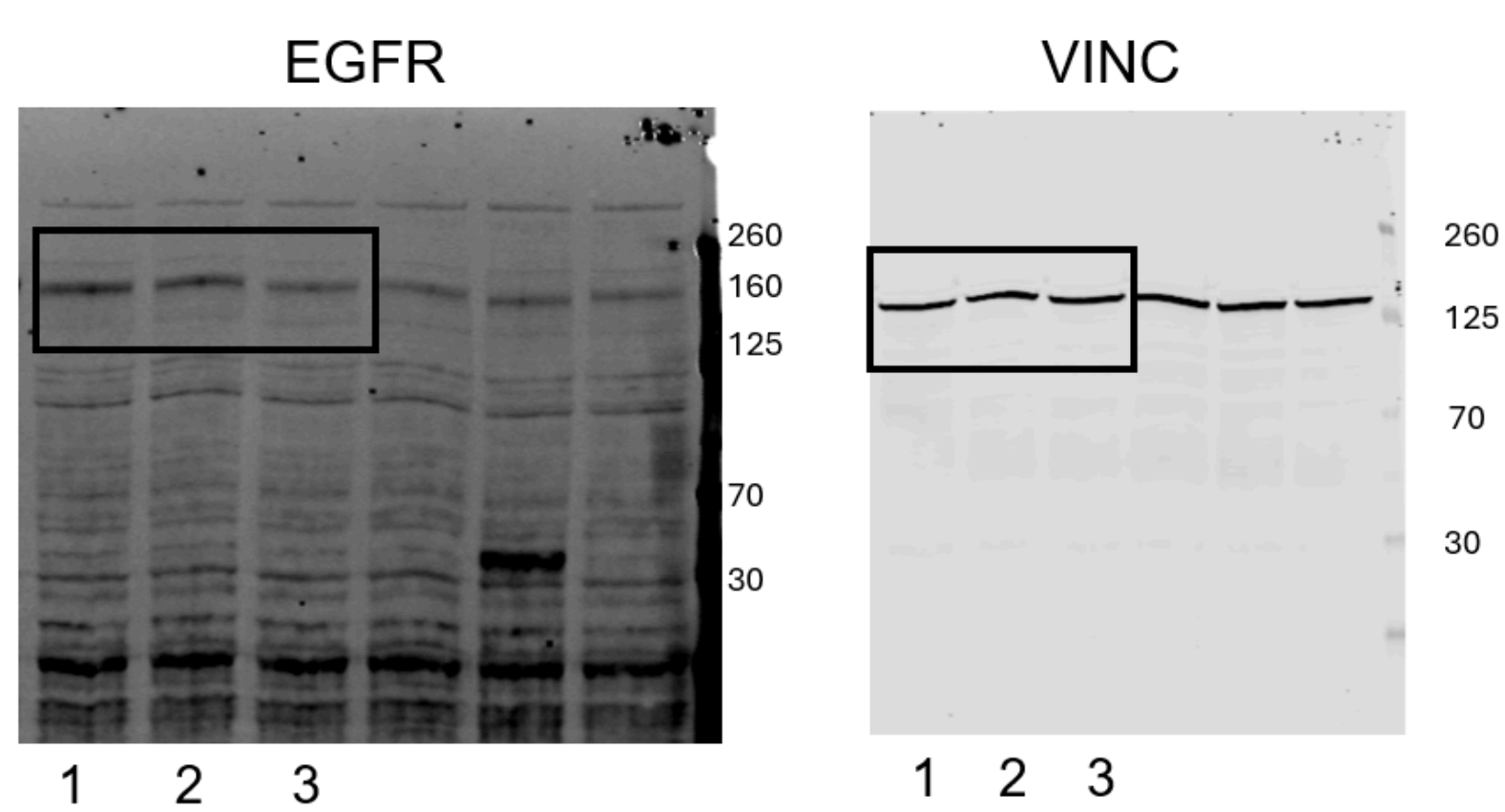

Supplement Figure 15. Full scan for Extended Data Fig. 12c

Annotations for each lane:

1:Untreated, 2:EGFRnmb, 3:EGFRn-IGF_EndoTag1

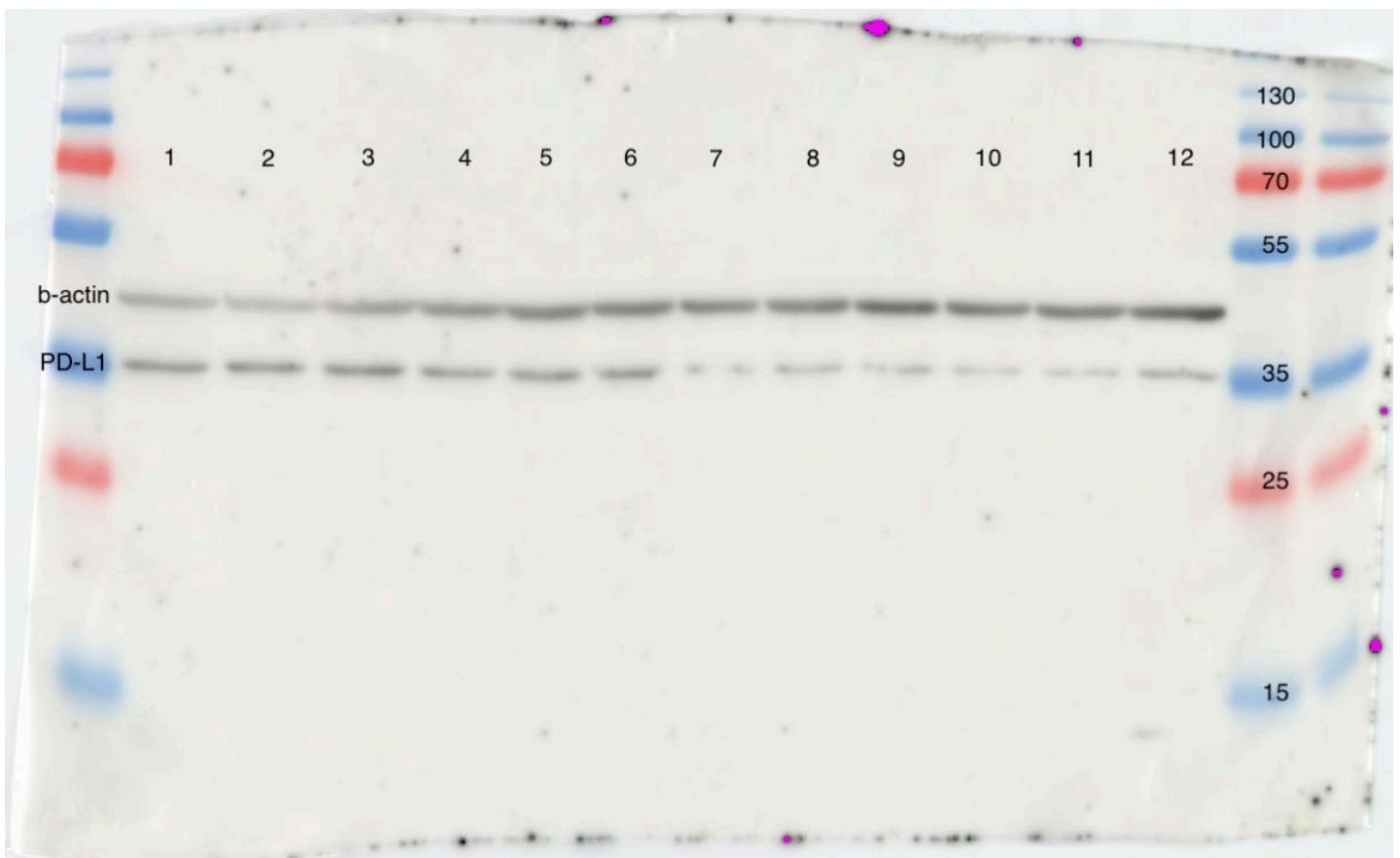

Supplement Figure 16. Full scan for Extended Data Fig. 15

Annotations for each lane: 1-3 different mice from Isotype treatment group; 4-6 different mice from Atz treatment group; 7-9 different mice from Atz-EndoTag3 treatment group; 10-12 different mice from Atz-EndoTag4 treatment group.

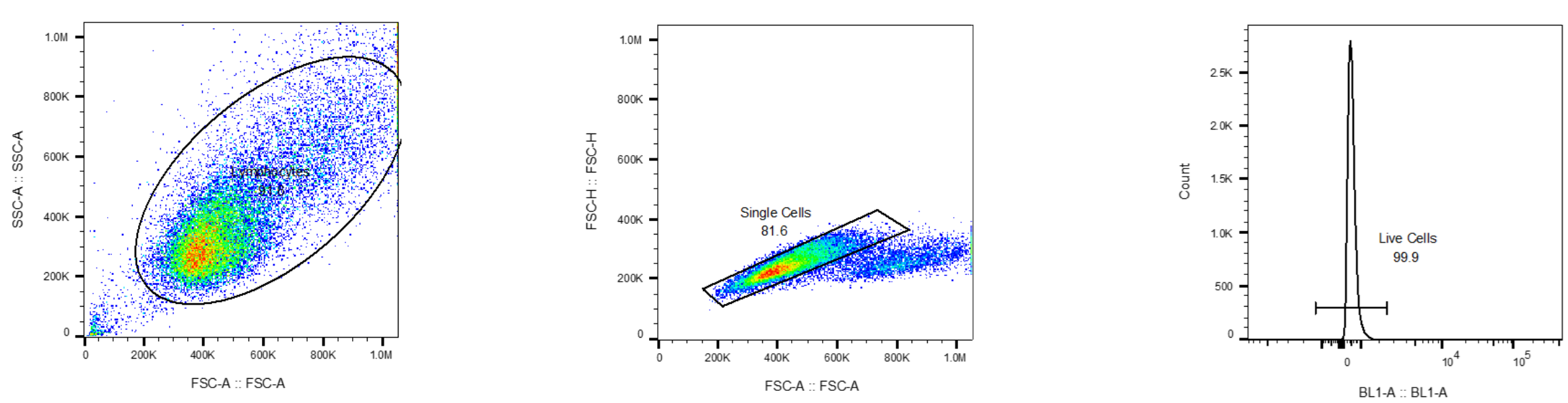

Supplement Figure 17. Flow cytometry gating strategy (representative)

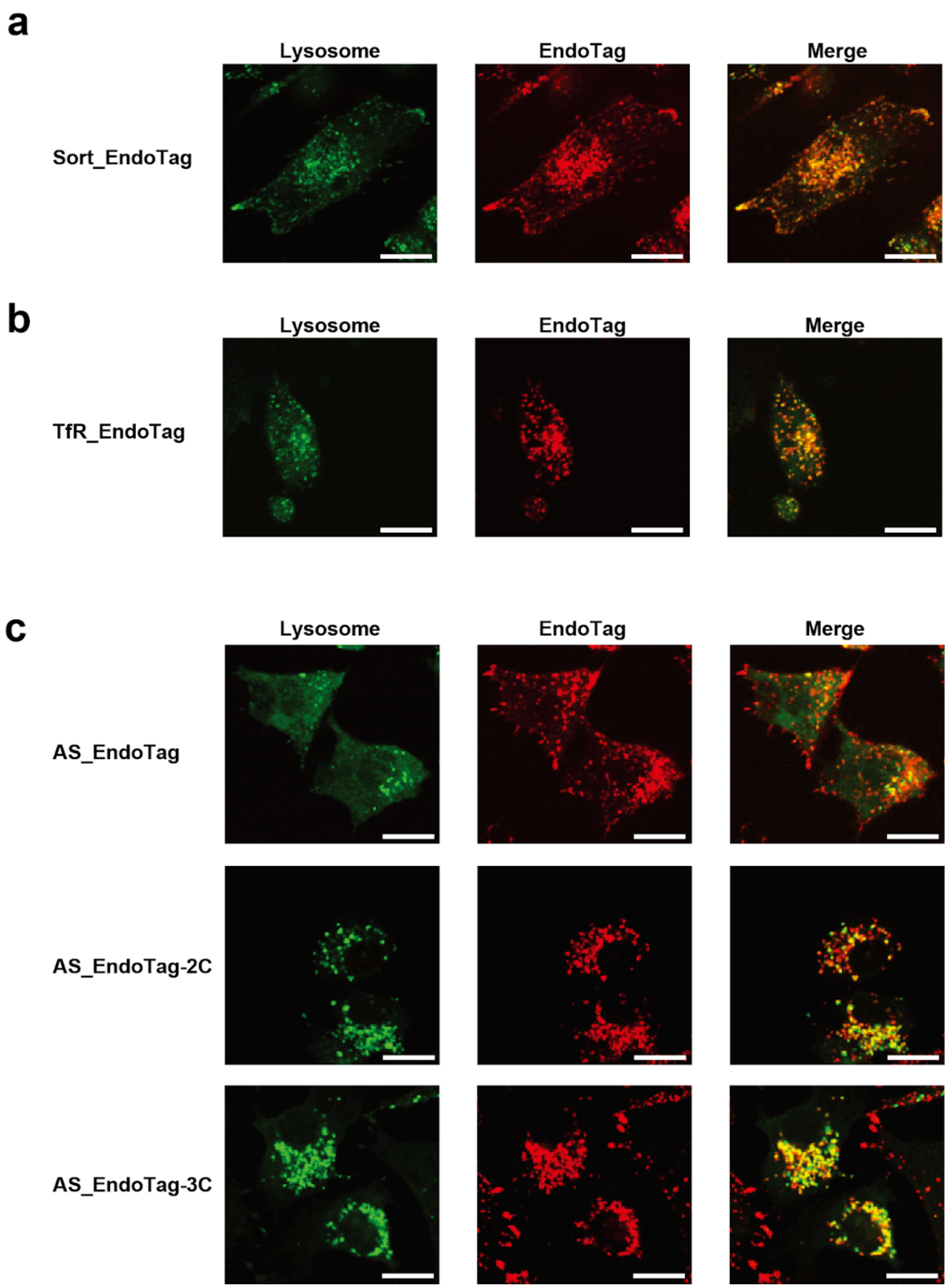

Supplement Figure 18. Confocal imaging of co-localization of EndoTag with lysosome. a, 500nM Sort_EndoTag was incubated with U-251MG cells for 24h. **b**, 500nM TfR_EndoTag was incubated with U-251MG cells for 24h. **c**, 500nM AS_EndoTags were incubated with Hep3B cells for 24h. For **a-c**, the lysosome was labelled by AF488-Lysotracker. All EndoTags were labelled with AF647. Representative images of three replicated samples. The scale bar indicates 20 μm for **a-c**.

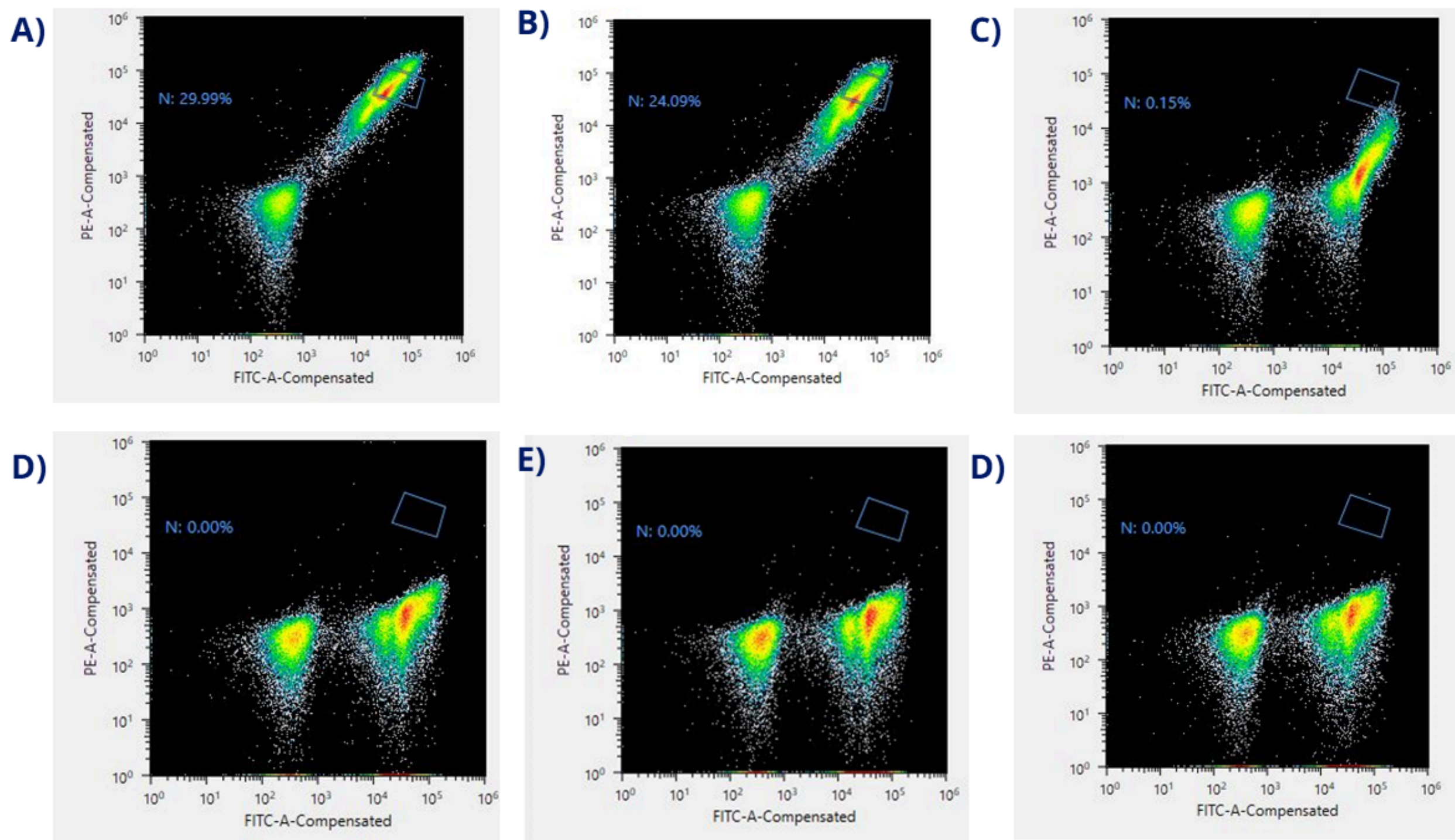

Supplement Figure 19. Epitope verification using N-linked glycan screen of computational designed Sortilin binder Sort_EndoTag using Yeast Display. **a**, 400 nM human ECD SORT-1. **b,** 400 nM mouse ECD SORT-1. **c,** 1000 nM NN-0975. **d,** 1000 nM NN-0979. **e,** 1000 nM NN-0981. **f,** 1000 nM NN-0977.

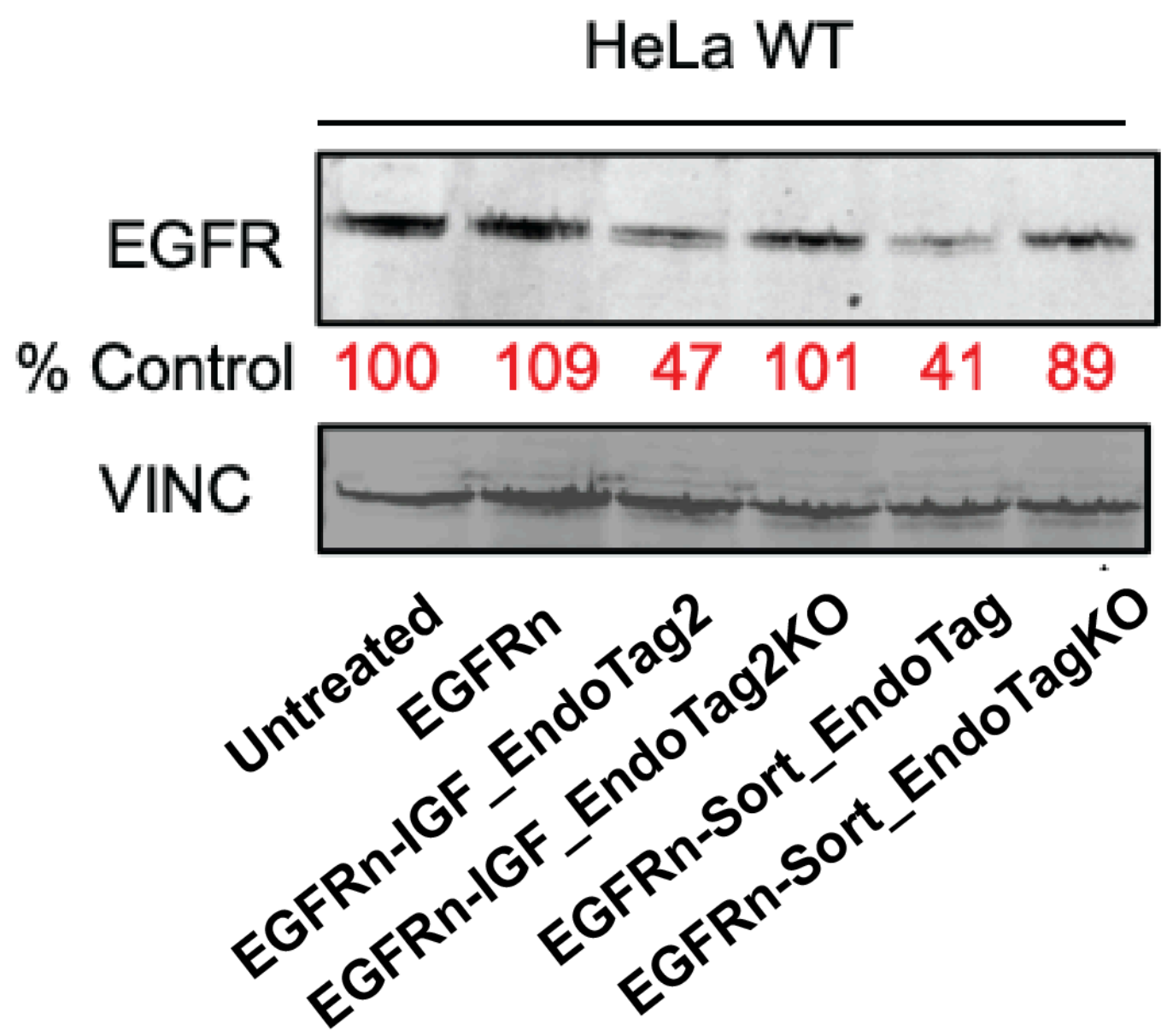

Supplement Figure 20. EGFR degradation with EndoTag interface knockout scaffolds. Western blot analysis of total EGFR levels in Hela WT cells after treatment with 200 nM EGFRn, EGFRn-Sort_EndoTag/EGFRn-Sort_EndoTagKO or EGFRn-IGF_EndoTag2/EGFRn-IGF_EndoTag2KO for 48h. The Western blot plot is representative of three independently replicated samples.

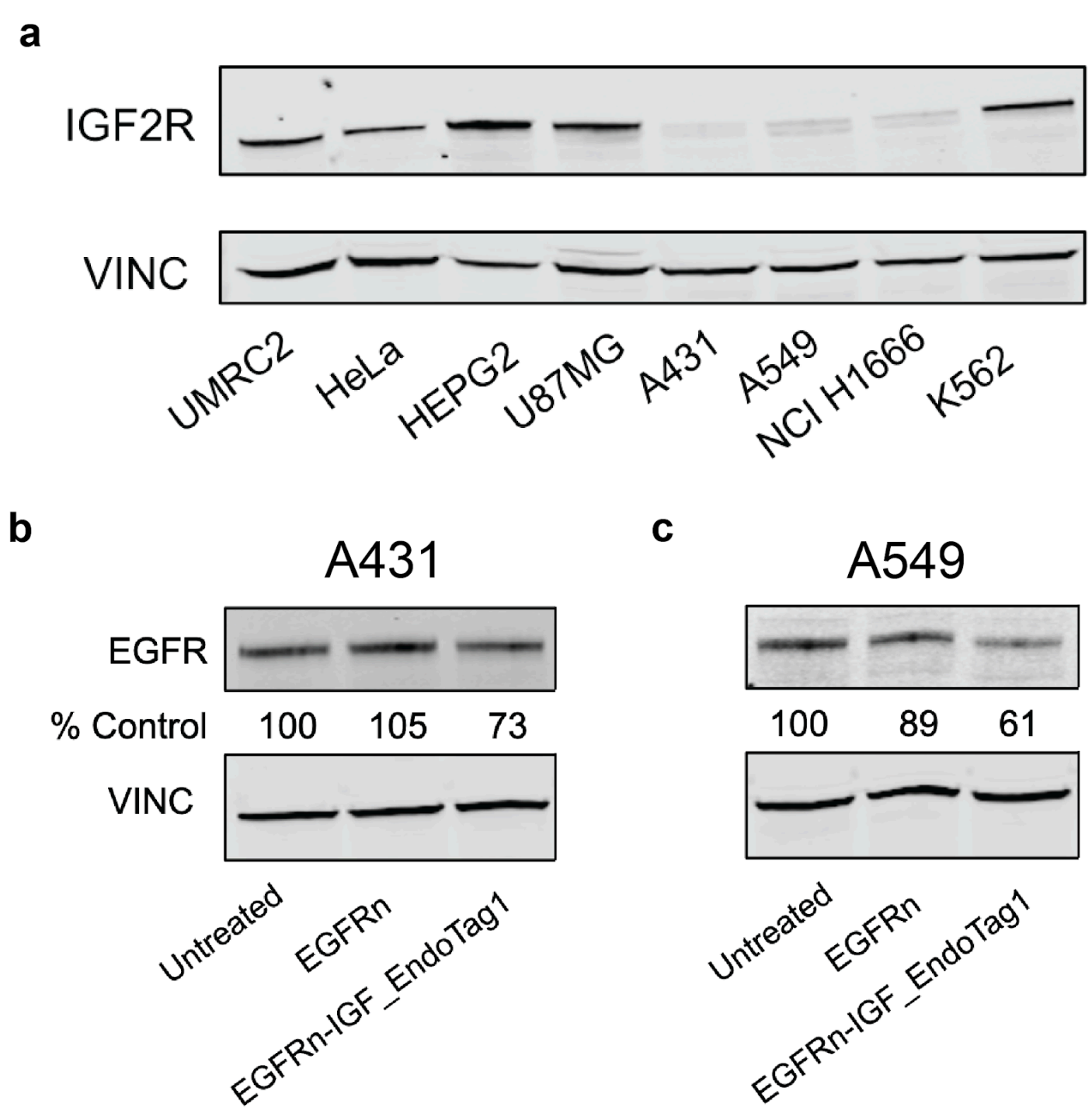

Supplement Figure 21. IGF-2R expression level and degradation capacity across cell lines. **a,** Scanning of IGF-2R level across multiple cancer cell lines. **b,** Western blot analysis of total EGFR levels in A431 WT cells after treatment with 200 nM EGFRn or EGFRn-IGF_EndoTag1 compared to untreated group. **c,** Western blot analysis of total EGFR levels in A549 WT cells after treatment with 200 nM EGFRn or EGFRn-IGF_EndoTag1 compared to untreated group. The Western blot plots are representative of three independently replicated samples.

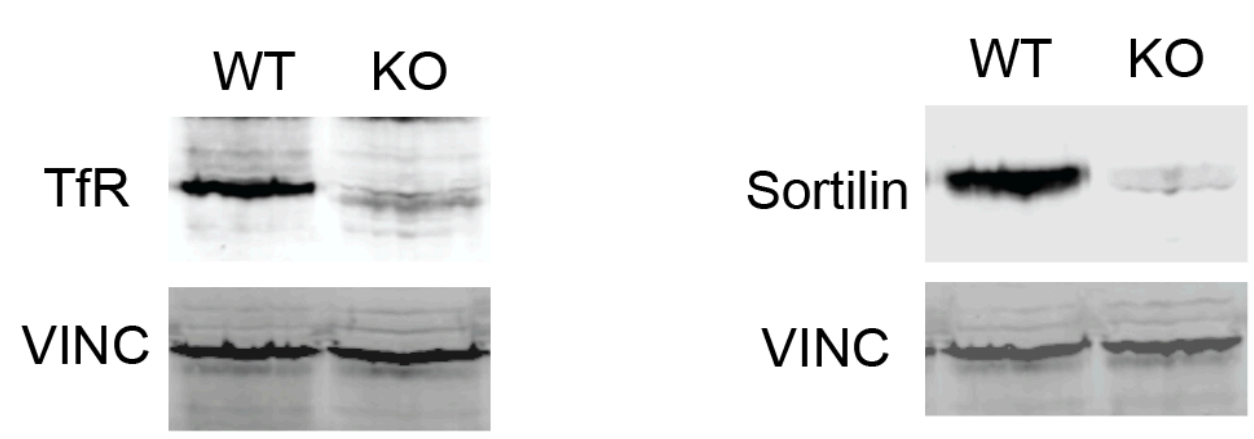

Supplement Figure 22. Validation of knockout of TfR or sortilin in HeLa cells. Western blot analysis of total TfR level or Sortilin in Hela cells with/without knockout of individual gene. The Western blot plots are representative of three independently replicated samples.

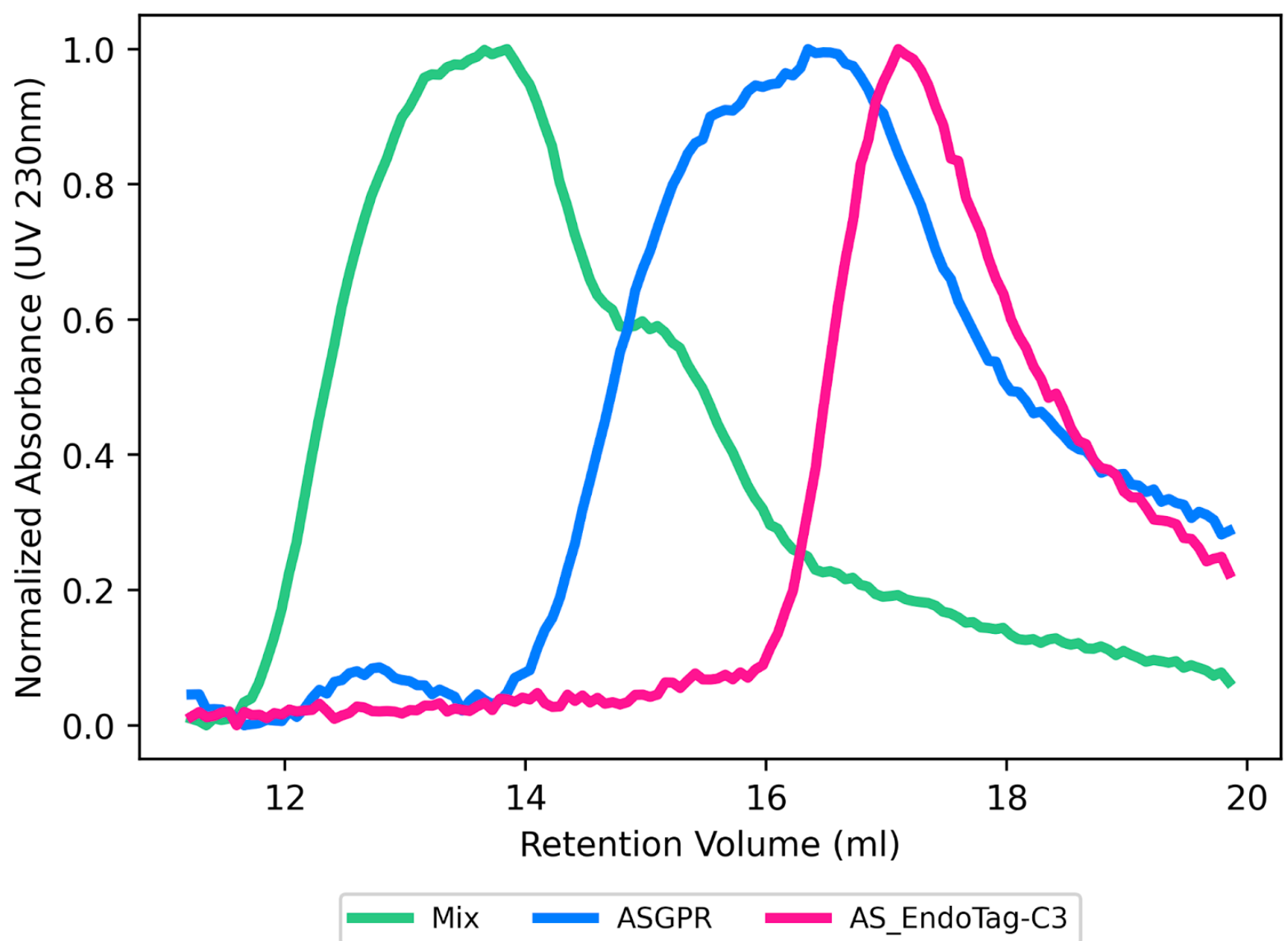

Supplement Figure 23. ASGPR trimer formation mechanism of AS_EndoTag. SEC traces of ASGPR, AS_EndoTag-3C or mixture of AS_EndoTag-3C with ASGPR at 3:1 ratio. ASGPR protein (28.4 kDa) at 1uM concentration was incubated with 3uM AS_EndoTag-3C for 30min and ran through ÄKTA SEC protein purification system using S200 16/90 column.

Supplement Table 1. IGF_EndoTag sequences

ID	Sequence
D6mb	TERRVIQVLEQILEDEDPEVVEKMLEILLEILEEAGDPARKLVEEILRVVRKNLEEARELVRRLS
D11mb	MVDARLLLLIWEAKELLERGNPEEARKVLEEAREIAERNNNEELLKELEVLLEKLE
IGF_EndoTag1	MVDARLLLLIWEAKELLERGNPEEARKVLEEAREIAERNNNEELLKELEVLLEKLEGGSTERRVIQV LEQILEDEDPEVVEKMLEILLEILEEAGDPARKLVEEILRVVRKNLEEARELVRRLS
IGF_EndoTag2	MVEASLWLLIWDAGELVERGNPEEARKVLEEAREIAERNNREEFLKELEVLLEKLEGGSTERRVIQ VLEQILEDEDPEVVEKMLEILLEILEEAGDPARKLVEEILRVVRKNLEEARELVRRLS
IGF_EndoTag3	MEEAQRLLEEWEFQERVDELARKYGERVKEVGRQIIQDPDPEVRRKMLDILERIYREAG
IGF_EndoTag4	MEEAQRLLLEWEAQEWADSQGEDAKRVKQVVQQILQDPDLEVQKKMLEILKRIYEEKG

Supplement Table 2. Sortilin_EndoTag sequences

ID	Sequence
Sort_EndoTag	SFLLKHAVRVIERLLEEGNVELAEKWVEQLKRLAHAQRDPEALRRAEELEEIVEELLE

Supplement Table 3. TfR_EndoTag sequences

ID	Sequence
TfR_EndoTag	KKFIKELLEQGYSKEETAIKVVQKFNVSVVVVTDDEEKAKEIAEYIKKNVPSATVVVYENLIVAKVES HEESTKVWELAQKAY

Supplement Table 4. ASGPR_EndoTag sequences

ID	Sequence
ASmb	AAEEAIRRLKELNTPEAEDLIDFIETLLEKGVPEEEALRQAAALARRWGEPEAARVIEEALKSL
AScombo_1	AAEEAIRRLEELNTPEAEDLIDLIETLLWKGVPEEEALRKAAALTRRWGEPEAARGIEEALKSLS
AScombo_2	AAEEAIRRLEELNTPEAEHILVFIETLLWKGGPEEEALRKAAALVRRWGEPEAARVIEEALKSLS
AScombo_3	AAEEAIRRLEELNTPEAEDLVDLIETLLWKRKPEEEALRKAAALARRWGEPEAARVIEEALKSLS

AScombo_21	AAEEAIRRLKELNTPEAEDLLDFIETLLWKGRPEEEALRKAAALARRWGEPEAARVIEEALKSLS
AScombo_5	AAEEAIRRLNELNTPEAEDLHDFIETLLSKGKPEEEALRKAAALARRWGEPEAARVIEEALKSLS

Supplement Table 5. Highlighted quantitative proteomic analysis of significantly downregulated proteins. H1975 cells were treated with EGFRn or EGFRn-IGF_EndoTag2 for 48h. The proteins were extracted and quantified with proteomic MS. The abundance for each protein was quantified for each treatment group and was normalized to the untreated cells as control group. The normalized relative abundance was shown in log2 scale. P values were calculated by a two-tailed unpaired t-test with Welch's correction. Data are the mean of three biological replicates. Full data is available in the **Source data file 1**.

Index	Gene	ProteinID	log2(EGFRn/Control)	-Log10_pvalue	log2(EGFRn-IGF_EndoTag2/Control)	-Log10_pvalue
RNASET2	RNASET2	O00584	-0.8182627591	0.3759886354	-1.006104591	0.4599565076
RNASEH1	RNASEH1	O60930	-0.8608774694	0.6092568552	-0.8969070696	0.6116116473
EGFR	EGFR	P00533	0.1025100614	0.6324122882	-1.070471242	3.046614618
HMGN2	HMGN2	P05204	-0.9212149029	0.3087685157	-0.8060847699	0.2675414492
SDC1	SDC1	P18827	-0.7058607398	0.2700216279	-0.7678699222	0.2943678634
HBA1	HBA1	P69905	-1.858225974	1.75618735	-0.938129482	0.9732949973
H1-1	H1-1	Q02539	-1.003662595	0.3816470022	-0.9726845489	0.3676827665
ATAD3B	ATAD3B	Q5T9A4	-1.195527326	0.4198468628	-0.8229561787	0.2774920165
MUC16	MUC16	Q8WXI7	-1.530303263	1.880591931	-0.7794132302	0.981993189
BCL7B	BCL7B	Q9BQE9	-0.9979054003	0.8228017875	-0.7908480878	0.6163844182
QRSL1	QRSL1	Q9H0R6	-0.8580319901	1.110368073	-0.8157068092	0.6183245412
CENPH	CENPH	Q9H3R5	-1.046874231	0.6941604232	-0.8080982854	0.5380211362
TMOD2	TMOD2	Q9NZR1	-1.855784561	1.360843603	-0.9724580788	0.7384212447
SNX9	SNX9	Q9Y5X1	-1.061319261	0.3513143796	-1.008354133	0.3311108683
