# Supplementary material for: Designed endocytosis-inducing proteins degrade targets and amplify signals
Source: Nature. 2024 Sep 25;638(8051):796–804. doi: 10.1038/s41586-024-07948-2 (PMC11839401; doi:10.1038/s41586-024-07948-2)
Supplement: Supplementary file 2 [file 41586_2024_7948_MOESM2_ESM.pdf]

nature portfolio

Corresponding author(s): David Baker

Last updated by author(s): 2024/05/07

Reporting Summary

Nature Portfolio wishes to improve the reproducibility of the work that we publish. This form provides structure for consistency and transparency in reporting. For further information on Nature Portfolio policies, see our Editorial Policies and the Editorial Policy Checklist.

Statistics

For all statistical analyses, confirm that the following items are present in the figure legend, table legend, main text, or Methods section.

n/a	Confirmed	
☐	☒	The exact sample size (*n*) for each experimental group/condition, given as a discrete number and unit of measurement
☐	☒	A statement on whether measurements were taken from distinct samples or whether the same sample was measured repeatedly
☐	☒	The statistical test(s) used AND whether they are one- or two-sided<br>*Only common tests should be described solely by name; describe more complex techniques in the Methods section.*
☒	☐	A description of all covariates tested
☒	☐	A description of any assumptions or corrections, such as tests of normality and adjustment for multiple comparisons
☐	☒	A full description of the statistical parameters including central tendency (e.g. means) or other basic estimates (e.g. regression coefficient) AND variation (e.g. standard deviation) or associated estimates of uncertainty (e.g. confidence intervals)
☐	☒	For null hypothesis testing, the test statistic (e.g. *F*, *t*, *r*) with confidence intervals, effect sizes, degrees of freedom and *P* value noted<br>*Give P values as exact values whenever suitable.*
☒	☐	For Bayesian analysis, information on the choice of priors and Markov chain Monte Carlo settings
☒	☐	For hierarchical and complex designs, identification of the appropriate level for tests and full reporting of outcomes
☒	☐	Estimates of effect sizes (e.g. Cohen's *d*, Pearson's *r*), indicating how they were calculated

Our web collection on statistics for biologists contains articles on many of the points above.

Software and code

Policy information about availability of computer code

Data collection	Fluorescence-activated cell sorting was collected in Sony SH800 software suites. Flow cytometry for mammalian cells was collected in Thermo Attune NxT flow cytometer software suites. Binding data was collected in Octet RED96 and processed using Octet Analysis software. Fluorescence plate reading was collected in BioTek Synergy Neo2 Reader. Epifluorescence imaging was conducted on a Yokogawa CSU-X1 microscope equipped with a Hamamatsu ORCA-Fusion scientific CMOS camera and Lumencor Celesta light engine. Confocal image acquisition was controlled via NIS Elements software and data was analyzed via Fiji and custom-written Python Scripts. . The source code for RIF docking is available at https://github.com/rifdock/rifdock. The source code for ProteinInpainting is available at https://github.com/RosettaCommons/RFDesign. The source code for ProteinMPNN is available at https://github.com/dauparas/ProteinMPNN. The scripts used in this paper for binder design applying the above codes, customized image processing and data processing are available in 10.5281/zenodo.11002950.
Data analysis	Data was analyzed and plotted using python3.6 with seaborn0.12.1 and matplotlib3.6.1, and GraphPad Prism9. Flow cytometry data was processed by Flowjo v9. Gels were visualized with an Odyssey CLx Imager (LI-COR) . Image Studio (LI-COR) and ImageJ1.5 was used to quantify gel band intensities.

For manuscripts utilizing custom algorithms or software that are central to the research but not yet described in published literature, software must be made available to editors and reviewers. We strongly encourage code deposition in a community repository (e.g. GitHub). See the Nature Portfolio guidelines for submitting code & software for further information.

Data

Policy information about availability of data

All manuscripts must include a data availability statement. This statement should provide the following information, where applicable:

- Accession codes, unique identifiers, or web links for publicly available datasets
- A description of any restrictions on data availability
- For clinical datasets or third party data, please ensure that the statement adheres to our policy

The structures used to guide the design of EndoTag is available in https://www.rcsb.org/ (IGF-2R: PDB 6UM2; ASGPR: 6YAU, 5JQ1; Sortilin: 3F6K). The raw data for animal data and mass spectrum is in Source Data in this manuscript. The full scan for the Western Blot gels are available in the Supplement Information. The raw data for the flow cytometry, next generation sequencing, designed binder models and sequences are available in 10.5281/zenodo.11002950.

Research involving human participants, their data, or biological material

Policy information about studies with human participants or human data. See also policy information about sex, gender (identity/presentation), and sexual orientation and race, ethnicity and racism.

Reporting on sex and gender	N/A
Reporting on race, ethnicity, or other socially relevant groupings	N/A
Population characteristics	N/A
Recruitment	N/A
Ethics oversight	N/A

Note that full information on the approval of the study protocol must also be provided in the manuscript.

Field-specific reporting

Please select the one below that is the best fit for your research. If you are not sure, read the appropriate sections before making your selection.

☒ Life sciences ☐ Behavioural & social sciences ☐ Ecological, evolutionary & environmental sciences

For a reference copy of the document with all sections, see nature.com/documents/nr-reporting-summary-flat.pdf

Life sciences study design

All studies must disclose on these points even when the disclosure is negative.

Sample size	No statistical analysis was used to determine the sample size. The number of sample size is determined by our ability to detect meaningful differences between treatments. Western blot experiments in vitro and BLI binding assays were conducted with sample size 1 based on low variance from our previous experience. Western blot experiments in mice were performed with sample size 3 to reduce the variance of protein level across animals from our previous best practice .
Data exclusions	No data were excluded from this study
Replication	The data were collected as biological replicates as indicated in the figure legend. All cell experiences were done multiple times to ensure reproducibility. All images were representative of three independently replicated samples.
Randomization	For the animal study, the mice been randomly assigned into the different treatment group.
Blinding	Researchers were not blinded in this study. No additional blinding was used for Western Blot analysis or flow cytometry analysis to compare specific treatments.

Reporting for specific materials, systems and methods

We require information from authors about some types of materials, experimental systems and methods used in many studies. Here, indicate whether each material, system or method listed is relevant to your study. If you are not sure if a list item applies to your research, read the appropriate section before selecting a response.

Materials & experimental systems

n/a	Involved in the study	
☐	☒	Antibodies
☐	☒	Eukaryotic cell lines
☒	☐	Palaeontology and archaeology
☐	☒	Animals and other organisms
☒	☐	Clinical data
☒	☐	Dual use research of concern
☒	☐	Plants

Methods

n/a	Involved in the study	
☒	☐	ChIP-seq
☐	☒	Flow cytometry
☒	☐	MRI-based neuroimaging

Antibodies

Antibodies used

Alexa Fluor® 647 anti-human EGFR Antibody (Biolegend, 352918), PE anti-human CD222 (IGF2R) Recombinant Antibody (Biolegend, 364204), EGFR Monoclonal Antibody (clone 199.12, Invitrogen, AHR5072), IGF2R Antibody Alexa647 (clone 2G11, Novus, NB300-514AF647), Human PD-L1 Alexa Fluor® 647-conjugated Antibody (Rndsystems, FAB1562R), Human IgG1 Isotype Control Alexa 647 (Novus, DDXCH01A647), rabbit anti-EGFR D38B1 Cell Signaling Technologies (#4267) , rabbit anti-HER2 2242 Cell Signaling Technologies (2242), rabbit anti-PD-L1 E1L3N Cell Signaling Technologies (13684), mouse anti- vinculin V284 Bio-Rad (MCA465GA). Anti-LAMP2A antibody (Abcam ab18528), goat anti-rabbit- IgG Alexa Fluor™ 488 secondary antibody (Thermo Fisher A-11034), anti PD-L1 (sc-518027), anti beta-actin (sc-47778), goat anti-mouse IgG H&L (HRP) (Abcam, ab205719); 800CW goat-anti-mouse or goat-anti-rabbit (LI-COR 926-32211), rabbit anti-CTLA4 E1V6T Cell Signaling Technologies (96399)

Validation

Alexa Fluor® 647 anti-human EGFR Antibody (Biolegend, 352918): Verified Reactivity to Human, FC - Quality tested
PE anti-human CD222 (IGF2R) Recombinant Antibody (Biolegend, 364204): Verified Reactivity to Human, ICFC, FC - Quality tested
EGFR Monoclonal Antibody (clone 199.12, Invitrogen, AHR5072): Target Species: Human, applications for Immunocytochemistry, Immunofluorescence, Immunoprecipitation, Western Blot
IGF2R Antibody Alexa647 (clone 2G11, Novus, NB300-514AF647): Reactivity Hu, Mu, Rt, Bv, Pm; Applications WB, ELISA, Flow, ICC/IF, IHC, IP, CyTOF-ready
Human PD-L1 Alexa Fluor® 647-conjugated Antibody (Rndsystems, FAB1562R): Species Reactivity Human, Detects human PD-L1/B7-H1 in direct ELISAs. Applications for FC verified.
Human IgG1 Isotype Control Alexa 647 (Novus, DDXCH01A647): Reactivity to human. Applications for FC
rabbit anti-EGFR D38B1 Cell Signaling Technologies #4267: REACTIVITY H M Mk; Applications to WB verified
rabbit anti-HER2 2242 Cell Signaling Technologies (2242): REACTIVITY H; Applications to WB verified
rabbit anti-PD-L1 E1L3N Cell Signaling Technologies (13684): REACTIVITY H; Applications to WB verified
rabbit anti-CTLA4 E1V6T Cell Signaling Technologies (96399): REACTIVITY H; Applications to WB verified
mouse anti- vinculin V284 Bio-Rad (MCA465GA): Target Species Human; Applications verified for FC/WB/IF
Anti-LAMP2A antibody (Abcam ab18528): Reacts with: Mouse, Human; Suitable for: WB, ICC/IF, IHC-P
anti PD-L1 (sc-518027): specific for an epitope mapping between amino acids 221-240 near the C-terminus of Pdcd-1L1 of human origin; verified application for WB/FC
anti beta-actin (sc-47778): beta Actin Antibody (C4) is recommended for detection of β-Actin of mouse, rat, human, avian, bovine, canine, porcine, rabbit, Dictyostelium discoideum and Physarum polycephalum origin by WB, IP, IF, IHC(P) and ELISA

Eukaryotic cell lines

Policy information about cell lines and Sex and Gender in Research

Cell line source(s)

MDA-MB-231 (ATCC HTB-26), Jurkat (ATCC TIB-152), K562 (ATCC CCL-243), Hela (ATCC CCL-2), H1975 (ATCC CRL-5908), Hep3B (ATCC HB-8064) from the American Type Culture Collection (ATCC). U251-MG (Sigma-Aldrich, 09063001). A20 (ATCC, #TIB-208). Expi293F™ Cells (ThermoFisher A14527) . Hela IGF-2R KO cell was a kind gift from Steven Banik Lab at Stanford University.

Authentication

Authentication was provided by ATCC. No additional authentication.

Mycoplasma contamination

The cell lines were not tested for mycoplasma contamination

Commonly misidentified lines
(See ICLAC register)

There are no misidentified lines.

Animals and other research organisms

Policy information about studies involving animals; ARRIVE guidelines recommended for reporting animal research, and Sex and Gender in Research

Laboratory animals

8-week-old female BALB/c mice (purchased from Charles River) were used in this study

Wild animals

No wild animals are included in this study

Reporting on sex	All female
Field-collected samples	For survival monitor, each mouse was sacrificed respectively when tumours reached 1,000 mm3. The light/dark cycle was 14 h light/10 h dark (lights on at 07:00; lights off at 21:00). The temperature was 20-24 °C and the relative humidity was 55 ± 10%, with controlled supply of High Efficiency-Particulate Air (HEPA) filtered air provided to individually ventilated cages. Maximum number of animals per cage was 5. Social isolation was avoided whenever possible. The type of food was autoclaved diet pellets RM3A (P), from SDS Special Diets Services (Product code: 801030). Food was placed in a grid inside the cage and provided ad libitum to animals. The type of water was sterile water treated by reverse osmosis. Water was provided ad libitum to animals through bottles with a capillary hole
Ethics oversight	All animal experiments were conducted at the Instituto de Medicina Molecular João Lobo Antunes (IMM, Lisbon). Animal work was performed in strict accordance with Portuguese Law (Portaria 1005/92) and the European Guideline 86/609/EEC and follow the Federation of European Laboratory Animal Science Associations guidelines and recommendations concerning laboratory animal welfare. All animal experiments were approved by the Portuguese official veterinary department for welfare licensing – Direção Geral de Alimentação e Veterinária (DGAV) and the IMM Animal Ethics Committee (authorization AWB_2021_03_GB_Targ CancerDrugs)

Note that full information on the approval of the study protocol must also be provided in the manuscript.

Plants

Seed stocks	N/A
Novel plant genotypes	N/A
Authentication	N/A

Flow Cytometry

Plots

Confirm that:

- [x] The axis labels state the marker and fluorochrome used (e.g. CD4-FITC).
- [x] The axis scales are clearly visible. Include numbers along axes only for bottom left plot of group (a 'group' is an analysis of identical markers).
- [x] All plots are contour plots with outliers or pseudocolor plots.
- [x] A numerical value for number of cells or percentage (with statistics) is provided.

Methodology

Sample preparation	For Yeast display: DNAs encoding the minbinder sequences were transformed into Saccharomyces cerevisiae EBY100 strain. The yeast cells were grown in CTUG medium and induced in SGCAA medium. After washing with PBSF (PBS+1% BSA), the cells were incubated with 1uM biotinylated target proteins (IGF-2R, ASGPR, Sortilin) together with streptavidin–phycoerythrin (SAPE, ThermoFisher, 1:100) and anti-c-Myc fluorescein isothiocyanate (FITC, Miltenyi Biotech, 6.8:100) for 30min. After washing twice with PBSF, the yeast cells were then resuspended in PBSF and screened via FACS<br><br>For mammalian cell internalization / surface binding assay, for cellular uptake assays using suspension cell lines (K-562, Jurkat), the cells were incubated with corresponding fluorescence-labeled protein constructs at 37 °C for indicated time, then spun down at 500g for 5min, resuspended and washed with cold PBS. After three washes, the cells were resuspended and transferred to a 96-well plate. For cellular uptake assays using adherent cell lines (U-251MG, Hep3B, Hela, H1975), the cells were incubated with corresponding fluorescence-labeled protein constructs at 37 °C for indicated time, then washed with cold PBS for three times. The cells were then treated with 50uL of trypsin and incubated at 37 °C for 10 min followed by adding 50uL of DMEM media. The resuspended cells were then transferred to a 96-well plate followed by two PBS washes. Flow cytometry was then performed in Attune NxT flow cytometer (Thermo Fisher). The data was analyzed in FlowJo software.<br><br>For cell surface receptor degradation experiments, the cells were first incubated with corresponding protein reagents for indicated time at 37 °C, then washed with cold PBS three times. For suspension cell lines, the cells were resuspended and transferred to the 96-well plate; for adherent cell lines, the cells were first treated with trypsin for 10 minutes then transferred to the 96-well plate. The cells were then stained with corresponding fluorescence-labeled antibodies against the corresponding receptor for 1 hour at room temperature. After washing three times with cold PBS for flow cytometry, flow cytometry was performed in Attune NxT flow cytometer (Thermo Fisher). The data was analyzed in FlowJo software.
Instrument	Sony SH800 / Thermo Attune NxT

Software	Sony SH800S software / Sony Attune NxT software / FlowJo v9
Cell population abundance	For Yeast display: At least 10,000 yeast cells were collected in each sorted fraction. The cells were sorted using the purity mode in Sony SH800 to achieve maximized purity. The purity was automatically calculated by the machine.<br><br>For mammalian cell internalization / surface binding assay, at least 2,000 mammalian cells were collected and resuspended in flow focusing buffer per sample.
Gating strategy	For Yeast display: The gate was selected based on the distribution of the main population of double positive PE and FITC signals (>10^4 & <10^5). The gate was consistently identical across all group tested for the same target.<br><br>For mammalian cell internalization / surface binding assay, the cells were gated based on the major population based on SSC and FSC.

☒ Tick this box to confirm that a figure exemplifying the gating strategy is provided in the Supplementary Information.
