# Supplementary material for: Designed endocytosis-inducing proteins degrade targets and amplify signals
Source: Nature. 2024 Sep 25;638(8051):796–804. doi: 10.1038/s41586-024-07948-2 (PMC11839401; doi:10.1038/s41586-024-07948-2)
Supplement: Supplementary file 3 [file 41586_2024_7948_MOESM3_ESM.pdf]

Peer Review File

Manuscript Title: Designed Endocytosis Inducing Proteins Degrade Targets and Amplify Signals

Reviewer Comments & Author Rebuttals

Parts of this Peer Review File have been redacted as indicated to maintain the confidentiality of unpublished data

Reviewer Reports on the Initial Version:

Referees' comments:
attach and degrade soluble or membrane targets of interest, EndoTags. The authors also report a logic gated strategy that further increases specificity of the degrader and cell signaling activated by the tag in a synthetic notch system. The work utilizes an impressive suite of de novo design approaches previously pioneered by the Baker lab to find de novo helical bundles that bind in both ligand and non-ligand sites. It directs binders to two well-established recycling receptors, ASGPR and IL-2R/CI-M6PR and two others, the transferrin receptor and sortillin. They show similar levels of degradation as seen by others using similar targets (PD-L1, EGFR) as well as some new ones. The work is very well done, and they show an impressive number of designs and applications.

There are, however, several aspects that reduce the impact of the work especially for a journal like Nature that focuses on paradigm shifting research. There have been over the past few years a number of extracellular degrader platforms that have been described, including the two acknowledged such as LYTACs, and KineTACs. It is not clear what significantly distinguishes the EndoTags that would constitute a breakthough. The authors make the theoretical argument that other platforms like LYTACs and KineTACs, have depended on endogenous ligand-receptor interactions which could have several drawbacks in that they could be affected by competition with endogenous ligand and triggering of unwanted biology. However, the authors do not show this is a convincing advantage for EndoTags. They argue that EndoTags are genetically encoded which has advantages to the LYTAC platform which uses glycan conjugation. But other platforms such as KineTACs are also fully genetically encoded. EndoTags are fully non-natural which could trigger immunogenicity issues if used therapeutically as they suggest. Moreover the design platform is very unique to Baker group and is non-trivial to apply. Thus, it is not clear why other investigators would adopt EndoTags over traditional antibodies or single domain binders, scaffolds which are validated clinically, and use technologies for finding binders that is far simpler and more accessible. It also depends on having a structure of the protein target but this is less of a barrier these days with good predictive modeling methods. While the work is impressive in scope, in our view it does not really cut a new path that many investigators will follow either from a technical or biological perspective. Thus, we would recommend it be published in a high quality but more specialized journal such as Nature Biotechnology or Nature Methods. Below are additional comments that could improve the paper.

Additional Comments:

1. The authors argue that the EndoTag system is bio-orthogonal by desinging binders do not bind where the natural ligands bind. In some cases, biorthogonality is obviously not achieved – the IGF-2R EndoTag would surely compete with natural ligand binding based on the structures in Fig 1. Even if the sites do not overlap, it is still possible the EndoTags affect the dynamics or have allosteric effects on the degrader that does impact its natural function. The authors have not tested the natural ligand functional effects from what I see here. For example, does the TfR EndoTag impact transferrin uptake. These are relatively simple things to test and important to add if they claim these will not impact native function. Moreover, even if shown to be benign, that isn't enough to say EndoTags have advantages to other systems that bind the natural ligand site unless they compare them head-to-head. Abundant recycling systems like the TfR can be far more abundant than that needed to degrade the protein of interest, so whether one binds at the ligand binding site or not it doesn't matter.
2. Are the EndoTags recycled or are they degraded? Are these functionally really better than simple non-degrading binders? Virtually all the experiments are degradation and trafficking experiments, with no functional readout. It is important to show a functional benefit.

Minor comments/questions:

1. Sometimes the exact lysosomal marker is not defined for confocal images. Please specify.
2. Line 90 – what are "all 4 receptor systems"?
3. Line 110 – Neurotrophins are a broad class. Do they all bind to the same site?
4. Line 122 - Naming conventions – What is the difference between sortmb and Sort_EndoTag?
5. Line 125 – Extensive washing is great but doesn't completely rule out surface binding, are there any controls to negate this (e.g. internalization at 4C with washing step)?
6. Line 174 – We would have liked to see monomer versions of D6 and D11 binders to compare. It's possible that making dimers out of single domain binders simply ablates all binding to the receptor.
7. Line 259 – There is no figure 1k
8. Line 269 – Ideally more brain relevant cell lines would be used to show that TfR and Sort tags could work in the brain.
9. Figure 2 – an EndoTag alone control would be good to see to ensure the tag isn't affecting anything.
10. The MS data is a bit underexplained in my view. What is the control? What are other downregulated hits? Do these support the position that the tags are biorthogonal?
11. Overall, many figures have "control" listed as one of the conditions. It is not always clear what this control is. Is this an isotype control or simply vehicle treated, etc?
12. Line 327 – Fold change over what?
13. Figures 3f&g would be nice to see colocalization with a merge
14. In the case of AND gated degraders, it seems from the diagram alone that the gating receptor would also get degraded. Is this happening? This could be a problem if undetected.
15. For cell signaling activation of the Ortho-SNIPR, why was LHDA dimer used as the comparison? How was the linker chosen for this dimer?
16. Line 418 – not sure if the data entirely supports this without a competition experiment. It is true they were designed this way and match the epitope by N-glycan attachment.
17. Line 431 – One limitation is that a structure or structural model of the receptor- native ligand

complex is required to design an EndoTag that does not sterically occlude ligand binding. This is a higher bar than having a good model of the receptor alone. The authors should discuss this

18. Line 442 – This is a great point, but really makes one ask why a synthetic system was used to show signal activation. Natural receptor activation is much more relevant and impactful.

Referee #2 (Remarks to the Author):

Designed Endocytosis-Triggering Proteins mediate Targeted Degradation and amplify signaling

Prior work published in Nature from the Baker and the Bertozzi labs respectively established de novo protein binders design with Rosetta (Nature 2022) and chimeric antibody-based degraders (LYTACs) for degradation of extracellular and transmembrane proteins via endocytosis-lysosomal pathways (Nature 2020). Here, Bertozzi and Baker have teamed up to develop a chimeric protein based strategy to induce endocytosis of extracellular and transmembrane proteins – an approach they here call EndoTags. The design EndoTag as binding proteins for 5 different receptors triggering endocytosis via various mechanisms, and then go on to conjugate those to transmembrane proteins (EGFR, PD-L1) as well as soluble proteins, showing their endocytosis-driven degradation. They also show utility of their approach for a RNA-based delivered secretory approach, and triggering of transcription factor based signaling upon target degradation.

There are many positives to this article. The work is of extremely high quality and reach, as expected by these two laboratories, offering an enticing proof of concept for bringing Rosetta-based protein design towards applications for targeted protein degradation. This could offer advantages relative to the antibody-based approach currently explore via LYTAC – however a limitation of the study is that in the few experiments where a side-by-side comparison of an EndoTag-based versus conventional LYTAC (antibody) based degraders the improvement in performance appears minimal, so such advantages are not strictly established. Other positives are the wide-range of receptors, proteins and mechanisms targeted in the study which evidence scope and power of the approach. Another negative is the therapeutic potential and appropriateness to drug development are not demonstrated; for example, do these agents work in vivo? Could they exhibit improved PK properties over e.g. conventional LYTACs which suffer from the antibody-based scaffold? Overall, the paper is nonetheless exciting and could eventually be published in a high-impact journal as a significant advance in the field.

In addition to the limitations highlighted above, to strengthen and improve the paper, the authors might want to consider including “negative control” non-binding EndoTags, ideally single-point or multiple mutants of otherwise identical fold, to evidence specificity – much in the same way as non-binding ligands for the E3 ligase VHL/CRBN are widely used in the PROTAC field as they lead to non-degrading molecules of otherwise very similar physico-chemical properties. It would also be interesting to evidence the kinetics of protein degradation – e.g. is the target depleted in minutes, hours, days? I notice in many cases the blots are from cells treated for up to 48 h.

Referee #3 (Remarks to the Author):

In this manuscript, Huang et al. present a protein design strategy to exploit the endocytic cell machinery to ultimately affect membrane receptor function and cellular signaling.
The authors have to be commended for putting together a manuscript that is well conceived, use state of the art protein design and innovative approaches to drive receptor internalization. This strategy, as the authors claim, was designed with the goal to differentiate from other approaches by being generalizable, not interfering with the native signaling of the internalizing receptors, by allowing tissue specific activity, and by enabling logic-gated activity. It is fair to say, however, that there have been a good handful of papers that have explored bispecific molecules to drive internalization and lysosomal degradation including the LyTACs, the KINETACs, PROTABs but also de Goeij et al Mol Cell Ther 2016, Andreev et al. Mol Cell Ther 2017, and Devay et al. Bioconj Chem 2017 who all did it to improve ADC activity.

Although the framework presented here has the potential to improve on the limitations of other strategies, little data is actually presented to demonstrate in fact that it does. Without further evidence, I do not think this essential claim can be made.
Major comments are listed below:
1) Bypass of native ligand: While the described peptides do bind at sites on the receptors that are orthogonal to ligand binding sites, there is little data validating that the normal signaling pathways are not impacted (either by less receptor on the cell surface, or by directly inducing signaling).
2) Tissue selectivity: Many other cell surface degradation studies have demonstrated in vivo POC including tissue selectivity, i.e the PROTAB strategy described by Marei et al. with tumor specific degradation in vivo.
Hence, one would want to see POC EndoTag that this is amenable in vivo and has some tissue selectivity. It is not clear to me whether it is attainable for targets where (e.g.) TfR vastly outnumbers the target.
3) Scope: Several targets are investigated using Endotag to claim a broad scope of this platform but the generalizability isn't really addressed. To me, the main limitation of all of the studies so far presented is that they have failed to enable a target that would not be addressable with conventional therapeutics and could only be enabled with the novel proposed framework. All of the targets presented here are nice POC but they are not novel. For publication in nature, one would either see Endotag on a new target or demonstration of superiority of EndoTag over standard of care in an in vivo setting, including improved depth of degradation, pathway inhibition and efficacy. The degradation levels are overall on par with what everyone else sees.
4) Mechanism of degradation: Surprisingly, little is shown to detail the MOA of EndoTag. The proposed mechanism seems to be inferred by the design, ie oligomerization or conformational change related to the EndoTag design, but how do they authors know this is the case with high certainty? For instance, for oligomerization, size exclusion chromatography could be performed. Further mechanistic studies, for instance: MOA of membrane clearance, trafficking and route of degradation or additional characterization of how relative expression levels of the receptor impact activity could be reported.
5) The binding selectivity of the various EndoTags is unclear.

Author Rebuttals to Initial Comments:

Rebuttal letter

Nature 2023-06-10015A

All original feedback and comments from the reviewers are colored in **grey*
All responses from the authors are colored in **blue*

Summary

We appreciate all the input, feedback, and suggestions from the reviewers and the editors. In this revised version of the manuscript, we have conducted new experiments to address the comments and concerns about the *in vivo* efficacy, orthogonality, and superiority of our EndoTags over other platforms. Here are the highlights of the new results:

1. ***In vivo* efficacy**
   a. We conducted an *in vivo* anti-tumor study in a syngenic mouse model comparing the efficacy among FDA-approved anti-PD-L1 antibody Atezolizumab (ATZ) treatment alone or ATZ genetic fusion with IGF_EndoTag (ATZ-IGF_EndoTag4). The ATZ-EndoTag fusions showed significantly better tumor size, volume reduction ability, and survival profile than ATZ treatment alone (**Fig. 2i-l**). This study provides remarkable proof-of-concept evidence that degrading PD-L1 is more beneficial than simply inhibiting PD-L1.
   b. There was no significant body weight loss in the ATZ-EndoTag treated group, indicating a good safety profile for the ATZ-EndoTag fusion (**Fig. 2m**).

2. **Orthogonality**
   a. We showed that TfR_EndoTag did not interfere with normal Transferrin (Tf) on-cell binding or cellular uptake (**Extended Data Fig. 9d-e**)
   b. A recent study by the Bertozzi lab [Ahn, Green, et al. Science (2023)] suggested that the mannose-6-phosphate (M6P)-based LYTAC system was endogenously competed with M6P-tagged lysosomal hydrolases, limiting the maximal degradation capacity through this receptor. We conducted a new cell assay showing that the IGF_EndoTag was orthogonal to M6P-based ligands or M6P-tagged hydrolases. (**Extended Data Fig. 9c**).
   c. In a confocal microscopy assay, we showed that IGF-2 can still internalize after IGF_EndoTag pretreatment, and the low-affinity IGF_EndoTag4 has no interference with IGF-2 internalization, suggesting the tunability of the EndoTag (**Extended Data Fig. 9a-b**).

3. **Superiority over other platforms**
    a. M6P-tagged hydrolases limit the degradation capacity of M6P-based LYTAC. As mentioned in **2b** above, we showed that the IGF_EndoTag has no competition issue with such hydrolases, as it is orthogonal to M6P binding.
    b. We conducted a head-to-head comparison of EGFR degradation ability between Cetuximab-M6P versus Cetuximab-IGF_EndoTag. Results indicated that Cetuximab-IGF_EndoTag achieved much better EGFR degradation performance than M6P-based LYTAC (**Fig. 2e**).
    c. As mentioned in **1a** above, we showed that PD-L1 degrader (ATZ-EndoTag fusion) achieved significantly better anti-tumor performance than PD-L1 inhibitor (ATZ alone) *in vivo* (**Fig. 2i-l**). To our knowledge, no published results show better in vivo degrader performance than standard therapy for known extracellular degrader systems such as LYTAC or KineTAC.

We have conducted a variety of additional experiments to address the specific questions or concerns of the reviewers; please find our response for each reviewer below:

For Reviewer #1

This work reports an extracellular degrader technology based on de novo designed binders that attach and degrade soluble or membrane targets of interest, EndoTags. The authors also report a logic gated strategy that further increases specificity of the degrader and cell signaling activated by the tag in a synthetic notch system. The work utilizes an impressive suite of de novo design approaches previously pioneered by the Baker lab to find de novo helical bundles that bind in both ligand and non-ligand sites. It directs binders to two well-established recycling receptors, ASGPR and IL-2R/CI-M6PR and two others, the transferrin receptor and sortillin. They show similar levels of degradation as seen by others using similar targets (PD-L1, EGFR) as well as some new ones. The work is very well done, and they show an impressive number of designs and applications.

There are, however, several aspects that reduce the impact of the work especially for a journal like Nature that focuses on paradigm shifting research. There have been over the past few years a number of extracellular degrader platforms that have been described, including the two acknowledged such as LYTACs, and KineTACs. It is not clear what significantly distinguishes the EndoTags that would constitute a breakthough. The authors make the theoretical argument that other platforms like LYTACs and KineTACs, have depended on endogenous ligand-receptor interactions which could have several drawbacks in that they could be affected by competition with endogenous ligand and triggering of unwanted biology. However, the authors do not show this is a convincing advantage for EndoTags. They argue that EndoTags are genetically encoded which has advantages to the LYTAC platform which uses glycan conjugation. But other platforms such as KineTACs are also fully genetically encoded. EndoTags are fully non-natural which could trigger immunogenicity issues if used therapeutically as they suggest. Moreover the design platform is very unique to Baker group and is non-trivial to apply. Thus, it is not clear why other investigators would adopt EndoTags over traditional antibodies or single domain binders, scaffolds which are validated clinically, and use technologies for finding binders that is far simpler and more accessible. It also depends on having a structure of the protein target but this is less of a barrier these days with good predictive modeling methods. While the work is impressive in scope, in our view it does not really cut a new path that many investigators will follow either from a technical or biological perspective. Thus, we would recommend it be published in a high quality but more specialized journal such as Nature Biotechnology or Nature Methods. Below are additional comments that could improve the paper.

We sincerely appreciate the reviewer's feedback and the reviewer's concern about the paradigm-shifting impact of the EndoTag platform. We believe EndoTags can be

significantly distinguished from other platforms to constitute a breakthrough for the following reasons:

1. The development of protein agonists or synthetic protein ligands for endocytosis is a grand challenge. Engineering native ligands is possible when ligands are known, but for receptors without a known ligand, the generation of the corresponding agonists to trigger endocytosis is hard. Antibodies/nanobodies can be raised to bind a specific epitope, but it is extremely challenging for antibodies/nanobodies to become agonists due to their relatively large size and poor control of distances and orientations. To bridge this gap, we created this general computational design method that can rationally generate non-existing ligands/agonists to trigger endocytosis specifically with different mechanisms, including controlling conformational change and multivalency. In the IGF_EndoTag example, we showed that we can rationally design two small binding proteins (65aa) at two different sites and then elegantly tune the relative orientations and angles between the two sites within proximity to achieve different orientations, and thus achieve optimal endocytosis efficiency. This rational control of orientations within a small space cannot be done by antibody/nanobody screening.
2. The current knowledge of endocytosis receptors available for extracellular protein degradation is limited. We further expanded the scope of potential LYTAC receptors to include Sortilin and TfR, showing that both receptors can be leveraged for extracellular targeted protein degradation, which has not been reported before. This breakthrough could guide and enable the generation of novel therapeutics using the degradation pathway of these two receptors with distinct tissue distribution.
3. Standard inhibitor-based anti-PD-L1 therapy is known to trigger PD-L1 compensation pathway to recycle PD-L1, thus limiting the overall therapeutic efficacy and causing relapses [Wu, Yilun, et al. Frontiers in immunology (2019)]. Our anti-tumor experiments in a syngeneic mouse model provided evidence that degradation of PD-L1 is more beneficial than inhibiting PD-L1 *in vivo* context (**Fig. 2i-m**). To our knowledge, no published results show better in vivo degrader performance than standard therapy for known extracellular degrader systems such as LYTAC or KineTAC. We provide very early proof-of-concept results showing that degraders could be more effective than standard inhibitors in vivo.
4. We provide the first proof-of-concept of a signaling enhancer system (**Fig.5**) by combining EndoTag with a signaling ligand bound to a signaling receptor, which could provide significant application for improving cell therapy like CAR-T or enhancing the agonism for native ligands that rely on endocytosis to trigger downstream signaling.
5. We provide proof-of-concept of a modular degrader system (**Fig.4**) that can conditionally degrade receptors with the presentation of another receptor,

which could provide high specificity and selectivity for dual-targeting against multiple cancer markers (e.g., EGFR and HER2).

Immunogenicity:
We agree with the reviewer's points about the immunogenicity of the de novo protein since they are novel proteins whose sequences have not been seen by the immune system before. However, novel sequences may not necessarily lead to high immunogenicity. For example, we did not observe significant weight loss in a mouse study for ATZ-EndoTag, indicating an acceptable safety profile of the EndoTag (**Fig. 2m**). Previously, the Baker lab has successfully translated several de novo proteins to clinical stage drug candidates without significant problems from immunogenicity (e.g., NL-201 from Neuleukin, TAK-062 from PvP Biologics, IVX-A12 from Icosavax). We hypothesize that because designed minibinders are small, stable, and soluble, they may not be taken up and presented on dendritic cells. On the other hand, even fully humanized antibodies can sometimes have immunogenicity issues [FA Harding, et al. MAbs. 2010.]. Therefore, we believe each protein therapeutic requires individual pharmacological assessment and optimization of immunogenicity for therapeutic usage, which has been relatively mature in the current pharma industry.
From our perspective, there are also several strategies to reduce immunogenicity:

1. With the ProteinMPNN [Dauparas, Justas, et al. Science (2022)] tool for protein sequence optimization, we could generate protein sequences with a bias on human-like sequences to potentially reduce the risk of immunogenicity.
2. With the peptide-MHC recognition tool developed by our lab [Motmaen, Amir, et al. PNAS (2022)], we are able to avoid protein sequences that can be recognized by the MHC system.
3. By fusing the EndoTag with established antibodies known to have low therapeutic immunogenicity, we can take advantage of the pharmacological properties of the antibody to shield the EndoTag, given the EndoTag sequence is much shorter relative to the antibody sequence.

Design platform hard to follow:
We thank the reviewer for bringing up the platform accessibility issue, and we acknowledge the reviewer’s suggestion on improving the impact of science for this manuscript. We agree that many of the tools and software might have a learning barrier for new users. Nonetheless, our lab continues to work hard to develop open-source and easy-to-use protein design methods for everyone, with the Rosetta software suites and newest RFdiffusion, ProteinMPNN, and

RosettaFold2 open-sourced with detailed protocols and documentation. People without much knowledge about protein design can use these tools to design proteins with a single command using a PC/Laptop with a GPU. We believe with our continuous endeavor, more and more users will be able to join our community and expand our open design platform with ease of usage.

We have also included a statement in the “induced conformational change” section of the main text to highlight the unique tunability of EndoTag over conventional antibody/nanobody scaffolds.

Superiority over other genetically encodable platforms (e.g., KineTAC): KineTAC is an excellent genetically encodable platform leveraging the native chemokine CXCR12. It has achieved remarkable degradation capacity with reduced downstream signaling activation through its native signaling receptors, while we focus more on a general protein design method that can be adapted to newly discovered endocytosis receptors without a known ligand. While we firmly believe each platform has its optimal application context and showing superiority is not necessary, we have collected data suggesting that the EndoTag platform has considerable anti-tumor efficacy compared to standard anti-PD-L1 therapy under *in vivo* context (**Fig. 2i-m**), which other major extracellular degrader platforms like KineTAC and LYTAC have not reported.

Additional Comments:
1. The authors argue that the EndoTag system is bio-orthogonal by desinging binders do not bind where the natural ligands bind. In some cases, biorthogonality is obviously not achieved – the IGF-2R EndoTag would surely compete with natural ligand binding based on the structures in Fig 1. Even if the sites do not overlap, it is still possible the EndoTags affect the dynamics or have allosteric effects on the degrader that does impact its natural function. The authors have not tested the natural ligand functional effects from what I see here. For example, does the TfR EndoTag impact transferrin uptake. These are relatively simple things to test and important to add if they claim these will not impact native function. Moreover, even if shown to be benign, that isn’t enough to say EndoTags have advantages to other systems that bind the natural ligand site unless they compare them head-to-head. Abundant recycling systems like the TfR can be far more abundant than that needed to degrade the protein of interest, so whether one binds at the ligand binding site or not it doesn’t matter.

We sincerely appreciate the reviewer's comments about the bio-orthogonality of our EndoTag system. Here are our responses regarding the orthogonality of our individual EndoTag the reviewer has raised concerns about:

Orthogonality of IGF-2R based EndoTag to M6P:
The Bertozzi lab [Ahn, Green, et al. Science (2023)] showed that a M6P-based LYTAC system against M6P-tagged lysosomal hydrolases limits the maximal degradation capacity through this receptor. Therefore, an orthogonal system avoiding competition with M6P-tagged hydrolases should achieve better degradation capacity. So, our major focus was to develop an EndoTag system that can bind to a binding site in IGF-2R that is orthogonal to M6P. To show the orthogonality of our IGF_EndoTag to M6P-tagged hydrolases, we conducted new cell assays comparing the IGF_EndoTag on-cell binding between UMRC2 wild-type (WT) cells and UMRC2 M6P-biosynthesis knockout cells (GNPTAB KO). The cell surface binding of IGF_EndoTag in UMRC2 GNPTAB KO cells has no significant increase compared to UMRC2 WT cells (**Extended Data Fig. 9c**), indicating the IGF_EndoTag is orthogonal to M6P-based ligands or M6P-tagged hydrolases. In contrast, the M6P-based LYTAC had significantly increased cell binding in UMRC2 M6P-hydrolase knockout (KO) cells compared to WT cells [Fig 6d from Ahn, Green, et al. Science (2023)].

With this advantage in orthogonality to M6P-tagged hydrolases, we ran the head-to-head comparison of our EndoTag system versus the M6P-based LYTAC. A western blot on EGFR levels suggested that Cetuximab-IGF_EndoTag achieved better EGFR degradation compared to M6P-based LYTAC (**Fig. 2e**).

Orthogonality of IGF-2R based EndoTag to IGF-2:
We appreciate the reviewer's concern about the potential competition of our IGF_EndoTags to IGF-2R since they are designed to share similar binding sites at IGF-2R domain11, even though the IGF_EndoTags were originally designed to be orthogonal to M6P binding sites. We carried out a confocal microscopy assay where we pre-treated the Hela cells with non-labeled EndoTags, then washed and added fluorescence-labeled IGF-2 and lysotracker for co-localization. We showed that the IGF-2 can still internalize and localize with lysosome after IGF_EndoTag pretreatment, and the low affinity IGF_EndoTag4 has no interference on IGF-2 internalization at all, while the high affinity IGF_EndoTag2 has slight interference on IGF-2 lysosome co-localization (**Extended Data Fig. 9a-b**). This finding suggests that by fine-tuning the binding affinity of EndoTag to the IGF-2R domain11, we can avoid the interference of the native IGF-2 trafficking pathway to the lysosome for its native degradation pathway through IGF-2R. Our Mass Spectrometry data (**Fig. 2g**) also showed that the IGF-2R level maintained the same after EGFRn-IGF_EndoTag treatment, suggesting that the degradation of EGFR will

not sacrifice the IGF-2R or affect IGF-2R recycling pathway, providing another evidence that the EndoTag will not interfere with the native IGF-2R pathway.

Furthermore, it is noteworthy that the primary role of IGF-2 is activating the IGF-1R-based signaling pathway for tissue growth and proliferation, while its binding to IGF-2R is a regulatory pathway to control its level in the blood [Blyth, Andrew J., et al. Cells (2020)]. In a biolayer interferometry (BLI) assay, the IGF_EndoTag didn't show binding signal to IGF-1R at 1 uM concentration, providing evidence that our system will not interfere with its main function in signaling through IGF-1R.

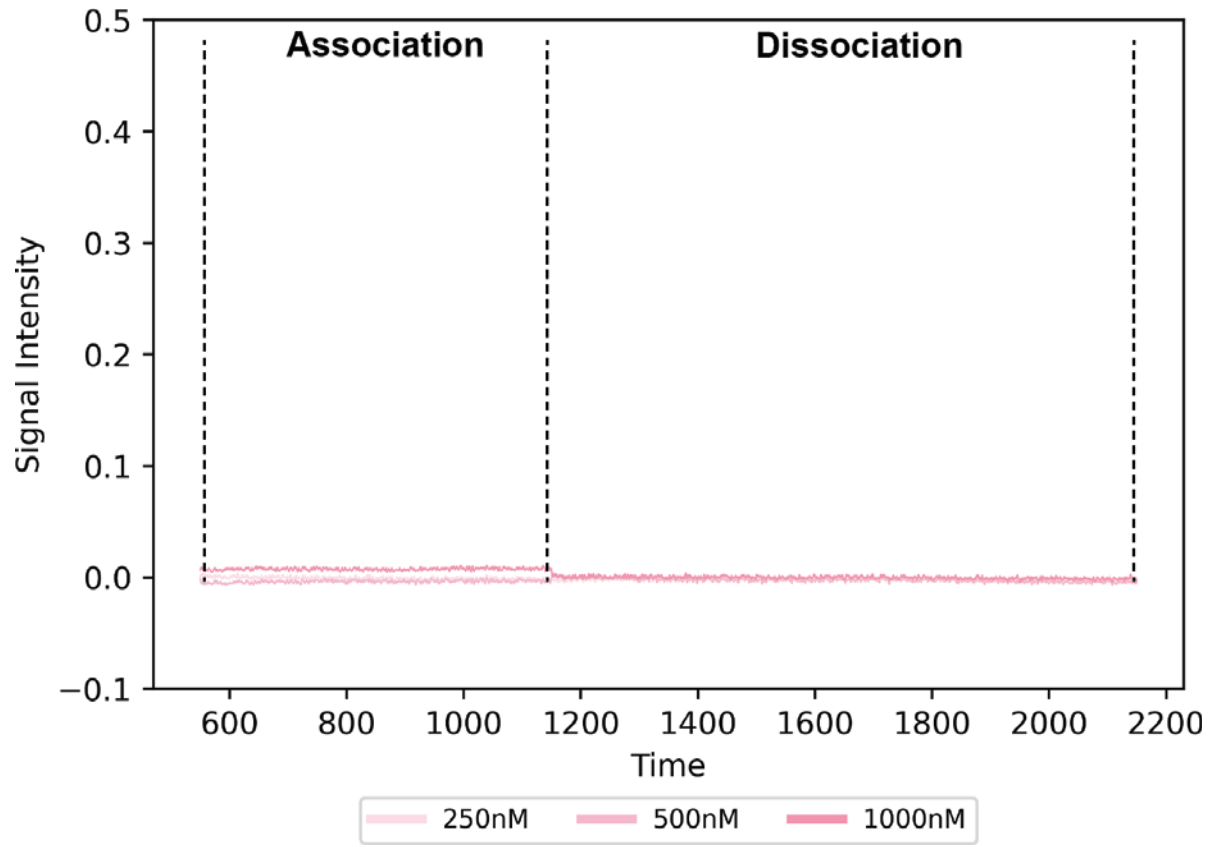

Orthogonality of TfR-based EndoTag:

We conducted the Tf cell binding and cellular uptake assay as the reviewer suggested, and found that our designed TfR_EndoTag didn't interfere with normal Tf on-cell binding (**Extended Data Fig. 9d**) or cellular uptake (**Extended Data Fig. 9e**), indicating its orthogonality to native ligands.

[Text Redacted]

[Figure Redacted]

Minor comments/questions:

1. Sometimes the exact lysosomal marker is not defined for confocal images. Please specify.
We thank the reviewer for pointing out the lysosomal marker issue for confocal images; we have updated the figure legend of Fig.3f, Fig.3g, and Fig.5e to specify the exact lysosomal marker.

2. Line 90 – what are “all 4 receptor systems”?
We apologize for the confusion of this description. We have modified the main text to resolve ambiguity.

3. Line 110 – Neurotrophins are a broad class. Do they all bind to the same site?
We thank the reviewer for catching this mistake. We should have used the word “Neurotensin,” but we made a typo and used the word “Neurotrophin.” As pointed out by the reviewer, Neurotensin represents a broad class of molecules, including NGFs and NT3-5. We modified the word to be Neurotensin in the main text, as we have used the solved crystal structure of Neurotensin in complex with Sortilin (PDB: 4PO7) to generate Fig.1b superimposition schema.

4. Line 122 - Naming conventions – What is the difference between sortmb and Sort_EndoTag?
We thank the reviewer for raising this naming convention issue. Sortmb and Sort_EndoTag are essentially the same thing. Originally, we referred to sortmb as the binder we screened that had good binding affinity to Sortilin, while we converted its name to Sort_EndoTag during validation of its lysosomal targeting ability. We have added more descriptions highlighting the renaming from sortmb to Sort_EndoTag in the main text to avoid ambiguity.

5. Line 125 – Extensive washing is great but doesn’t completely rule out surface binding, are there any controls to negate this (e.g. internalization at 4C with washing step)?
We acknowledge the reviewer’s concern about the washing issue for the internalization experiment. First, we have measured the binding affinity of Sort_EndoTag (Extended Data Fig. 2g) using BLI assay, and its off-rate is quite

fast. Therefore, we believe the extensive wash is adequate to completely wash out the surface binding. Second, we fully agree with the reviewer that the flow cytometry-based internalization assay is not perfect, since we cannot differentiate between surface binding versus internalization, even with extensive washing. Therefore, we further conducted confocal imaging to evaluate the lysosome co-localization. By combing the flow data and imaging data, we can safely conclude that the Sort_EndoTag internalizes inside the cells.

6. Line 174 – We would have liked to see monomer versions of D6 and D11 binders to compare. It's possible that making dimers out of single domain binders simply ablates all binding to the receptor.
We thank the reviewer's comments. We included the monomer version of D6 and D11 binders in the cellular uptake comparison shown in **Extended Data Fig. 4c**.

7. Line 259 – There is no figure 1k
We thank the reviewer for carefully catching this typo; we have modified the figure label in line 259 to be Fig. 1i.

8. Line 269 – Ideally more brain relevant cell lines would be used to show that TfR and Sort tags could work in the brain.
We appreciate the reviewer's suggestion about the brain-relevant cell lines. We did include the cellular uptake data of Sort_EndoTag and TfR_EndoTag using U251 glioblastoma cell lines in Fig. 1i. Since the manuscript focuses primarily on the protein design approach to develop the EndoTag, we think this proof-of-concept cellular uptake assay using U251 glioblastoma is adequate to support our claim.

9. Figure 2 – an EndoTag alone control would be good to see to ensure the tag isn't affecting anything.
We thank the reviewer's suggestion for adding the EndoTag alone control. We conducted a flow cytometry assay to evaluate surface EGFR degradation. For the treatment groups, the control is the untreated group, pLYTAC20 refers to IGF_EndoTag1, and Ctx refers to Cetuximab. As shown in the figure below, the pLYTAC20 (IGF_EndoTag1) group didn't affect the EGFR level at all, which supports the claim that the EndoTag alone isn't affecting anything.

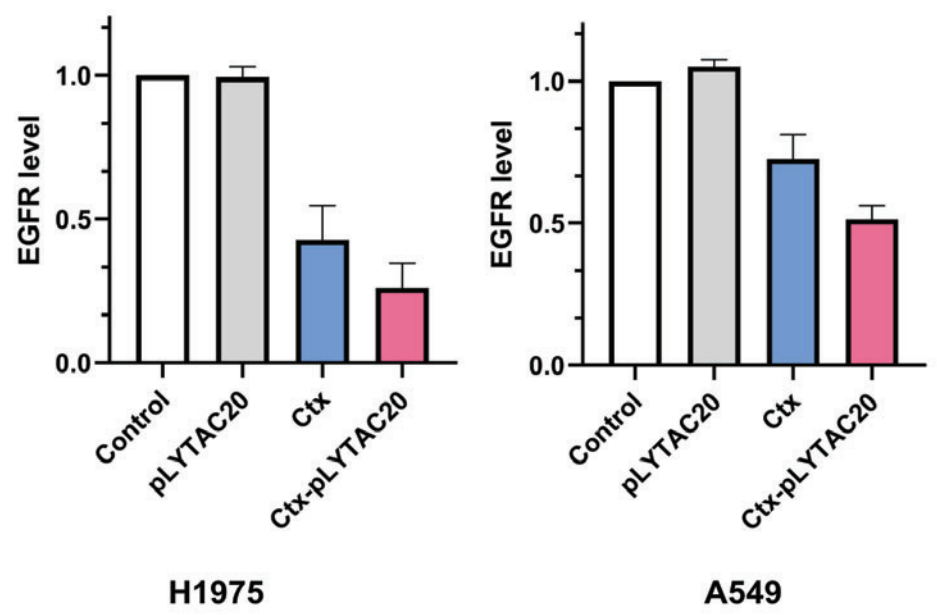

10. The MS data is a bit underexplained in my view. What is the control? What are other downregulated hits? Do these support the position that the tags are biorthogonal?

We appreciate the reviewer's suggestion on MS data to improve the manuscript. The control is the untreated cells. We have updated the figure legend for Fig. 2g to reduce ambiguity.

We have added a new table in the manuscript to highlight the quantified level of certain proteins with a downregulated level larger than 40% in the EGFRn-IGF_EndoTag2 treated group versus the untreated control (**Extended Data Table. 2**). The EGFR degradation is with high statistical significance (p=0.0008) in the EGFRn-IGF_EndoTag2 treated group compared to the untreated group (control), while there is no significant EGFR level reduction for EGFRn group. For all other proteins in the list with more than 40% reduction, none of their abundance reduction levels are statistically significant. For most of these proteins, we observed a reduction trend in the EGFRn-treated group compared to the EGFRn-IGF_EndoTag2-treated group. These results suggest that EGFRn-IGF_EndoTag2 didn't induce significant non-specific downregulation on the proteomics.

11. Overall, many figures have "control" listed as one of the conditions. It is not always clear what this control is. Is this an isotype control or simply vehicle treated, etc?
We thank the reviewer for the comment about the clarification of the control group. The "control" either refers to the vehicle-treated or untreated group. To avoid ambiguity, we have specified all the "control" groups used for the corresponding experiments in the main text and figure legend (mainly cover Fig. 1c, Fig. 3e, Extended Data Fig. 4, Extended Data Fig. 6).

12. Line 327 – Fold change over what?
We thank the reviewer for the question. It is the fold change over the proteinG-alone treated group. We have modified the main text correspondingly.

13. Figures 3f&g would be nice to see colocalization with a merge
We acknowledge the reviewer's suggestion to show colocalization with a merge figure. We have included a new figure in the supplement with the merge across all 3 channels (488 Lysosome (green), 588 IGF2R (blue), and 647 IgG Ligand (red)) with 3 biological replicates in Hela WT cells (**Extended Data Fig. 16a**) and Hela IGF-2R KO cells (**Extended Data Fig. 16b**).

14. In the case of AND gated degraders, it seems from the diagram alone that the gating receptor would also get degraded. Is this happening? This could be a problem if undetected.
We thank the reviewer's comment about the degradation of the gating receptor. We originally designed the system to be a dual-receptor degrading system, so both the gating receptor and the target receptor will be degraded. Such a system is most relevant in cancers with two overexpressed cancer markers (e.g., EGFR+/HER2+ breast cancer, where both markers are detrimental) in order to achieve better specificity.

15. For cell signaling activation of the Ortho-SNIPR, why was LHDA dimer used as the comparison? How was the linker chosen for this dimer?
We thank the reviewer for bringing up the concern about the LHDA dimer control. The LHD-A dimer was used to show that dimerization of the receptor alone was insufficient to induce signaling and support the hypothesis that endocytosis, as opposed to receptor dimerization, is the mechanism of action. We chose a short GGS linker to guarantee the receptors can interact. In addition to the LHD-A dimer, in the accompanying manuscript (Ortho-SNIPR), we have compared the signaling activation of LHD-A monomer and LHD-A dimer; neither induces a significant increase in signaling activation than the untreated control.

16. Line 418 – not sure if the data entirely supports this without a competition experiment. It is true they were designed this way and match the epitope by N-glycan attachment.
We appreciate the comment from the reviewer about the competition to determine epitope specificity. For Sortilin, as highlighted by the reviewer, our N-linked glycan competition data suggests that the Sort_EndoTag is epitope-specific. For TfR_EndoTag, a previous paper [Sahtoe, Danny et al. PNAS. (2021)] from our lab has included the crystal structure of the Tf binder in complex with Tf. The TfR_EndoTag used in the current manuscript is a slightly mutated version of the Tf binder with the solved structure for better solubility with all interface residues fixed, so we can assert the Tf_EndoTag possesses the epitope specificity. For AS_EndoTag, there are ongoing projects leveraging

AS_EndoTag as a liver-specific delivery reagent, and we will conduct structure determination on AS_EndoTag in complex with ASGPR, which will be a follow-up paper.

17. Line 431 – One limitation is that a structure or structural model of the receptor- native ligand complex is required to design an EndoTag that does not sterically occlude ligand binding. This is a higher bar than having a good model of the receptor alone. The authors should discuss this

We thank the reviewer for the comments about the requirement for a ligand-bound model to initialize the design. We agree with the reviewer that a ligand-bound receptor structure could provide important insight to avoid sterical competition with the native ligand, and it indeed leads to a higher bar than having a good receptor structure alone. Nonetheless, a solved complex structure is not always necessary, especially when paired with relevant experiments. On the one hand, the latest docking/structure prediction tools such as RosettaFold2-All-atom [Krishna, et al. biorxiv. (2023)] and Alphafold2-latest are reported to achieve accurate docking predictions of protein or chemical ligands in complex with a protein receptor. Therefore, for ligands with known monomer structures, we could employ such tools to predict potential binding sites and make designs orthogonal to that site. Furthermore, we can design binders at multiple available sites in the given receptor without knowing which sites are orthogonal, then use experimental screening to remove candidates that show competition with the ligand. The binder design protocol described in this manuscript and many other papers from Baker lab (e.g., [Watson, et al. Nature. (2023)], [Cao, et al. Nature. (2021)]) have shown that a de novo binder can be generated to bind at arbitrary binding sites, as long as the site contains a small hydrophobic patch with little backbone flexibility. Therefore, starting with the receptor model alone, it is still possible to generate an orthogonal synthetic binder/ligand.

18. Line 442 – This is a great point, but really makes one ask why a synthetic system was used to show signal activation. Natural receptor activation is much more relevant and impactful.

We thank the reviewer's comment about why a synthetic ligand/receptor pair is more beneficial than a natural system. We agree with the reviewer that most natural ligand/receptor pairs can achieve considerable signaling activation. In this manuscript we provide a proof-of-concept study exploring the possibility of EndoTag to enhance signaling for a synthetic system. We suppose that for many tasks involved with ligands related to tissue regeneration or differentiation, a superagonist consisting of an EndoTag and a ligand with stronger signal activation than the native one could be beneficial.

For Reviewer #2

Prior work published in Nature from the Baker and the Bertozzi labs respectively established de novo protein binders design with Rosetta (Nature 2022) and chimeric antibody-based degraders (LYTACs) for degradation of extracellular and transmembrane proteins via endocytosis-lysosomal pathways (Nature 2020). Here, Bertozzi and Baker have teamed up to develop a chimeric protein based strategy to induce endocytosis of extracellular and transmembrane proteins – an approach they here call EndoTags. The design EndoTag as binding proteins for 5 different receptors triggering endocytosis via various mechanisms, and then go on to conjugate those to transmembrane proteins (EGFR, PD-L1) as well as soluble proteins, showing their endocytosis-driven degradation. They also show utility of their approach for a RNA-based delivered secretory approach, and triggering of transcription factor based signaling upon target degradation.

There are many positives to this article. The work is of extremely high quality and reach, as expected by these two laboratories, offering an enticing proof of concept for bringing Rosetta-based protein design towards applications for targeted protein degradation. This could offer advantages relative to the antibody-based approach currently explore via LYTAC – however a limitation of the study is that in the few experiments where a side-by-side comparison of an EndoTag-based versus conventional LYTAC (antibody) based degraders the improvement in performance appears minimal, so such advantages are not strictly established. Other positives are the wide-range of receptors, proteins and mechanisms targeted in the study which evidence scope and power of the approach. Another negative is the therapeutic potential and appropriateness to drug development are not demonstrated; for example, do these agents work in vivo? Could they exhibit improved PK properties over e.g. conventional LYTACs which suffer from the antibody-based scaffold? Overall, the paper is nonetheless exciting and could eventually be published in a high-impact journal as a significant advance in the field.

Superiority over LYTAC:

We sincerely appreciate the reviewer's feedback on the lack of side-by-side comparison to conventional LYTAC to improve the current manuscript. Our original goal of developing an orthogonal IGF-2R-based EndoTag system was mainly raised from a recently published study by the Bertozzi lab [Ahn, Green, et al. Science (2023)], where we showed that M6Pbased LYTAC system was endogenously competed with M6P-tagged lysosomal hydrolases, limiting the maximal degradation capacity through this receptor. So, our major focus is to develop an EndoTag system that can bind to a binding site in IGF-2R that is

orthogonal to M6P. We conducted a new cell assay comparing the IGF_EndoTag on-cell binding between UMRC2 WT cells and UMRC2 M6P-hydrolase KO cells. The cell surface binding of IGF_EndoTag in UMRC2 M6P-hydrolase KO cells has no significant increase than UMRC2 WT cells (**Extended Data Fig. 9c**), indicating the IGF_EndoTag is orthogonal to M6P-based ligands or M6P-tagged hydrolases. On the contrary, the M6P-based LYTAC had significantly increased cell binding in UMRC2 M6P-hydrolase KO cells compared to WT cells [Fig 6d from Ahn, Green, et al. Science (2023)].

With the advantage of orthogonality to M6P-tagged hydrolases, we ran the head-to-head comparison of our EndoTag system versus the M6P-based LYTAC. A western blot on EGFR level suggested that Cetuximab-IGF_EndoTag achieved better EGFR degradation compared to M6P-based LYTAC (**Fig. 2e**).

Therapeutic potential:
We highly value the reviewer's concern about the therapeutic potential of the EndoTag system for drug development. With the newly included mouse study where we established a syngenic mouse model, we showed that PD-L1 degrader (ATZ-EndoTag fusion) achieved better anti-tumor performance than PD-L1 inhibitor (ATZ alone) *in vivo* with significantly improved survival (**Fig. 2i-l**), which provided a proof-of-concept therapeutic application of the EndoTag approach over the standard immune checkpoint inhibitor therapy.

Regarding the reviewer's question about PK, we don't expect our EndoTag to prolong the half-life or the PK profile compared to the conventional LYTAC or antibody system. We believe the PK of drug candidates needs to be individually optimized by the mature pharma industry for certain diseases. In general, with the small size of the de novo–designed proteins, our EndoTags can have a relatively short half-life due to quick kidney filtration if fused with a small peptide or designed miniproteins. By genetically fusing EndoTags with conventional antibodies that possess a long half-life containing the Fc domain, we could rely on the PK profile of the original antibody. Another factor that might affect the PK is the tissue distribution of the corresponding endocytosis receptors leveraged by the EndoTags. Since our system is designed to rapidly activate endocytosis upon binding to the receptor, it can target to the organs/tissues that have more corresponding receptors and get internalized/removed in the blood rapidly. That explains why generating a large scope of EndoTag targeting a variety of receptors with distinct tissue distribution is useful, allowing EndoTags to be picked and modularly fused with different antibodies to achieve desired therapeutic application in certain tissues.

In addition to the limitations highlighted above, to strengthen and improve the paper, the authors might want to consider including “negative control” non-binding EndoTags, ideally single-point or multiple mutants of otherwise identical fold, to evidence specificity – much in the same way as non-binding ligands for the E3 ligase VHL/CRBN are widely used in the PROTAC field as they lead to non-degrading molecules of otherwise very similar physico-chemical properties. It would also be interesting to evidence the kinetics of protein degradation – e.g. is the target depleted in minutes, hours, days? I notice in many cases the blots are from cells treated for up to 48 h.

Non-binding EndoTag control:
We fully align with the reviewer's feedback regarding the degradation specificity. We followed the reviewer's suggestion and selected IGF_EndoTag and Sort_EndoTag as examples. We introduced 5 interface mutations on IGF_EndoTag2 to diminish its binding affinity to IGF-2R based on the mutation scanning assay we did previously (not disclosed in the manuscript). Similarly, we introduced 2 mutations on the Sortilin binder at the Sortilin binding interface. After fusing the EGFRn binder with the scaffold mutants, we compared their EGFR degradation ability with the WT EGFRn-EndoTag. In Hela cells after 48h treatment, the EGFR degradation ability is fully ablated for both the IGF_EndoTag2KO and Sort_EndoTagKO, indicating that the degradation is specific for both EndoTags (**Extended Data Fig.10**).

Kinetics of protein degradation:
We agree that the kinetics of protein degradation are essential for the therapeutic aspect. Therefore, we evaluated the surface PD-L1 level across multiple time points with the ATZ-IGF_EndoTags. All 3 candidates showed significant PD-L1 degradation after a 4 h treatment, where both the ATZ-IGF_EndoTag2 and ATZ-IGF_EndoTag4 led to 60~70% depletion of PD-L1. The maximum degradation capacity was reached at 24 h and lasted for 48 h (**Extended Data Fig.6h**). This trend was held for all 3 EndoTag candidates tested. Additionally, we tested CTLA4 degradation using our designed CTLA4mb-IGF_EndoTag. Within 3 h, the fusion was able to clear 50% of CTLA4 (**Extended Data Fig.6c**). We believe that the degradation kinetics is target-dependent, factors including target abundance on the cell surface, target re-synthesis rate, and target size might all affect the actual degradation kinetics.

For Reviewer #3

In this manuscript, Huang et al. present a protein design strategy to exploit the endocytic cell machinery to ultimately affect membrane receptor function and cellular signaling.
The authors have to be commended for putting together a manuscript that is well conceived, use state of the art protein design and innovative approaches to drive receptor internalization. This strategy, as the authors claim, was designed with the goal to differentiate from other approaches by being generalizable, not interfering with the native signaling of the internalizing receptors, by allowing tissue specific activity, and by enabling logic-gated activity. It is fair to say, however, that there have been a good handful of papers that have explored bispecific molecules to drive internalization and lysosomal degradation including the LyTACs, the KINETACs, PROTABs but also de Goeij et al Mol Cell Ther 2016, Andreev et al. Mol Cell Ther 2017, and Devay et al. Bioconj Chem 2017 who all did it to improve ADC activity.

Although the framework presented here has the potential to improve on the limitations of other strategies, little data is actually presented to demonstrate in fact that it does. Without further evidence, I do not think this essential claim can be made.
Major comments are listed below:
1) Bypass of native ligand: While the described peptides do bind at sites on the receptors that are orthogonal to ligand binding sites, there is little data validating that the normal signaling pathways are not impacted (either by less receptor on the cell surface, or by directly inducing signaling).

Bypass native ligand:
We acknowledge the reviewer's suggestion for more data validating that the normal pathway is not impacted. In a confocal microscopy assay where we pre-treated the Hela cells with non-labeled EndoTags, then washed and added fluorescence-labeled IGF-2 and lysotracker for co-localization, we showed that the IGF-2 can still internalize and localize with lysosome after IGF_EndoTag pretreatment. Notably, the low-affinity IGF_EndoTag4 has no interference on IGF-2 internalization at all, while the high-affinity IGF_EndoTag2 has slight interference on IGF-2 lysosome co-localization (**Extended Data Fig. 9a-b**). This finding suggests that by fine-tuning the binding affinity of EndoTag to the IGF-2R domain11, we can avoid the interference of the native IGF-2 trafficking pathway to the lysosome for its native degradation pathway through IGF-2R. Our mass spectrum data (**Fig. 2g**) also showed that the IGF-2R didn’t significantly reduce after EGFRn-IGF_EndoTag treatment, suggesting that the degradation of EGFR will not sacrifice the IGF-2R or affect the IGF-2R recycling pathway, providing

further evidence that the EndoTag will not interfere with the native IGF-2R pathway.

Additionally, it is worth mentioning that the main function of IGF-2 is activating the IGF-1R-based signaling for tissue growth and proliferation, while its binding to IGF-2R is a compensation pathway to control its level in the blood [Blyth, Andrew J., et al. Cells (2020)]. Our binding data below with a BLI assay suggested that our IGF_EndoTag didn't bind to IGF-1R at 1uM concentration, providing evidence that our system will not interfere with its main function in signaling through IGF-1R.

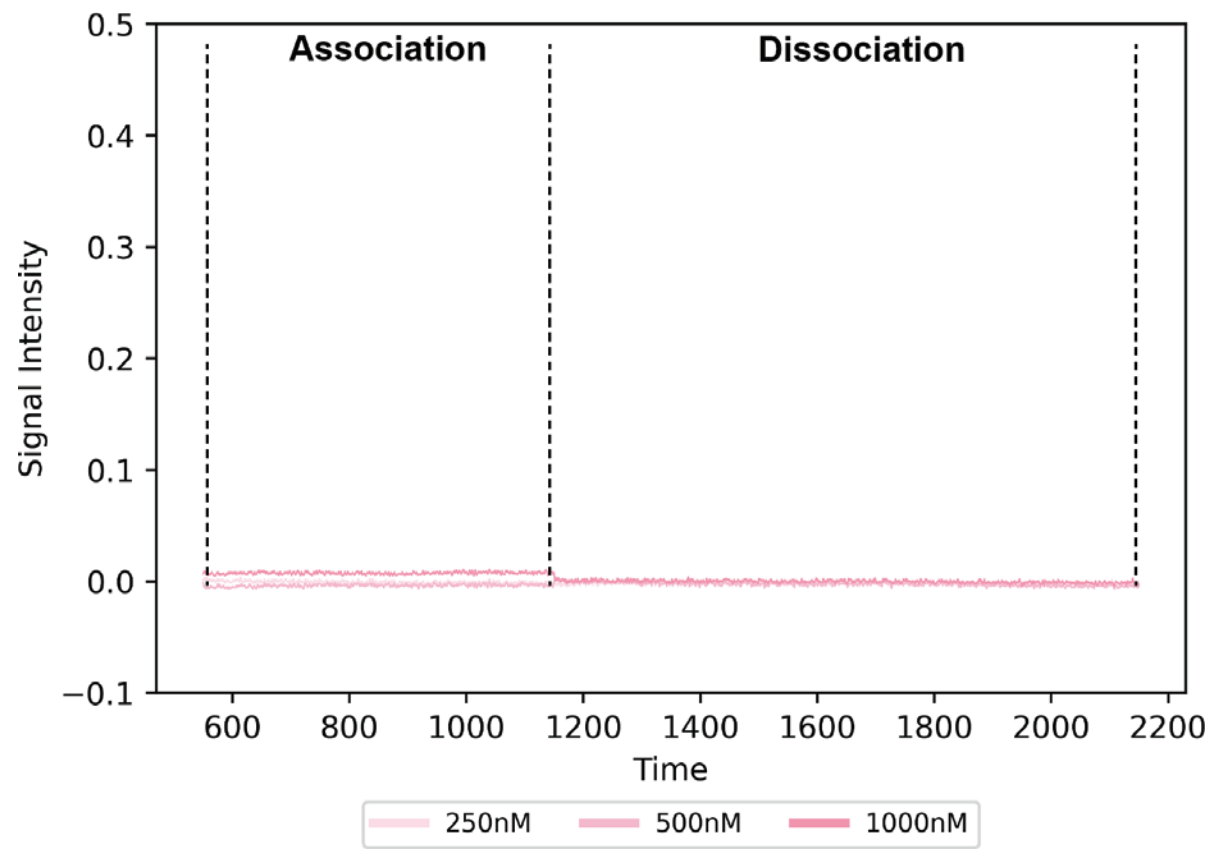

For TfR_EndoTag, we conducted the Tf cell binding and cellular uptake assay as the reviewer suggested. As shown in **Extended Data Fig. 9d-e,** our designed TfR_EndoTag didn't interfere with normal Tf on-cell binding or cellular uptake (**Extended Data Fig. 9d-e**), indicating its orthogonality to native Tf ligands.

2) Tissue selectivity: Many other cell surface degradation studies have demonstrated in vivo POC including tissue selectivity, i.e the PROTAB strategy described by Marei et al. with tumor specific degradation in vivo.
Hence, one would want to see POC EndoTag that this is amenable in vivo and has some tissue selectivity. It is not clear to me whether it is attainable for targets where (e.g.) TfR vastly outnumbers the target.

We thank the reviewer for bringing up the potential tissue selectivity of the EndoTag to improve the manuscript. We conducted *in vivo* POC experiments with the IGF-2R-based EndoTag, and the PD-L1 degrader (ATZ-EndoTag fusion) achieved better anti-tumor performance than PD-L1 inhibitor (ATZ alone) (**Fig. 2i-m**). Due to the broad expression of IGF-2R across all tissues, we are not able to show the tissue specificity *in vivo*. Similarly, as the reviewer pointed out, though TfR is expressed largely in the brain, it is also broadly expressed in

most tissues, so tissue specificity will be a concern in an in vivo context. We have adjusted our statement in the main text about tissue selectivity of TfR.

The other two receptors (Sortilin and ASGPR) could provide better examples of tissue-specific degraders, and we have ongoing work to characterize their tissue specificity. Sortilin has been shown to express dominantly in the brain with slight expression in male tissues and muscle tissues (https://www.proteinatlas.org/ENSG00000134243-SORT1/tissue). We are conducting *in vivo* POC experiments leveraging Sort_EndoTag for brain targeting and delivering of reagents, but it will end up in a separate paper we are preparing. ASGPR has been demonstrated to be highly liver-specific [Ahn, et al. Nature Chem Bio. (2021)]. Since there is a slight structure difference between human and mouse ASGPR, we are still working on affinity optimization of AS_EndoTag to achieve binding to mouse ASGPR, followed by *in vivo* POC for liver targeting; this work will be the follow-up paper with a focus on more in-depth application.

3) Scope: Several targets are investigated using Endotag to claim a broad scope of this platform but the generalizability isn't really addressed. To me, the main limitation of all of the studies so far presented is that they have failed to enable a target that would not be addressable with conventional therapeutics and could only be enabled with the novel proposed framework. All of the targets presented here are nice POC but they are not novel. For publication in nature, one would either see Endotag on a new target or demonstration of superiority of EndoTag over standard of care in an in vivo setting, including improved depth of degradation, pathway inhibition and efficacy. The degradation levels are overall on par with what everyone else sees.

We highly value the reviewer's feedback about the therapeutic potential of the EndoTag system. With the newly included mouse study where we established a syngeneic mouse model (**Fig. 2i**), we showed that PD-L1 degrader (ATZ-EndoTag fusion) achieved better anti-tumor performance than PD-L1 inhibitor (ATZ alone) *in vivo* with significantly reduced tumor volume and weight (**Fig. 2j-k**), and improved treatment efficacy with better overall survival (**Fig. 2l**), which provided a proof-of-concept therapeutic application of the EndoTag approach over the standard-of-care immune checkpoint inhibitor therapy. We further collected the tumor tissues from the mice with different treatments. Western blot quantification of PD-L1 levels showed that ATZ-IGF_EndoTag3/4 induced improved depth of PD-L1 degradation than ATZ treatment alone or the untreated group under *in vivo* context.

4) Mechanism of degradation: Surprisingly, little is shown to detail the MOA of EndoTag. The proposed mechanism seems to be inferred by the design, ie oligomerization or conformational change related to the EndoTag design, but how do they authors know this is the case with high certainty? For instance, for oligomerization, size exclusion chromatography could be performed. Further mechanistic studies, for instance: MOA of membrane clearance, trafficking and route of degradation or additional characterization of how relative expression levels of the receptor impact activity could be reported.

We thank the reviewer for constructive suggestions regarding the MOA of the EndoTag platform. We highly valued the reviewer's comments and included multiple new experiments to answer the reviewer's questions about MOA from various aspects:

MOA for AS_EndoTag oligomerization:
We performed the size exclusion chromatography as the reviewer suggested on the AS_EndoTag in the complex with ASGPR. We mixed the AS_EndoTag-3C with the ASGPR at a 3:1 molar ratio in PBS and injected the mixture into an Akta SEC using an S200 column. SEC traces indicated that compared to the ASGPR alone or AS_EndoTag-3C alone, a mixture of AS_EndoTag with ASGPR induces the formation of two obvious higher-order complex peaks (peak1 at retention V=13ml, peak2 at elution V=13.6ml) and a small monomer peak (peak3 at retention V=15ml) (**Extended Data Fig. 14**). The observed elution volume of peak1 is equal to the estimated elution volume of trimer complex containing 3 ASGPR molecules and 1 AS_EndoTag-3C molecule (estimated MW=111kDa, estimated retention V=13.00kDa). The observed elution volume of peak2 is equal to the estimated elution volume of a dimer complex containing 2 ASGPR molecules and 1 AS_EndoTag-3C molecule (estimated MW=82.5kDa, estimated retention V=13.56kDa). The peak3 is likely because the extra ASGPR molecules didn’t form the complex with AS_EndoTag. In general, these SEC traces indicated that the AS_EndoTag-3C could induce the trimer or dimer complex formation with ASGPR.

MOA for IGF_EndoTag:
For the MOA of IGF_EndoTag-based conformational change, we have tried very hard to determine the IGF-2R/IGF_EndoTag4 complex structure using negative-stain CyroEM. Due to high flexibility, we have limited particles for analysis and are unable to increase the resolution further. From the current low-resolution data, we did observe a conformation similar, but slightly different from, the IGF-2-based structure (PDB:6UM2) after superimposition with IGF-2R/IGF-2 complex, which could partially suggest that our designed IGF_EndoTag can trigger a conformational change upon binding.

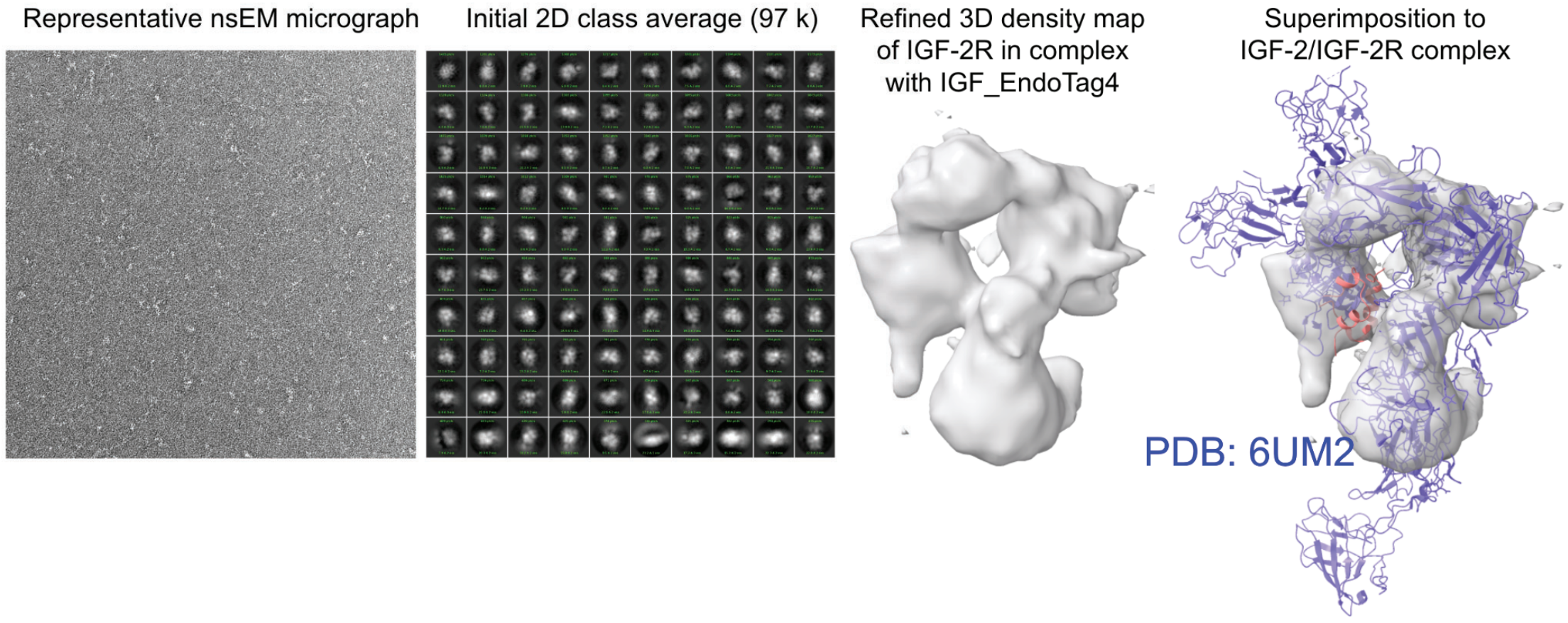

Relative expression level of receptors affects degradation activity
We used IGF-2R-based EndoTag to evaluate the effect of receptor expression on degradation activity. We first ran a western blot screening on IGF-2R across multiple human cancer cell lines (**Extended Data Fig. 12a**). Notably, A431 cells have very low expression of IGF-2R, A549 cells have medium level expression, and Hela cells express relatively higher levels of IGF-2R. We then compared the EGFR degradation capacity of EGFRn-IGF_EndoTag1 across A431, A549, and Hela cells using western blots. Quantified results indicated that the relative expression level of IGF-2R is strongly correlated with EGFR degradation efficiency, as EGFRn-IGF_EndoTag1 degrades 20% of EGFR in low expression A431 cells (**Extended Data Fig. 12b**), 40% of EGFR in medium level A549 cells (**Extended Data Fig. 12c**), and 70% in high IGF-2R Hela cells (**Fig. 2f**).

In Hela IGF-2R KO cell line, we observed no reduction in EGFR level (**Fig. 2f**). All results above suggest IGF-2R is essential for inducing the degradation for the EndoTag system, and its degradation activity relies on the relative IGF-2R level of the cell lines.

5) The binding selectivity of the various EndoTags is unclear.
We agree with the reviewer that selectivity is essential for the EndoTag platform. Therefore, we generated 2 individual KO cell lines that silenced the Sortilin and TfR, respectively (**Extend Data Fig. 11**) and sourced the Hela IGF-2R KO cell line from Banik lab at Stanford University. We then compared the degradation efficacy using the corresponding EGFRn-EndoTags fusions in both WT and KO cell lines. In the Hela IGF-2R KO cell line, the EGFR degradation is largely ablated after EGFRn-IGF_EndoTag2 treatment (**Fig. 2f**). In the Hela TfR KO cell line, the EGFR degradation of EGFRn-TfR_EndoTag is largely reduced (**Fig. 2c**). In the Hela Sortilin KO cell line, the degradation ability is also largely reduced for EGFRn-Sort_EndoTag (**Fig. 2d**). In summary, the western blot results in KO cell line indicate the EndoTag platform is receptor-specific / receptor-dependent.

Reviewer Reports on the First Revision:

Referee #1 (Remarks to the Author):

Rebuttal to author response for Huang et al from Reviewer #1:

The authors have made a good effort to respond to our comments. It is great to see more head-to-head comparisons and especially the new animal data. This definitely raises the impact. However, there are some must haves that are simply not addressed or brushed aside.

1. The author say:

“Engineering native ligands is possible when ligands are known, but for receptors without a known ligand, the generation of the corresponding agonists to trigger endocytosis is hard. Antibodies/nanobodies can be raised to bind a specific epitope, but it is extremely challenging for antibodies/nanobodies to become agonists due to their relatively large size and poor control of distances and orientations. To bridge this gap, we created this general computational design method that can rationally generate non-existing ligands/agonists to trigger endocytosis specifically with different mechanisms, including controlling conformational change and multivalency. In the IGF_EndoTag example, we showed that we can rationally design two small binding proteins (65aa) at two different sites and then elegantly tune the relative orientations and angles between the two sites within proximity to achieve different orientations, and thus achieve optimal endocytosis efficiency. This rational control of orientations within a small space cannot be done by antibody/nanobody screening.”

First, virtually everyone would acknowledge that antibodies and nanobodies are much easier to generate to targets with known ligands or not. Both methods have the same antigen requirements but antibody generation does not require a structure of the target. The de novo designs require very significant engineering and optimization that are more extensive than typical antibodies. De novo design may seem trivial for this talented protein engineering group and they make efforts to increase use of de novo design, but it is far from a democratic approach that can at this point be widely applied. The authors need to acknowledge this point. Secondly, the notion that the de novo design allows one to trigger specific enodocytic mechanisms that antibodies can’t do is also fallacious. One can build antagonistic antibodies as well as agonistic ones. To make these statements the authors would need to provide more evidence that these designs do things antibodies can’t do. This is an undo a burden for this paper and we would not hold them to it, but if they insist on making claims otherwise they need to provide data that strongly support it. Thirdly, a nanobody (or other selectable domains like DARP-ins) is a 10kDA domain that is not in a different size range or foot-print than these designed helical molecules. There is a lot of literature precedent for linking small domains in bi-paratopic forms that are not different than what is proposed here and the authors to not reference any of this. The Authors in their rebuttal state “We have also included a statement in the “induced conformational change” section of the main text to highlight the unique tunability of EndoTag over conventional antibody/nanobody scaffolds.” The authors must be award that many antibodies have been made that are conditional in their binding properties (ATP, pH, calcium) or masked or conformationally dependent. The authors can easily find these references or we can

provide them if needed. Again, there is no need to over-claim when there's not a significant distinction. Lastly, a key requirement for the de novo design is a structure of the target which naïve selections do not require. The authors should not exaggerate advantages that are not there and should spell out the limitations that are obvious. They lose credibility by not doing so.

2. The authors plead that immunogenicity may not be an issue and try to provide examples: "We agree with the reviewer's points about the immunogenicity of the de novo protein since they are novel proteins whose sequences have not been seen by the immune system before. However, novel sequences may not necessarily lead to high immunogenicity. For example, we did not observe significant weight loss in a mouse study for ATZ-EndoTag, indicating an acceptable safety profile of the EndoTag (Fig. 2m)." Anyone would know that immunogenicity can't be assessed by weight loss and over this short time regime. Perhaps at a minimum they should sample the mouse serum and see if they have raised antibodies to the designed degrader and do so over several months and with numbers of animals that are statistically significant. In fact, if these induced antibodies it could be that the efficacy they observe is due to antibodies being raised to the EndoTag which bind it when bound to the tumor and degrade the tumor by ADCC.

Some protein therapeutics will generate an ADA, but these de novo designed molecules are clearly in a different sequence category and until clear data is presented that they are equivalent (or even better as the authors hope) one simply needs to own up to the uncertainty regarding immunogenicity. This uncertainty is likely the reason these have not been widely adopted by pharma as injectable therapeutics unless they want a vaccine.

The authors also argue that the de novo designed proteins have been in the clinic. This is true. However, simple search of the web shows that NK 201 from Neuleukin has been with-drawn from the clinic as it does not out-perform other IL-2 designs based on the natural ligand. The press release does not specify that immunogenicity was the reason but shows that the de novo design did not make it better than engineered variants of the natural cytokine.
https://www.biospace.com/article/neoleukin-therapeutics-slashes-workforce-drops-de-novo-protein-therapeutic-/. TAK-062 from PvP Biologics is an engineered glutenase recently acquired by Takeda for celiac disease. It is used as an oral drug, not injected, so hardly confirmation that de novo designed injectable drugs lack immunogenicity.
https://www.takeda.com/newsroom/newsreleases/2020/takeda-acquires-pvp-biologics-following-results-of-a--phase-1-study-of-tak-062-kuma062-for-the-treatment-of-celiac-disease/. Lastly, IVX-A12 from Icosavax is a vaccine candidate for RSV based on a VLP and shown to be highly immunogenic by intention https://ir.icosavax.com/news-releases/news-release-details/icosavax-initiates-phase-2-trial-ivx-a12-against-rsv-and-hmpv. These three examples of clinically tested de novo designs are hardly examples to argue for de novo designs being non-immunogenic. This issue is big and remains unresolved. If the authors want to quote these examples they should make clear what they are, and not imply because something is in the clinic they may not be immunogenic.

The authors say "We hypothesize that because designed minibinders are small, stable, and soluble, they may not be taken up and presented on dendritic cells". This is a hypothesis and the untested advantage would apply to any small domain binder (nanobody, DARPin, VHH etc). However, for all these small formats it is very likely they would be made as larger fusions to Fc anyway so as to

increase the half-life, otherwise will require frequent injections or pumps like the BiTE blincyto which pharma desperately wants to move away from.

The authors say they have strategies to reduce immunogenicity. “From our perspective, there are also several strategies to reduce immunogenicity: 1. With the ProteinMPNN [Dauparas, Justas, et al. Science (2022)] tool for protein sequence optimization, we could generate protein sequences with a bias on human-like sequences to potentially reduce the risk of immunogenicity. 2. With the peptide-MHC recognition tool developed by our lab [Motmaen, Amir, et al. PNAS (2022)], we are able to avoid protein sequences that can be recognized by the MHC system. 3. By fusing the EndoTag with established antibodies known to have low therapeutic immunogenicity, we can take advantage of the pharmacological properties of the antibody to shield the EndoTag, given the EndoTag sequence is much shorter relative to the antibody sequence” Again, these are hypothetical and either not tested or incompletely so.

The other arguments regarding immunogenicity are theoretical and imperfect practice. For example it is common practice to scan and remove peptide T-cell epitopes using sparse immunopeptidome data bases in hopes of reducing immunogenicity that may not emerge until post-marketing studies. There are up to 10,000 HLA class I alleles across humans and each has its own unique but often overlapping set of immunopeptidomes. Only a small fraction of these immunopeptidomes (albeit the most abundant ones) have been characterized in detail. At this point immunogenicity needs to be determined empirically and non-native designs are a significant pharma concern.

3. The authors make efforts to see if the IGF-2 EndoTag blocks IGF-2 binding to the M6PR. “Orthogonality of IGF-2R based EndoTag to IGF-2: We appreciate the reviewer's concern about the potential competition of our IGF_EndoTags to IGF-2R since they are designed to share similar binding sites at IGF-2R domain11, even though the IGF_EndoTags were originally designed to be orthogonal to M6P binding sites. We carried out a confocal microscopy assay where we pre-treated the Hela cells with non-labeled EndoTags, then washed and added fluorescence-labeled IGF-2 and lysotracker for co-localization. We showed that the IGF-2 can still internalize and localize with lysosome after IGF_EndoTag pretreatment, and the low affinity IGF_EndoTag4 has no interference on IGF-2 internalization at all, while the high affinity IGF_EndoTag2 has slight interference on IGF-2 lysosome co-localization (Extended Data Fig. 9a-b). This finding suggests that by fine-tuning the binding affinity of EndoTag to the IGF-2R domain11, we can avoid the interference of the native IGF-2 trafficking pathway to the lysosome for its native degradation pathway through IGF-2R.” I may be misinterpreting this data, but it makes the point that the EndoTag CAN compete with the IGF-2 binding site, yet is stated to be orthogonal. Yes, orthogonal to M6P but not IGF-2.

4. “Are the EndoTags recycled or are they degraded? This is a very good question: the fate of designed EndoTags is important to understand as a fully recycled EndoTag could serve as a catalytic degrader like PROTAC with optimal degradation capacity. In a preliminary study looking at IgG clearance with ProteinG-IGF_EndoTag fusion, instead of incubating the fluorescence-labeled IgG and ProteinG-IGF_EndoTag together with the cells (Fig.3a), we pre-treated the Jurkat cells with 1 uM of ProteinG-IGF_EndoTag for 4 h or 24 h (see figure below). We extensively washed the pre-treated cells with PBS 5 times and kept them in new media for an additional 4 h followed by another set of PBS washes to fully remove the surface-bound EndoTag. We then incubated the cells with 50 nM of

fluorescence-labeled IgG for 24 h and ran a flow cytometry assay to evaluate the cellular uptake for IgG. If the EndoTag cannot get recycled, it will not traffic back to the membrane to bring the newly added IgG inside the cells. We compared the recycling behaviors across the 5 unpublished EndoTag candidates sourced from IGF_EndoTag3 (generating Irigid1,4,5,6,8) fused with ProteinG variants ProteinG0919. The flow results below indicated that only Irigid1 possessed the recycling ability to recycle and bring more IgG inside the cells. In contrast, other candidates with >90% sequence similarity didn't recycle, especially Irigid8, which shares a very similar binding affinity to Irigid1. We are still trying to figure out the mechanism of why Irigid1 is unique. Our main hypothesis is that Irigid1 possesses binding to IGF-2R at low pH, while Irigid8 and other designs don't. At low pH, the IGF-2R will have another conformational change to remove the bound ligands [Wang, et al. Science Advance. (2020)], and only the designs that maintain the binding to IGF-2R at low pH can keep such a recycling mechanism. We are continuing to investigate approaches for increasing the recycling of the EndoTags. Are these functionally really better than simple non-degrading binders? Virtually all the experiments are degradation and trafficking experiments, with no functional readout. It is important to show a functional benefit. We value the reviewer's concern about the functional performance of the EndoTag versus simple non-degrading binders. We showed that PD-L1 degrader (ATZ-EndoTag fusion) achieved better anti-tumor performance than PD-L1 inhibitor (ATZ alone) in vivo with significantly improved survival (Fig. 2i-l), which provided a functional benefit of the EndoTag approach than standard non-degrading binders. Additionally, in the cell signaling assay where we ran phosphorylation pERK assay on EGF-stimulated cells pretreated with EGFRn-IGF_EndoTag, EGFRn control, or PBS control, the EGFRn-IGF_EndoTag pretreated cells showed significantly decreased phosphorylated pERK signal compared to the PBS or EGFRn inhibitor alone group, indicating that the degradation of EGFR resulted in stronger functional EGF signaling inhibition than non-degrading inhibitor alone. (Extended Data Fig. 13)." I appreciate the experiments to determine if the degrader is degraded. The experimental approach to test this with multiple washes and trying to observe recycling is complicated. But it is interesting that one design survived and others did not. I think the jury is still out on this point but great that they report the differences in the Endo-Tags and that there is more biology to sort out.

In conclusion, the authors have made a good effort to address the issues raised by this reviewer and two others below. We appreciates the scope of the work and the care of the molecular designs and the new animal data. The authors should address the issues we raise above. We do not believe this is a major break-through in the field but it does add to it in important ways. Editors will need decide if this raises to the level of impact reserved for Nature.

Referee #2 (Remarks to the Author):

In the revised manuscript, to address several thematic concerns that seemed mostly consistent from the collective referees reports, the authors have conducted some new experiments. The main new data is from an in vivo study comparing the antitumour activity of one of their PDL1-IGF endotag against the PD-L1 antibody ATZ alone, in a single mouse model (inoculated subcutaneously with A20 lymphoma cells). While the authors are commended for performing this additional in vivo experiment, this new data falls short of establishing strong in vivo PoC, let alone superiority over current degrading platform, because 1) the effect is minor when compared to the ATZ antibody alone; 2) the induced target degradation (presumed to be as measured at sacrifice at day 21, please clarify) is minimal (EDF 15); 3) no comparison against a different degrading e.g. a PD-L1 LYTAC or KINETAC is shown; 4) the model itself (lymphoma cells in subcutaneous implant) is far from ideal when it comes to studying heamatological malignancies such as lymphomas in vivo, and measuring tumour weight / volume from it not fully relevant. A disseminated model, where lymphoma cells are in blood and express luciferase to monitor via imaging and read-out overall survival would be much more relevant and appropriate. So the new data, while showing use of an EndoTag in vivo, does not offer an ideal PoC in vivo and does not establish that the measured in vivo activity to be degradation-driven.

The other major point was about comparison with current Ab-based degradation platforms e.g. LYTACs. The new data to address this point seemed rather minimal (EDF 9c, which mainly establishes that EndTag does not affect the receptor of the Lytac pathway), and the main data being referenced in the rebuttal which is shown in Fig.2e was already included at first submission. So my other main comment at first review about lack of side-by-side comparison of an EndoTag-based versus conventional LYTAC (or other Ab-based) degraders remains largely unchanged.

I had other two minor comments. First, related to the usefulness of including a non-binding negative control, and on this point the authors provide excellent new data showing the introducing mutations on their receptor ligand that abrogates affinity also largely abrogated the induced protein degradation (new data in new EDF 10).
Second, related to providing more evidence on the kinetics of protein degradation, and on this point the authors have not provided any further data, and the main data being referenced in the rebuttal which is shown in EDF6c and EDF6h, which was already included at first submission. While potentially sufficient on its own, this is a time-course degradation measurement from a single system.

So all in all, the manuscript has not been changed or improved substantially in my opinion from first submission, even though there are indeed new data that to some extent strengthen the claims overall. Together, the negatives that were outlined at my first review have largely remained unaddressed. However, on balance, the positives probably outweigh the negatives and there is indeed a lot of new data added to an already data-heavy paper.

Referee #3 (Remarks to the Author):

In their revised manuscript, the authors have addressed the majority of my concerns. In particular, they have performed an in vivo experiment to demonstrate superior anti-tumor activity and tolerability of anti-PDL1/endotag compared to anti-PDL1 alone.
This is no small feat since few studies have to date showed that cell surface protein degradation of actionable receptors using a novel framework can improve efficacy over more traditional approaches.

They have also admirably addressed the collective comments from all reviewers and added appropriate controls for most experiments.

The manuscript had merit from the outset and the revised version is now on a par with other impactful studies published in Nature.

I recommend publication of this significant work

Author Rebuttals to First Revision:

We thank the reviewers for their time and valuable feedback. We are grateful for the opportunity to refine our work based on the reviewers' invaluable feedback and we wish to address their followup concerns comprehensively in our revised manuscript.

Referees' comments:

Referee #1 (Remarks to the Author):

Rebuttal to author response for Huang et al from Reviewer #1:

The authors have made a good effort to respond to our comments. It is great to see more head-to-head comparisons and especially the new animal data. This definitely raises the impact. However, there are some must haves that are simply not addressed or brushed aside.

1. The author say:

"Engineering native ligands is possible when ligands are known, but for receptors without a known ligand, the generation of the corresponding agonists to trigger endocytosis is hard. Antibodies/nanobodies can be raised to bind a specific epitope, but it is extremely challenging for antibodies/nanobodies to become agonists due to their relatively large size and poor control of distances and orientations. To bridge this gap, we created this general computational design method that can rationally generate non-existing ligands/agonists to trigger endocytosis specifically with different mechanisms, including controlling conformational change and multivalency. In the IGF_EndoTag example, we showed that we can rationally design two small binding proteins (65aa) at two different sites and then elegantly tune the relative orientations and angles between the two sites within proximity to achieve different orientations, and thus achieve optimal endocytosis efficiency. This rational control of orientations within a small space cannot be done by antibody/nanobody screening."

First, virtually everyone would acknowledge that antibodies and nanobodies are much easier to generate to targets with known ligands or not. Both methods have the same antigen requirements but antibody generation does not require a structure of the target. The de novo designs require very significant engineering and optimization that are more extensive than typical antibodies. De novo design may seem trivial for this talented protein engineering group and they make efforts to increase use of de novo design, but it is far from a democratic approach that can at this point be widely applied. The authors need to acknowledge this point.

Secondly, the notion that the de novo design allows one to trigger specific enodocytic mechanisms that antibodies can't do is also fallacious. One can build antagonistic antibodies as well as agonistic ones. To make these statements the authors would need to provide more evidence that these designs do things antibodies can't do. This is an undo a burden for this paper and we would not hold them to it, but if they insist on making claims otherwise they need to provide data that strongly support it. Thirdly, a nanobody (or other selectable domains like DARP-ins) is a 10kDA domain that is not in a different size range or foot-print than these designed helical molecules. There is a lot of literature precedent for linking small domains in bi-paratopic forms that are not different than what is proposed here and the authors to not reference any of this. The Authors in their rebuttal state "We have also included a statement in the "induced conformational change" section of the main text to highlight the unique tunability of EndoTag over conventional antibody/nanobody scaffolds." The authors must be aware that many antibodies have been made that are conditional in their binding properties (ATP, pH, calcium) or masked or conformationally dependent. The authors can easily find these references or we can provide them if needed. Again, there is no need to over-claim when there's not a significant distinction. Lastly, a key requirement for the de novo design is a structure of the target which naïve selections do not require. The authors should not exaggerate advantages that are not there and should spell out the limitations that are obvious. They lose credibility by not doing so.

We thank the reviewer's suggestion about avoiding over-claiming the advantage of EndoTag approach over antibody/nanobody approach.

We acknowledge that the protein design is still not trivial to implement at the current point. It is worth mentioning that the recent development of RosettaFold/RFDiffusion/ProteinMPNN has made significant progress in simplifying the protein design pipeline compared to the Rosetta-based software. We will continuously contribute to developing easy-to-use protein design tools and expanding our communities.

We agree with the reviewer on the fact that many antibodies and nanobodies could achieve agonism with subtle engineering, and that many of them possess tunability and conditional properties, so we removed the following claims in the main text: *"To date there does not exist it has remained challenging to rapidly and rationally a rational and systematic approach completely synthetic and systematic approach to design such orthogonal and synthetic activators of cellular trafficking*" in the introduction paragraph and "*achieving similar tunability with antibodies or*

nanobodies remains a challenge" in the "induced conformational change" section to avoid overclaiming. We have included a section in the Conclusion section of the manuscript to discuss the things-to-improve of the current platform including structure requirement, immunogenicity, and recycling mechanism.

2. The authors plead that immunogenicity may not be an issue and try to provide examples: "We agree with the reviewer's points about the immunogenicity of the de novo protein since they are novel proteins whose sequences have not been seen by the immune system before. However, novel sequences may not necessarily lead to high immunogenicity. For example, we did not observe significant weight loss in a mouse study for ATZ-EndoTag, indicating an acceptable safety profile of the EndoTag (Fig. 2m)." Anyone would know that immunogenicity can't be assessed by weight loss and over this short time regime. Perhaps at a minimum they should sample the mouse serum and see if they have raised antibodies to the designed degrader and do so over several months and with numbers of animals that are statistically significant. In fact, if these induced antibodies it could be that the efficacy they observe is due to antibodies being raised to the EndoTag which bind it when bound to the tumor and degrade the tumor by ADCC.

Some protein therapeutics will generate an ADA, but these de novo designed molecules are clearly in a different sequence category and until clear data is presented that they are equivalent (or even better as the authors hope) one simply needs to own up to the uncertainty regarding immunogenicity. This uncertainty is likely the reason these have not been widely adopted by pharma as injectable therapeutics unless they want a vaccine.

The authors also argue that the de novo designed proteins have been in the clinic. This is true. However, simple search of the web shows that NK 201 from Neuleukin has been with-drawn from the clinic as it does not out-perform other IL-2 designs based on the natural ligand. The press release does not specify that immunogenicity was the reason but shows that the de novo design did not make it better than engineered variants of the natural cytokine. https://www.biospace.com/article/neoleukin-therapeutics-slashes-workforce-drops-de-novo-protein-therapeutic-/. TAK-062 from PvP Biologics is an engineered glutenase recently acquired by Takeda for celiac disease. It is used as an oral drug, not injected, so hardly confirmation that de novo designed injectable drugs lack immunogenicity.
https://www.takeda.com/newsroom/newsreleases/2020/takeda-acquires-pvp-biolo

gics-following-results-of-a--phase-1-study-of-tak-062-kuma062-for-the-treatment-of-celiac-disease/. Lastly, IVX-A12 from Icosavax is a vaccine candidate for RSV based on a VLP and shown to be highly immunogenic by intention https://ir.icosavax.com/news-releases/news-release-details/icosavax-initiates-phase-2-trial-ivx-a12-against-rsv-and-hmpv. These three examples of clinically tested de novo designs are hardly examples to argue for de novo designs being non-immunogenic. This issue is big and remains unresolved. If the authors want to quote these examples they should make clear what they are, and not imply because something is in the clinic they may not be immunogenic.

The authors say “We hypothesize that because designed minibinders are small, stable, and soluble, they may not be taken up and presented on dendritic cells”. This is a hypothesis and the untested advantage would apply to any small domain binder (nanobody, DARPin, VHH etc). However, for all these small formats it is very likely they would be made as larger fusions to Fc anyway so as to increase the half-life, otherwise will require frequent injections or pumps like the BiTE blincyto which pharma desperately wants to move away from.

The authors say they have strategies to reduce immunogenicity. “From our perspective, there are also several strategies to reduce immunogenicity: 1. With the ProteinMPNN [Dauparas, Justas, et al. Science (2022)] tool for protein sequence optimization, we could generate protein sequences with a bias on human-like sequences to potentially reduce the risk of immunogenicity. 2. With the peptide-MHC recognition tool developed by our lab [Motmaen, Amir, et al. PNAS (2022)], we are able to avoid protein sequences that can be recognized by the MHC system. 3. By fusing the EndoTag with established antibodies known to have low therapeutic immunogenicity, we can take advantage of the pharmacological properties of the antibody to shield the EndoTag, given the EndoTag sequence is much shorter relative to the antibody sequence” Again, these are hypothetical and either not tested or incompletely so.

The other arguments regarding immunogenicity are theoretical and imperfect practice. For example it is common practice to scan and remove peptide T-cell epitopes using sparse immunopeptidome data bases in hopes of reducing immunogenicity that may not emerge until post-marketing studies. There are up to 10,000 HLA class I alleles across humans and each has its own unique but often overlapping set of immunopeptidomes. Only a small fraction of these immunopeptidomes (albeit the most abundant ones) have been characterized in detail. At this point immunogenicity needs to be determined empirically and non-native designs are a significant pharma concern.

We appreciate the reviewer’s follow-up comments regarding immunogenicity. We were referring in our original response to the statement in the Neoleukin press release (2022Q3) that: “The decision to drop the candidate is unexpected, as data showed NL-201 engaged the target receptor, performed as expected in terms of pharmacodynamic changes for a potent IL-2/IL-15 agonist and did not demonstrate significant immunogenicity”. We also have a wealth of immunogenicity information in preclinical studies indicating that designs are not greatly immunogenic [Case, James Brett, et al. Cell Host & Microbe 29.7 (2021) Fig5; Chevalier, Aaron, et al. Nature (2017) Fig5]. But the reviewer is certainly correct that this is still an important concern, and a pivotal aspect of therapeutic development, and we understand the critical nature of this evaluation. Additionally, the remarks about the variability of HLA class I alleles and the limitations inherent in current immunopeptidome databases have been well-received. We recognize the cautious approach of the pharmaceutical industry in adopting new therapeutics due to these uncertainties.

In light of the feedback, we have incorporated a new section in the Conclusion section of our revised manuscript. This section will explicitly acknowledge the aforementioned limitations and emphasize that the field of immunogenicity, particularly concerning de novo designed molecules, warrants further investigation through empirical immunogenicity determination assays.

While we are grateful for the reviewer’s comprehensive analysis and valuable suggestions concerning the immunogenicity determination of designed proteins, we wish to clarify that the primary focus of our manuscript is on the protein design aspect of novel endocytosis ligands for extracellular degradation. The aim is not to prove the therapeutic potential of a new degrader platform. Consequently, we believe that additional extensive immunogenicity experiments exceed the current scope of our manuscript. Such detailed investigations would be more suitably addressed in a future paper with a focus on drug development. We hope that our revised manuscript, with the proposed additions and clarifications, will adequately address the reviewer’s concerns while maintaining its focus on the innovative aspect of protein design in our study.

3. The authors make efforts to see if the IGF-2 EndoTag blocks IGF-2 binding to the M6PR. “Orthogonality of IGF-2R based EndoTag to IGF-2: We appreciate the reviewer's concern about the potential competition of our IGF_EndoTags to IGF-2R since they are designed to share similar binding sites at IGF-2R domain11, even though the IGF_EndoTags were originally designed to be orthogonal to M6P binding sites. We carried out a confocal microscopy assay where we pre-treated the Hela cells with non-labeled EndoTags, then washed and added fluorescence-labeled IGF-2 and lysotracker for co-localization. We showed that the IGF-2 can still internalize and localize with lysosome after IGF_EndoTag pretreatment, and the low affinity IGF_EndoTag4 has no interference on IGF-2 internalization at all, while the high affinity IGF_EndoTag2 has slight interference on IGF-2 lysosome co-localization (Extended Data Fig. 9a-b). This finding suggests that by fine-tuning the binding affinity of EndoTag to the IGF-2R domain11, we can avoid the interference of the native IGF-2 trafficking pathway to the lysosome for its native degradation pathway through IGF-2R.” I may be misinterpreting this data, but it makes the point that the EndoTag CAN compete with the IGF-2 binding site, yet is stated to be orthogonal. Yes, orthogonal to M6P but not IGF-2.

We thank the reviewer for bringing this to our attention and allowing us to further clarify. We have edited the text to accurately reflect the results, where EndoTag binds to an orthogonal site from the M6P binding site.

4. “Are the EndoTags recycled or are they degraded? This is a very good question: the fate of designed EndoTags is important to understand as a fully recycled EndoTag could serve as a catalytic degrader like PROTAC with optimal degradation capacity. In a preliminary study looking at IgG clearance with ProteinG-IGF_EndoTag fusion, instead of incubating the fluorescence-labeled IgG and ProteinG-IGF_EndoTag together with the cells (Fig.3a), we pre-treated the Jurkat cells with 1 uM of ProteinG-IGF_EndoTag for 4 h or 24 h (see figure below). We extensively washed the pre-treated cells with PBS 5 times and kept them in new media for an additional 4 h followed by another set of PBS washes to fully remove the surface-bound EndoTag. We then incubated the cells with 50 nM of fluorescence-labeled IgG for 24 h and ran a flow cytometry assay to evaluate the cellular uptake for IgG. If the EndoTag cannot get recycled, it will not traffic back to the membrane to bring the newly added IgG inside the cells. We

compared the recycling behaviors across the 5 unpublished EndoTag candidates sourced from IGF_EndoTag3 (generating Irigid1,4,5,6,8) fused with ProteinG variants ProteinG0919. The flow results below indicated that only Irigid1 possessed the recycling ability to recycle and bring more IgG inside the cells. In contrast, other candidates with >90% sequence similarity didn't recycle, especially Irigid8, which shares a very similar binding affinity to Irigid1. We are still trying to figure out the mechanism of why Irigid1 is unique. Our main hypothesis is that Irigid1 possesses binding to IGF-2R at low pH, while Irigid8 and other designs don't. At low pH, the IGF-2R will have another conformational change to remove the bound ligands [Wang, et al. Science Advance. (2020)], and only the designs that maintain the binding to IGF-2R at low pH can keep such a recycling mechanism. We are continuing to investigate approaches for increasing the recycling of the EndoTags. Are these functionally really better than simple non-degrading binders? Virtually all the experiments are degradation and trafficking experiments, with no functional readout. It is important to show a functional benefit. We value the reviewer's concern about the functional performance of the EndoTag versus simple non-degrading binders. We showed that PD-L1 degrader (ATZ-EndoTag fusion) achieved better anti-tumor performance than PD-L1 inhibitor (ATZ alone) in vivo with significantly improved survival (Fig. 2i-l), which provided a functional benefit of the EndoTag approach than standard non-degrading binders. Additionally, in the cell signaling assay where we ran phosphorylation pERK assay on EGF-stimulated cells pretreated with EGFRn-IGF_EndoTag, EGFRn control, or PBS control, the EGFRn-IGF_EndoTag pretreated cells showed significantly decreased phosphorylated pERK signal compared to the PBS or EGFRn inhibitor alone group, indicating that the degradation of EGFR resulted in stronger functional EGF signaling inhibition than non-degrading inhibitor alone. (Extended Data Fig. 13)." I appreciate the experiments to determine if the degrader is degraded. The experimental approach to test this with multiple washes and trying to observe recycling is complicated. But it is interesting that one design survived and others did not. I think the jury is still out on this point but great that they report the differences in the Endo-Tags and that there is more biology to sort out.

We thank the reviewer's recognition of our efforts. We completely agree that there is more biology to sort out regarding the degrader's recycling. In fact, this will be the focus of our future work, where we will rigorously evaluate the fate of the degrader and engineer other degraders that do not get degraded.

In conclusion, the authors have made a good effort to address the issues raised by this reviewer and two others below. We appreciates the scope of the work and

the care of the molecular designs and the new animal data. The authors should address the issues we raise above. We do not believe this is a major break-through in the field but it does add to it in important ways. Editors will need decide if this raises to the level of impact reserved for Nature.

Referee #2 (Remarks to the Author):

In the revised manuscript, to address several thematic concerns that seemed mostly consistent from the collective referees reports, the authors have conducted some new experiments. The main new data is from an in vivo study comparing the antitumour activity of one of their PDL1-IGF endotag against the PD-L1 antibody ATZ alone, in a single mouse model (inoculated subcutaneously with A20 lymphoma cells). While the authors are commended for performing this additional in vivo experiment, this new data falls short of establishing strong in vivo PoC, let alone superiority over current degrading platform, because 1) the effect is minor when compared to the ATZ antibody alone; 2) the induced target degradation (presumed to be as measured at sacrifice at day 21, please clarify) is minimal (EDF 15); 3) no comparison against a different degrading e.g. a PD-L1 LYTAC or KINETAC is shown; 4) the model itself (lymphoma cells in subcutaneous implant) is far from ideal when it comes to studying heamatological malignancies such as lymphomas in vivo, and measuring tumour weight / volume from it not fully relevant. A disseminated model, where lymphoma cells are in blood and express luciferase to monitor via imaging and read-out overall survival would be much more relevant and appropriate. So the new data, while showing use of an EndoTag in vivo, does not offer an ideal PoC in vivo and does not establish that the measured in vivo activity to be degradation-driven.

The other major point was about comparison with current Ab-based degradation platforms e.g. LYTACs. The new data to address this point seemed rather minimal (EDF 9c, which mainly establishes that EndTag does not affect the receptor of the Lytac pathway), and the main data being referenced in the rebuttal which is shown in Fig.2e was already included at first submission. So my other main comment at first review about lack of side-by-side comparison of an EndoTag-based versus conventional LYTAC (or other Ab-based) degraders remains largely unchanged.

I had other two minor comments. First, related to the usefulness of including a non-binding negative control, and on this point the authors provide excellent new data showing the introducing mutations on their receptor ligand that abrogates

affinity also largely abrogated the induced protein degradation (new data in new EDF 10).
Second, related to providing more evidence on the kinetics of protein degradation, and on this point the authors have not provided any further data, and the main data being referenced in the rebuttal which is shown in EDF6c and EDF6h, which was already included at first submission. While potentially sufficient on its own, this is a time-course degradation measurement from a single system.

So all in all, the manuscript has not been changed or improved substantially in my opinion from first submission, even though there are indeed new data that to some extent strengthen the claims overall. Together, the negatives that were outlined at my first review have largely remained unaddressed. However, on balance, the positives probably outweigh the negatives and there is indeed a lot of new data added to an already data-heavy paper.

We sincerely thank the insightful and constructive comments of the reviewer regarding our updated manuscript and followup comments regarding the in vivo data. We also thank the reviewer's recognition of our efforts in resolving the non-binding negative control issue. To further answer the reviewer's comments:

"1) the effect is minor when compared to the ATZ antibody alone; "

Given that ATZ is a FDA-approved antibody with validated treatment efficacy, we wouldn't expect a much stronger anti-tumor effects of ATZ-EndoTag fusion in vivo, and we think the current data in the manuscript (significantly reduced tumor weight in Fig. 2k and improved survival in Fig. 2l) provides acceptable evidence to support our claim on better effect. We were not able to obtain an antibody that has no effect by itself for which the contrast would likely have been greater.

"2) the induced target degradation (presumed to be as measured at sacrifice at day 21, please clarify) is minimal (EDF 15);"

Yes the induced PD-L1 degradation in vivo is measured at sacrifice at day 21. We have elaborated the description in the legend of Extended Data Fig. 15 in the manuscript and included a quantification of the darkness of the WB band after normalization with b-actin. As shown in the figure, the treatment of ATZ alone caused 50% reduction in PD-L1 level, while the ATZ-EndoTag3/ATZ-EndoTag4 led to 80%~85% reduction in PD-L1 level, which reached the maximum protein degradation level we have seen for cell

surface receptors. So we think the current target degradation data still provides reasonable evidence on PD-L1 degradation effects.

“3) no comparison against a different degrading e.g. a PD-L1 LYTAC or KINETAC is shown; “

To our knowledge there is no published animal data for either of the LYTAC/AbTAC/KineTAC platforms, so the in vivo data is an important milestone for the field. Additionally, we aren't trying to claim EndoTag is better than other platform, instead, we would like to highlight we can make extracellular degraders against given lysosome trafficking receptors (like Sortilin and TfR) and target proteins which was not possible with previous LYTAC by leveraging protein design approaches.

“4) the model itself (lymphoma cells in subcutaneous implant) is far from ideal when it comes to studying heamatological malignancies such as lymphomas in vivo”

We agree with the reviewer that the current in vivo data is not perfect and we acknowledge the reviewer’s comprehensive suggestion on the disseminated lymphoma model. Given that our manuscript is mainly focused on the protein design aspect of novel endocytosis ligands for extracellular degradation instead of proving the therapeutic potential of a new degrader platform, we believe the current in vivo data provides a good starting point for guiding future preclinical research in more relevant diseases model.

Referee #3 (Remarks to the Author):

In their revised manuscript, the authors have addressed the majority of my concerns. In particular, they have performed an in vivo experiment to demonstrate superior anti-tumor activity and tolerability of anti-PDL1/endotag compared to anti-PDL1 alone.
This is no small feat since few studies have to date showed that cell surface protein degradation of actionable receptors using a novel framework can improve efficacy over more traditional approaches.

They have also admirably addressed the collective comments from all reviewers and added appropriate controls for most experiments.

The manuscript had merit from the outset and the revised version is now on a par with other impactful studies published in Nature.

I recommend publication of this significant work

We appreciate the reviewer’s recognition of the novel aspects of our approach in demonstrating the superior anti-tumor activity and tolerability of anti-PDL1/EndoTag. As the reviewer points out, exploring the degradation of actionable receptors using our novel framework is relatively uncharted territory.
